# *C11orf58* (Hero20) Gene Polymorphism: Contribution to Ischemic Stroke Risk and Interactions with Other Heat-Resistant Obscure Chaperones

**DOI:** 10.3390/biomedicines12112603

**Published:** 2024-11-14

**Authors:** Irina Shilenok, Ksenia Kobzeva, Vladislav Soldatov, Alexey Deykin, Olga Bushueva

**Affiliations:** 1 Laboratory of Genomic Research, Research Institute for Genetic and Molecular Epidemiology, Kursk State Medical University, 305041 Kursk, Russia; 2 Division of Neurology, Kursk Emergency Hospital, 305035 Kursk, Russia; 3 Laboratory of Genome Editing for Biomedicine and Animal Health, Belgorod State National Research University, 308015 Belgorod, Russia; 4 Department of Pharmacology and Clinical Pharmacology, Belgorod State National Research University, 308015 Belgorod, Russia; 5 Department of Biology, Medical Genetics and Ecology, Kursk State Medical University, 305041 Kursk, Russia

**Keywords:** stroke, chaperones, heat-resistant obscure, *C11orf58*, Hero20, SNPs, *C19orf53*, *C9orf16*, *SERBP1*, *SERF2*

## Abstract

**Background**: Recently identified Hero proteins, which possess chaperone-like functions, are promising candidates for research into atherosclerosis-related diseases, including ischemic stroke (IS). **Methods**: 2204 Russian subjects (917 IS patients and 1287 controls) were genotyped for fifteen common SNPs in Hero20 gene *C11orf58* using probe-based PCR and the MassArray-4 system. **Results**: Six *C11orf58* SNPs were significantly associated with an increased risk of IS in the overall group (OG) and significantly modified by smoking (SMK) and low fruit/vegetable intake (LFVI): rs10766342 (effect allele (EA) A; P(_OG_ = 0.02; _SMK_ = 0.009; _LFVI_ = 0.04)), rs11024032 (EA T; P(_OG_ = 0.01; _SMK_ = 0.01; _LFVI_ = 0.036)), rs11826990 (EA G; P(_OG_ = 0.007; _SMK_ = 0.004; _LFVI_ = 0.03)), rs3203295 (EA C; P(_OG_ = 0.016; _SMK_ = 0.01; _LFVI_ = 0.04)), rs10832676 (EA G; P(_OG_ = 0.006; _SMK_ = 0.002; _LFVI_ = 0.01)), rs4757429 (EA T; P(_OG_ = 0.02; _SMK_ = 0.04; _LFVI_ = 0.04)). The top ten intergenic interactions of Hero genes (two-, three-, and four-locus models) involved exclusively polymorphic loci of *C11orf58* and *C19orf53* and were characterized by synergic and additive (independent) effects between SNPs. **Conclusions**: Thus, *C11orf58* gene polymorphism represents a major risk factor for IS. Bioinformatic analysis showed the involvement of *C11orf58* SNPs in molecular mechanisms of IS mediated by their role in the regulation of redox homeostasis, inflammation, vascular remodeling, apoptosis, vasculogenesis, neurogenesis, lipid metabolism, proteostasis, hypoxia, cell signaling, and stress response. In terms of intergenic interactions, *C11orf58* interacts most closely with *C19orf53*.

## 1. Introduction

Atherosclerosis is the leading cause of ischemic brain injury and acute cerebrovascular events [1,2]. The development of atherosclerotic plaques involves profound alterations in both the composition and function of the cellular compartment within the vascular wall [3], including changes in cellular structure and metabolic function [4]. These modifications are characterized by endothelial cell dysfunction [5], monocyte adhesion [6], followed by their differentiation into macrophages and foam cells [7], and the activation of the secretory and migratory potential of vascular smooth muscle cells (VSMCs) [8,9,10,11]. The primary drivers of these pathological changes include chronic inflammation [12], elevated oxidative stress [13], and hypoxia [14], which increase cellular toxicity, promote vascular calcification, and degrade the extracellular matrix [15,16]. These processes are crucial for the destabilization of atherosclerotic plaques [17], triggering atherothrombosis and, ultimately, ischemic stroke (IS).

Understanding the mechanisms underlying atherosclerosis is essential for developing effective interventions. Much promising research to tackle atherogenesis addressed the role of chaperones, molecular assistants of protein folding, refolding, and degradation. For instance, some recent studies revealed chaperone-mediated autophagy (CMA) as an important player in atherosclerosis [18,19,20,21]. CMA plays a crucial role in responding to stress by being activated during conditions such as starvation, hypoxia, and oxidative stress [22,23,24,25,26], as well as in response to lipotoxicity, proteotoxicity, or DNA damage [27]. CMA selectively degrades damaged proteins to maintain quality control, as well as non-damaged proteins to terminate their function, thereby regulating a variety of intracellular processes, including T-cell activation, cell cycle progression, metabolism, growth, and survival [28,29,30,31,32,33].

Studies have shown that impaired CMA can accelerate the progression of atherosclerosis by influencing macrophages and VSMCs, which are responsible for plaque formation [19,34]. However, not only their role in CMA but also their role in hormonal signaling [35,36], inflammation [37,38], antioxidant defense [39], and especially in the regulation of altered proteomes [40], makes chaperones an important link in atherosclerosis [41].

Given the pivotal role of chaperones, we aimed to address the link between ischemic stroke risk and the recently discovered class of chaperones “Hero” (heat-resistant obscure) [42]. The term “Hero” also draws from its Japanese meaning, “fragile, loose, or flexible”, reflecting the unique physicochemical properties of these proteins. While their functions are still not fully understood, it is now recognized that Hero proteins act as molecular shields, safeguarding various “client” proteins from denaturation during stressful conditions such as heat shock, desiccation, and exposure to harmful chemicals [42]. This function is provided by the flexible structure and high negative or positive charge of the molecules [42]. Unlike molecular chaperones, which often use ATP binding and hydrolysis cycles [43], Hero proteins lack any visible ATPase or other domains. It is suggested that anti-aggregation may be a common feature of thermostable Hero proteins. Additionally, they have been shown to suppress certain types of pathogenic protein aggregates in cells [44], and their functional targeting depends on the type of proteotoxic stress [42]. These characteristics suggest that Hero proteins may play a significant role in both normal cellular processes and disease states. However, their involvement in atherosclerosis-related diseases remains largely unexplored.

In our prior research, we investigated the impact of four Hero genes—*C9orf16* (Hero9) [45], *SERBP1* (Hero45) [46], *SERF2* (Hero7) [47], and *C19orf53* (Hero11) [48]—on ischemic stroke risk, finding a notable association between these genes and the disease.

In this pilot study, we extend our investigation to the *C11orf58* (*SMAP*) gene, which encodes the Hero20 protein, exploring its potential role in the risk and clinical progression of IS. Moreover, we aimed to discover the most significant gene-gene and gene-environmental interactions of the mentioned Hero genes associated with IS risk.

## 2. Materials and Methods

**Figure 1:**
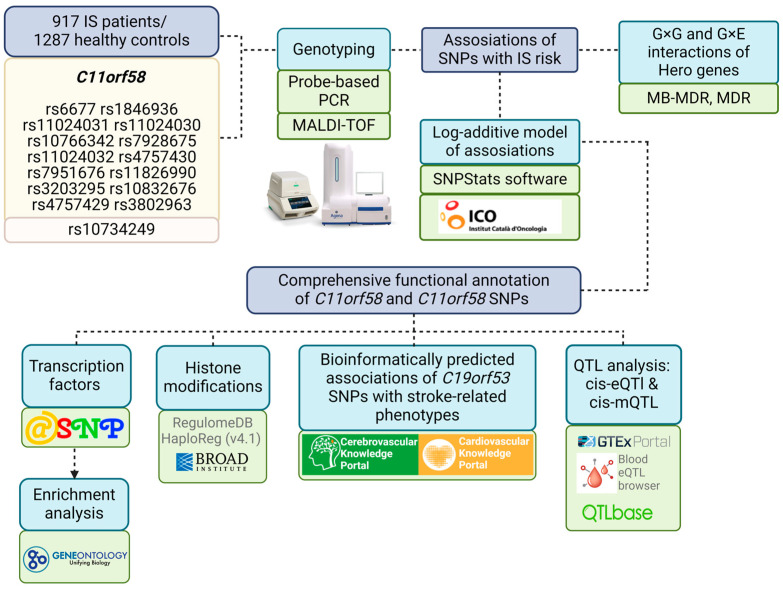
Materials and methods of the study.

Figure 1 provides an overview of the materials and methods used in the research.

### 2.1. Study Participants

The study included 2204 unrelated individuals from Central Russia, comprising 917 IS patients and 1287 healthy controls. The Ethical Review Committee of Kursk State Medical University approved the study protocol, and all participants provided written informed consent.

Table 1 provides the baseline and clinical characteristics of the study cohort.

Patients were recruited for the study in two distinct time frames: from 2015 to 2017 at the Regional Vascular Center of Kursk Regional Clinical Hospital [49] and from 2010 to 2012 at the Neurology Clinics of Kursk Emergency Medicine Hospital [50,51]. Each case underwent evaluation by a team of qualified neurologists. Brain computed tomography and/or magnetic resonance imaging scans were analyzed to confirm an IS diagnosis during the acute period. Our study excluded participants with autoimmune or oncological diseases, traumatic brain injury, hemodynamic or dissection-related stroke, intracerebral hemorrhage, hepatic or renal failure, and other conditions that could cause an abrupt cerebrovascular event. Every IS patient was being treated with antihypertensive drugs and had a history of hypertension that was documented.

Individuals in the control group did not have clinical symptoms of cardiovascular, cerebrovascular, or other serious disorders. They also did not take antihypertensive medication and had normal blood pressure. Control subjects were selected during regular medical examinations in public and industrial establishments in the Kursk region [52,53]. The same population and time period were used to choose this group. Consuming fewer than 400 g of fresh fruit and vegetables equivalent to 3–4 servings per day, excluding starchy tubers such as potatoes, was considered a deficient intake, in accordance with the World Health Organization’s recommendations [54].

### 2.2. Selection of SNPs

In Table S1, we provide the general characteristics of Hero genes, including *C11orf58*, which has three identified mRNA transcripts (*NM_014267.6*, *XM_017017143.3*, and *XM_047426310.1*) and three corresponding protein isoforms (*NP_055082.1*, *XP_016872632.1*, and *XP_047282266.1*). Among these, the isoform *NP_055082.1* consists of 183 amino acids and has a molecular weight of 20,333 Daltons (Da), as detailed in Table S1.

SNPs were selected based on the criteria of having a minor allele frequency of at least 0.05 in the European population and exhibiting a high regulatory potential.

The bioinformatic tools LD TAG SNP Selection (TagSNP) and SNPinfo Web Server (https://snpinfo.niehs.nih.gov/ (accessed on 15 March 2022)) were utilized to choose SNPs based on the reference haplotypic structure of the Caucasian population (CEU) of the project HapMap. We selected fifteen SNPs in the *C11orf58* (Chromosome 11 Open Reading Frame 58, ID:10944) gene: SNPs rs11024031, rs10766342, rs7928675, rs11024030, rs11024032, rs4757430, rs7951676, rs11826990, rs3203295, rs10832676, rs4757429, rs3802963, and rs10734249 are located in introns, rs6677 is located in the 3 prime UTRs, and rs1846936 is located in the 5 prime UTR (Table S2).

### 2.3. Genetic Analysis

A genetic study was conducted by the Laboratory of Genomic Research at the Research Institute for Genetic and Molecular Epidemiology of Kursk State Medical University (Kursk, Russia). Each participant’s cubital vein was used to draw up to 5 mL of venous blood, which was then preserved in tubes coated with EDTA and kept at −20 °C until processing. Genomic DNA was extracted using standard phenol/chloroform extraction. A NanoDrop spectrophotometer (Thermo Fisher Scientific, Waltham, MA, USA) was then used to assess the extracted DNA samples for purity, quality, and concentration.

The Laboratory of Genomic Research at the Research Institute for Genetic and Molecular Epidemiology of Kursk State Medical University designed protocols for allele-specific probe-based polymerase chain reaction (PCR) that were used to genotype the SNPs rs6677, rs1846936, rs11024031, rs10766342, rs7928675, rs11024030, rs11024032, rs4757430, rs7951676, rs11826990, rs3203295, rs10832676, rs4757429, and rs3802963. The Primer3 program [55] was used to create the primers, and Table S3 lists the primer and probe sequences that were used for genotyping. A 25 µL reaction solution was used for a real-time PCR procedure. It contained 1.5 units of Hot Start Taq DNA polymerase (Biolabmix, Novosibirsk, Russia), roughly 10 ng of DNA, 0.25 μM of each primer, 0.1 μM of each probe, 250 μM of each dNTP, and 1xPCR buffer (67 mM Tris-HCl, pH 8.8, 16.6 mM (NH_4_)_2_SO_4_, 0.01% Tween-20) and the following concentrations of MgCl2: 1.5 mM MgCl_2_ (rs4757430) or 2 mM MgCl_2_ (rs11024031) or 2.5 mM MgCl_2_ (rs1846936, rs11024032, rs3802963, rs3203295, rs11024030) or 3 mM MgCl_2_ (rs6677, rs7928675, rs11826990, rs4757429, rs10832676) or 3.5 mM MgCl_2_ (rs10766342) or 4 mM MgCl_2_ (rs7951676). To ensure quality control, 10% of the DNA samples underwent additional genotyping while being blinded to the case control status. A full 99% of the data were in agreement.

The MassARRAY-4 genetic analyzer (Agena Bioscience, San Diego, CA, USA) was used to genotype the SNP rs10734249. The Assay Design Suite from Agena Bioscience was utilized to create SNP primers, and matrix-assisted laser desorption ionization mass spectrometry (MALDI-TOF) was used for genotyping; the methodology is described in detail in our previous study [56]. Using prawn alkaline phosphatase (SAP), unreacted dNTPs were eliminated following locus-specific PCR. A further iPLEX reaction required E-primer mixing, and the resultant mixture underwent mass spectrometry. Contamination peaks were avoided by using SpectroCLEAN resin (Agena Bioscience, San Diego, CA, USA) in robotic desalination. Samples were put onto a spectral chip, exposed to a high-energy laser, and then TyperAnalyzer was used to determine the genotype of the samples. Ninety-five randomly chosen DNA samples were genotyped again on the same platform for quality assurance, but the case-control status was kept unknown. The test for repeatability showed 100% concordance.

### 2.4. Statistical and Bioinformatic Analysis

The statistical power was calculated using the genetic association study power calculator, which may be accessed online at http://csg.sph.umich.edu/abecasis/gas_power_calculator/ (accessed on 15 March 2022). With a sample size of 1287 controls and 917 cases, the association analysis of *C11orf58* gene SNPs and IS risk was able to detect a genotype relative risk between 1.47 and 1.55, assuming 0.80 power and a 5% type I error (α = 0.05). The minor allele frequencies ranged from 0.166 to 0.468. The STATISTICA program (v13.3, USA) was used to perform the statistical analysis. Most quantitative characteristics were expressed as the median (Me) with the first and third quartiles [Q1 and Q3] due to their divergence from normal distribution. For quantitative variables comparing two independent groups, the Mann–Whitney test was utilized, and for categorical variables, Pearson’s chi-squared test with Yates’s correction for continuity was applied. The Hardy-Weinberg equilibrium compliance of genotype distributions was evaluated by means of Fisher’s exact test.

Associations of genotypes and haplotypes with disease risk, as well as linkage disequilibrium analysis, were performed through SNPStats software version 4.1.0 [57]. For haplotype analysis, only tagging SNPs (a representative single nucleotide polymorphism (SNP) that indicates a group of SNPs known as a haplotype in a section of the genome with strong linkage disequilibrium) were taken into account. The genotype association analysis considered an additive model. Age, gender, and smoking status adjustments were made to the associations as these covariates showed significant differences between the control and IS patient groups. Recognizing that multifactorial diseases often exhibit pronounced sexual dimorphism, we also conducted separate association analyses within gender-specific subgroups. Furthermore, to understand how environmental risk factors may modify the associations of the SNPs under study with IS risk, we performed analyses in subgroups defined by the presence or absence of these risk factors.

Recognizing that multifactorial diseases often exhibit pronounced sexual dimorphism, we also conducted separate association analyses within gender-specific subgroups. In order to assess how environmental risk factors may modify the associations of the studied SNPs with IS risk, we performed analyses in subgroups defined by the presence or absence of these risk factors. In subgroup analyses based on environmental risk factors, in cases where information on the environmental risk factor in the control group was missing, associations were analyzed based on the presence or absence of the risk factor in the patient group compared with the overall control group. The Bonferroni adjustment was used in these cases.

The MB-MDR analysis tested two-, three-, and four-level genotype combinations (G × G) and genotype-environment combinations (G × E). Since there were differences in this environmental risk factor between the patient and control groups and because smoking has a high pathogenetic significance in the IS risk, smoking was examined as an environmental risk factor in the analysis of G × E interactions. A permutation test was used to estimate the empirical *p*-value (Pperm) for each G × G and G × E model and to improve the validity of the results obtained. Because the default call to MB-MDR is designed to simultaneously test all possible interactions of a given order, we used 1000 permutations to obtain accurate *p*-values. Models (on average 3–4 models of each level) with the highest Wald statistics and the lowest P-level of significance (Pperm < 0.001) were considered statistically significant and used for further analysis. Additionally, using the MB-MDR method, individual combinations of genotypes associated with the studied phenotypes were established (*p* < 0.05). The calculation was performed with the R programming (Version 3.6.3) language.

Furthermore, the MDR approach was used to analyze the most important G × G and G × E models. Genes included in the two or more best 2-, 3-, and 4-locus G × G-models, as well as genes included in the two or more best 2-, 3-, and 4-locus G × E-models, were included in the MDR testing. This analysis was applied in version 3.0.2 of the MDR program (http://sourceforge.net/projects/mdr (accessed on 8 October 2024)). The interactions’ mechanisms (synergy, antagonism, additive interactions (independent effects)) and strengths (the contribution of interactions and the individual genes’ and environmental factors’ entropy of a characteristic) were evaluated using the MDR approach [58]. The results of the MDR analysis were visualized as a graph.

The following bioinformatics resources were used for bioinformatics analysis of the functional effects of the *C11orf58* gene and *C11orf58* SNPs (the approaches used are described in detail in our previous studies [59,60] (Table S4):

- The GTExportal was used for analysis of cis-eQTL (expression quantitative trait loci)-effects of *C11orf58* SNPs and for the assessments of expression levels of the studied gene in the vessels, brain, and whole blood [61].
- Extras examination of *C11orf58* SNPs eQTLs in peripheral blood, the eQTLGen resource was employed [62].
- We used QTLbase to look at the mQTLs (methylation quantitative trait loci) [63].
- To evaluate the relationships between *C11orf58* SNPs and particular histone modifications indicative of promoters and enhancers and positioning of SNPs in DNase hypersensitive regions, HaploReg (v4.2) was used [64]. These modifications included the lysine residues at locations 27 and 9 of the histone H3 protein being acetylated (H3K27ac and H3K9ac), as well as the mono-methylation and tri-methylation of the 4th lysine residue of the histone H3 protein (H3K4me1 and H3K4me3, respectively).
- The effect of *C11orf58* SNPs on the gene’s affinity for transcription factors (TFs) based on the carriage of reference and alternative alleles was assessed using the atSNP Function Prediction tool [65].
- In order to analyze the combined participation of TFs connected to reference/SNP alleles in over-represented biological processes directly associated to the molecular mechanisms of IS, Gene Ontology was recruited [66].
- Bioinformatic analyses of the associations of *C11orf58* SNPs with cerebrovascular diseases and risk factors for IS (such as total cholesterol, LDL, BMI, etc.) were conducted using the tools available on the Cerebrovascular Disease Knowledge Portal (CDKP) and Cardiovascular Disease Knowledge Portal (CVDKP), which aggregate and evaluate the findings of genetic associations of the largest consortiums for the study of cardio- and cerebrovascular diseases [67].

## 3. Results

### 3.1. C11orf58 SNPs and the Ischemic Stroke Risk: An Analysis of Associations

The genotype frequencies of *C11orf58* SNPs (rs6677, rs1846936, rs11024031, rs10766342, rs7928675, rs11024030, rs11024032, rs4757430, rs7951676, rs11826990, rs3203295, rs10832676, rs4757429, rs3802963, rs10734249) in the study groups are presented in Table S5. The distribution of the genotype frequencies of all studied SNPs corresponded to the Hardy-Weinberg equilibrium in both the control and case groups (*p* > 0.05).

The analysis conducted on the total sample revealed significant associations between *C11orf58* following SNPs and IS risk: rs10766342 (risk allele A; OR = 1.21, 95% CI 1.03–1.43, *p* = 0.02), rs11024032 (risk allele T; OR = 1.22, 95% CI 1.04–1.44, *p* = 0.01), rs11826990 (risk allele G; OR = 1.25, 95% CI 1.06–1.47, *p* = 0.007), rs3203295 (risk allele C; OR = 1.22, 95% CI 1.04–1.44, *p* = 0.016), rs10832676 (risk allele G; OR = 1.26, 95% CI 1.07–1.48, *p* = 0.006), rs4757429 (risk allele T; OR = 1.21, 95% CI 1.03–1.42, *p* = 0.02) (Table 2 and Table S6).

The data reveal associations between specific genetic variants of *C11orf58* and the risk of IS under two different conditions: smoking and low fruit and vegetable intake (Table 2 and Table S7).

In smokers, the following genetic variants were found to be associated with an increased risk of IS: rs10766342 (risk allele A; OR = 1.42; 95% CI = 1.09–1.86; *p* = 0.009), rs11024030 (risk allele C; OR = 1.33; 95% CI = 1.02–1.72; *p* = 0.03), rs11024032 (risk allele T; OR = 1.39; 95% CI = 1.07–1.81; *p* = 0.01), rs11826990 (risk allele G; OR = 1.48; 95% CI = 1.13–1.93; *p* = 0.004), rs3203295 (risk allele C; OR = 1.40; 95% CI = 1.07–1.83; *p* = 0.01), rs10832676 (risk allele G; OR = 1.53; 95% CI = 1.17–2.00; *p* = 0.002), and rs4757429 (risk allele T; OR = 1.32; 95% CI = 1.01–1.71; *p* = 0.04) (Table 2 and Table S7). Meanwhile, rs7928675 (protective allele C; OR = 0.73; 95% CI = 0.56–0.96; *p* = 0.025) and rs7951676 (protective allele T; OR = 0.75; 95% CI = 0.57–0.99; *p* = 0.04) were associated with a reduced risk of IS among smokers (Table 2 and Table S7).

On the other hand, under the condition of low fruit and vegetable intake (Table 2 and Table S7), with an increased risk of IS were associated: rs10766342 (risk allele A; OR = 1.26; 95% CI = 1.04–1.53; *p* = 0.02, P_bonf_ = 0.04), rs11024030 (risk allele C; OR = 1.26; 95% CI = 1.04–1.52; *p* = 0.018, P_bonf_ = 0.032), rs11024032 (risk allele T; OR = 1.26; 95% CI = 1.04–1.52; *p* = 0.018, P_bonf_ = 0.036), rs11826990 (risk allele G; OR = 1.26; 95% CI = 1.05–1.53; *p* = 0.017, P_bonf_ = 0.034), rs3203295 (risk allele C; OR = 1.26; 95% CI = 1.04–1.53; *p* = 0.02, P_bonf_ = 0.04), rs10832676 (risk allele G; OR = 1.30; 95% CI = 1.07–1.57; *p* = 0.007, P_bonf_ = 0.014), and rs4757429 (risk allele T; OR = 1.25; 95% CI = 1.03–1.51; *p* = 0.02, P_bonf_ = 0.04) (Table 2 and Table S7). The relationships between IS risk and the haplotypes of the tag SNPs were discovered by analysis (Table S8). The haplotype rs3802963G-rs7951676G-rs11024032T-rs6677T *C11orf58* was linked to an increased risk of IS in the entire group (OR = 1.22; 95% CI = 1.02–1.46; *p* = 0.026) and in smokers (OR = 1.34; 95% CI = 1.01–1.79; *p* = 0.04). Conversely, the haplotype rs3802963G-rs7951676T-rs11024032C-rs6677T *C11orf58* was associated with a reduced risk of IS in smokers (OR = 0.71; 95% CI = 0.52–0.95; *p* = 0.022). The haplotype rs3802963G-rs7951676G-rs11024032C-rs6677T *C11orf58* was associated with an increased risk of IS in non-smokers (OR = 1.90; 95% CI = 1.14–3.19; *p* = 0.015). Notably, in patients with low fruit and vegetable intake, haplotypes rs3802963G-rs7951676G-rs11024032C-rs6677T *C11orf58* (OR = 1.61; 95% CI = 1.00–2.57; *p* = 0.048) and rs3802963C-rs7951676T-rs11024032C-rs6677T *C11orf58* (OR = 2.11; 95% CI = 1.00–4.46; *p* = 0.049) showed borderline associations with increased IS risk.

Since divergent effects of SNPs on the risk of IS in smokers have been found, we have assessed the linkage disequilibrium between IS-associated *C11orf58* SNPs (Table S9). It turned out that the protective SNP rs7928675 is in positive linkage disequilibrium with the protective SNP rs7951676 and in negative linkage disequilibrium with the risk SNPs rs4757429, rs10832676, rs11024030, rs11024032. The protective SNP rs7951676 is negatively linked with the risk SNPs rs11024030 and rs11024032.

### 3.2. Functional Annotation of IS-Associated C11orf58 SNPs

The *C11orf58* gene is expressed in brain tissues, blood vessels, and whole blood. *C11orf58* gene expression levels (MeTPM) range from 37.12 to 89.32 in brain tissues, from 120.8 to 147.5 in blood vessels, and are 19.04 in whole blood (Figure S1).

#### 3.2.1. Quantitative Trait Loci (QTL) Analysis

Table 3 displays the results of the cis-eQTL analysis involving the *C11orf58* SNPs. According to the data from the eQTLGene browser, SNPs linked to an increased risk of IS, namely rs10766342 (effect allele A), rs11024030 (effect allele C), rs11024032 (effect allele T), rs11826990 (effect allele G), rs3203295 (effect allele C), rs10832676 (effect allele G), and rs4757429 (effect allele T), were associated with decreased expression levels of both *C11orf58* and *PIK3C2A* in blood. Conversely, the SNPs associated with a decreased risk of IS, specifically rs7928675 (effect allele C) and rs7951676 (effect allele T), were associated with reduced expression of *C11orf58* in blood (Table 3).

Table 4 presents an analysis of the cis-mQTL effects of the *C11orf58* SNPs. It was observed that all risk-associated SNPs within *C11orf58* were associated with elevated methylation levels of cg05512310, cg05405872, cg20474675, and cg01047635 within the *C11orf58* gene in the brain-prefrontal cortex, resulting in decreased expression of *C11orf58* (Table 4).

#### 3.2.2. Histone Modifications

Upon further examination, we have uncovered the significant impact of *C11orf58* SNPs associated with IS on histone modifications (Table 5).

Specifically, all IS-associated SNPs are located in the region of DNA binding to H3K4me1. Furthermore, SNPs rs10766342, rs11826990, rs3203295, rs4757429, and rs7928675 are situated in the region of DNA binding to H3K4me3 in both brain tissues and blood.

The influence of these histone modifications is increased by the H3K27ac marking enhancers in blood cells and brain tissues for SNPs rs10766342, rs11024032, rs11826990, rs3203295, rs4757429, and rs7928675. Additionally, SNPs rs10766342, rs11024032, rs11826990, rs3203295, and rs7928675 are further influenced by H3K9ac, which marks enhancers, in the brain tissues, while the same happens for the SNPs rs10766342, rs11826990, rs3203295, rs4757429, rs7928675, and rs79516760 in blood.

Notably, SNPs rs10832676 and rs7951676 are exclusively found within the regions of DNA binding to H3K4me3 in blood, and the effect of these histone tags is increased by the H3K27ac.

Moreover, the SNPs rs10766342, rs11826990, and rs3203295 are also situated in DNA regions that are hypersensitive to DNase-1 in the blood (Table 5).

#### 3.2.3. Analysis of Transcription Factors

The genetic variants associated with stroke risk and *C11orf58* reveal diverse impacts on transcription factor (TF) binding and biological processes (Tables S10–S18). Protective allele G of rs10766342 creates DNA binding sites for 11 TFs primarily involved in astrocyte fate commitment (GO:0060018; FDR = 0.0027). In contrast, the risk allele A of rs10766342 generates binding sites for 84 TFs implicated in 14 overrepresented GOs, encompassing neuronal cell differentiation and development: neuron fate specification (GO:0048665; FDR = 0.00031), dopaminergic neuron differentiation (GO:0071542; FDR = 0.0076), positive regulation of neuron differentiation (GO:0045666; FDR = 0.0073), neural precursor cell proliferation (GO:0061351; FDR = 0.01), oligodendrocyte differentiation (GO:0048709; FDR = 0.047), regulation of neurogenesis (GO:0050767; FDR = 0.026), astrocyte fate commitment (GO:0060018; FDR = 0.0058), axon development (GO:0061564; FDR = 0.04); vascular and smooth muscle cell regulation: artery morphogenesis (GO:0048844; FDR = 0.0018), regulation of vascular associated smooth muscle cell proliferation (GO:1904705; FDR = 0.028), negative regulation of angiogenesis (GO:001652; FDR = 0.0096); immune response and inflammation: monocyte differentiation (GO:0030224; FDR = 0.047), response to cytokine (GO:0034097; FDR = 0.017) and cellular response to transforming growth factor beta stimulus (GO:0071560; FDR = 0.033) (Table S10).

Moving on to the SNP rs11024030 *C11orf58*, the risk allele C creates DNA binding domains for 88 TFs, associated with neuronal differentiation and development: negative regulation of gliogenesis (GO:0014014; FDR = 0.0164), neuron differentiation (GO:0030182; FDR = 0.00885), positive regulation of neuron differentiation (GO:0045666; FDR = 0.0000336); stress response: cellular response to stress (GO:0033554; FDR = 0.0166); response to hypoxia (GO:0001666; FDR = 0.00788) and response to oxidative stress (GO:0006979; FDR = 0.03); neuronal apoptosis: positive regulation of neuron apoptotic process (GO:0043525; FDR = 0.0339), negative regulation of fat cell proliferation (GO:0008285; FDR = 0.00817) (Table S11).

The risk allele T of rs11024032 *C11orf58* generates binding sites on DNA for 26 TFs, impacting biological processes such as positive regulation of leukocyte adhesion to vascular endothelial cells (GO:1904996; FDR = 0.0393), positive regulation of NIK/NF-kappaB signaling (GO:1901224; FDR = 0.0109), and positive regulation of cytokine production (GO:0001819; FDR = 0.0217) (Table S12).

For the risk allele G of rs11826990 *C11orf58*, it is responsible for generating DNA binding domains for 50 TFs involved in multiple biological processes, including positive regulation of neuron differentiation (GO:0045666; FDR = 0.0000746), neuron development (GO:0048666; FDR = 0.00364), regulation of blood vessel endothelial cell migration (GO:0043535; FDR = 0.0324), positive regulation of endothelial cell migration (GO:0010595; FDR = 0.0466), regulation of interleukin-2 production (GO:0032663; FDR = 0.0135), regulation of cellular response to growth factor stimulus (GO:0090287; FDR = 0.0103), and negative regulation of apoptotic process (GO:0043066; FDR = 0.0248) (Table S13).

Conversely, protective allele A of rs3203295 *C11orf58* creates DNA binding sites for 17 TFs specifically implicated in the regulation of monocyte differentiation (GO:0045655; FDR = 0.0237). In contrast, risk allele C of rs3203295 generates binding sites for 38 TFs associated with neuron apoptotic process (GO:0051402; FDR = 0.035), regulation of neuron apoptotic process (GO:0043523; FDR = 0.0204), positive regulation of apoptotic process (GO:0043065; FDR = 0.0369) and nervous system development (GO:0007399; FDR = 0.000346) (Table S14).

The risk allele G of rs10832676 *C11orf58* establishes binding sites on DNA for 100 TFs (Table S15). These TFs are involved in several biological processes, including transcriptional regulation in hypoxia and oxidative stress: regulation of transcription from RNA polymerase II promoter in response to oxidative stress (GO:0043619; FDR = 0.000369), positive regulation of transcription from RNA polymerase II promoter in response to hypoxia (GO:0061419; FDR = 0.0103); response to oxidative stress and cellular damage: oxidative stress-induced premature senescence (GO:0090403; FDR = 0.00787), negative regulation of reactive oxygen species metabolic process (GO:2000378; FDR = 0.00127), positive regulation of transcription from RNA polymerase II promoter in response to endoplasmic reticulum stress (GO:1990440; FDR = 0.0333), regulation of autophagy of mitochondrion (GO:1903146; FDR = 0.0136); vascular differentiation and development: arterial endothelial cell differentiation (GO:0060842; FDR = 0.0128), positive regulation of vascular endothelial growth factor receptor signaling pathway (GO:0030949; FDR = 0.0415), positive regulation of vascular endothelial growth factor production (GO:0010575; FDR = 0.00769), regulation of vascular associated smooth muscle cell proliferation (GO:1904705; FDR = 0.0432); neurogenesis: neuron differentiation (GO:0030182; FDR = 0.00239), negative regulation of gliogenesis (GO:0014014; FDR = 0.0174); signaling pathways: cellular response to growth factor stimulus (GO:0071363; FDR = 0.0333), negative regulation of transforming growth factor beta receptor signaling pathway (GO:0030512; FDR = 0.00124); cell death and survival mechanisms: regulation of apoptotic process (GO:0042981; FDR = 0.0000166), positive regulation of apoptotic process (GO:0043065; FDR = 0.00258), negative regulation of apoptotic process (GO:0043066; FDR = 0.00658), negative regulation of Notch signaling pathway (GO:0045746; FDR = 0.0146); metabolic response and lipid metabolism: peroxisome proliferator activated receptor signaling pathway (GO:0035357; FDR = 0.0218); proteostasis: positive regulation of protein acetylation (GO:1901985; FDR = 0.0102) (Table S15).

For the risk allele T of rs4757429, it creates binding sites for 86 TFs, playing roles in the vascular remodeling: blood vessel remodeling (GO:0001974; FDR = 0.016), artery morphogenesis (GO:0048844; FDR = 0.000365); neural development and function: neuron fate specification (GO:0048665; FDR = 0.0125), peripheral nervous system development (GO:0007422; FDR = 0.000653), positive regulation of neurogenesis (GO:0050769; FDR = 0.0434); hypoxia response: positive regulation of transcription from RNA polymerase II promoter in response to hypoxia (GO:0061419; FDR = 0.0126); stress response: integrated stress response signaling (GO:0140467; FDR = 0.000515); apoptosis regulation: negative regulation of stress-activated MAPK cascade (GO:0032873; FDR = 0.0291), Notch signaling pathway (GO:0007219; FDR = 0.00278). Conversely, the protective allele C of rs4757429 results in binding sites for 32 TFs implicated in neurogenesis: neuron differentiation (GO:0030182; FDR = 0.0226); sell signaling: BMP signaling pathway (GO:0030509; FDR = 0.0271); apoptosis: regulation of neuron apoptotic process (GO:0043523; FDR = 0.0207); positive regulation of programmed cell death (GO:0043068; FDR = 0.0425); inflammation: positive regulation of cytokine production (GO:0001819; FDR = 0.0311) (Table S16).

With the presence of the protective allele C of rs7928675 DNA binding sites for 65 TFs are formed (Table S17). All together these TFs represent cellular responses and signaling: cellular response to growth factor stimulus (GO:0071363; FDR = 0.0442), transmembrane receptor protein serine/threonine kinase signaling pathway (GO:0007178; FDR = 0.000995); inflammation: interleukin-6-mediated signaling pathway (GO:0070102; FDR = 0.0437), cellular response to interleukin-17 (GO:0097398; FDR = 0.0476), regulation of apoptosis: positive regulation of apoptotic process (GO:0043065; FDR = 0.0116); hypoxia: cellular response to hypoxia (GO:0071456; FDR = 0.0172); regulation of cholesterol transport (GO:0032374; FDR = 0.0329) and peroxisome proliferator activated receptor signaling pathway (GO:0035357; FDR = 0.0239). Whereas the risk allele A of rs7928675 generates DNA binding sites for 30 TFs involved in: inflammatory responses: interleukin-9-mediated signaling pathway (GO:0038113; FDR = 0.00720), cytokine production (GO:0001816; FDR = 0.0479); cell signaling: cellular response to growth factor stimulus (GO:0071363; FDR = 0.0252), calcineurin-NFAT signaling cascade (GO:0033173; FDR = 0.0205), receptor signaling pathway via JAK-STAT (GO:0007259; FDR = 0.0065); cellular stress and hypoxia: response to hypoxia (GO:0001666; FDR = 0.0303), cellular response to stress (GO:0033554; FDR = 0.0387); apoptosis: regulation of apoptotic process (GO:0042981; FDR = 0.00556), regulation of neuron death (GO:0014041; FDR = 0.0373); vascular remodeling: cellular response to angiotensin (GO:1904385; FDR = 0.0452) (Table S17).

#### 3.2.4. Bioinformatic Analysis of the Associations of C11orf58 SNPs with IS-Related Phenotypes

According to the bioinformatic resources Cerebrovascular Disease Knowledge Portal (CDKP) and Cardiovascular Disease Knowledge Portal (CVDKP), which combine and analyze the results of genetic associations of the largest consortiums for the study of cardio- and cerebrovascular diseases, the IS-related *C11orf58* SNPs are associated with different types of stroke (lacunar stroke, TOAST small artery occlusion, large artery atherosclerosis major) and a number of stroke-related phenotypes, like blood pressure, total cholesterol, LDL cholesterol, serum ApoB, and atrial fibrillation (Table S19).

### 3.3. Gene-Gene and Gene-Environmental Interactions of C11orf58 and Other Genes, Encoding Hero Proteins

#### 3.3.1. Intergenic Interactions

Ten most significant models of intergenic interactions associated with the IS risk were established using the MB-MDR method: three two-locus models, four three-locus models, and three four-locus models (Pperm ≤ 0.001) (Table 6). It should be noted that the most significant models of G × G interactions associated with IS included polymorphic loci of two Hero genes: *C19orf53* and *C11orf58*.

**Figure 2:**
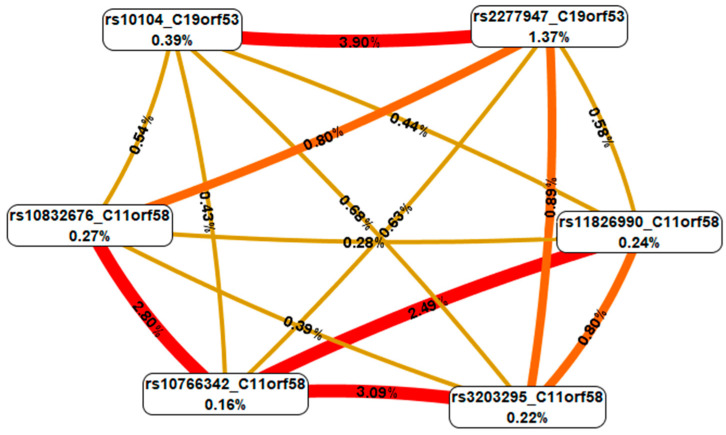
Graph reflecting the structure and strength of the most significant G × G interactions of Hero genes associated with IS. The color of the lines reflects the nature of the interaction: red and orange lines mean pronounced and moderate synergism, brown—additive effect of genes (independent effects); % reflects the strength and direction of the phenotypic effect of gene interaction (% entropy).

The subsequent analysis of these genetic variants using the MDR method (carried out for the three most significant interactions of high-order genes—four-locus), first revealed that the majority of the interactions between the genetic variants of Hero were characterized by pronounced and moderate synergism, with the exception of rs10104 *C19orf53*, which has independent effects (additive effect) with all SNPs analyzed by the MDR method, with the exception of the interactions with rs2277947 *C19orf53* (Figure 2).

Second, rs2277947 *C19orf53* had the most pronounced mono-effect (1.37% of trait entropy). Third, the effects of the intergenic interactions (0.28–3.90% entropy) exceeded or were comparable to the mono-effects of the SNPs (0.16–1.37% entropy). Fourth, the following combinations of the genotypes of Hero polymorphic variants had the most pronounced associations with IS: rs3203295 *C11orf58* A/C × rs10766342 *C11orf58* G/G × rs10104 *C19orf53* A/A × rs2277947 *C19orf53* G/G (beta = 0.543; *p* = 5.21 × 10^−8^), rs3203295 *C11orf58* A/A × rs10766342 *C11orf58* G/A × rs10104 *C19orf53* A/A × rs2277947 *C19orf53* G/G (beta = 0.588; *p* = 9.05 × 10^−6^), rs3203295 *C11orf58* A/A × rs10766342 *C11orf58* G/G × rs10104 *C19orf53* A/G × rs2277947 *C19orf53* G/G (beta = 0.492; *p* = 1.43 × 10^−6^), rs10832676 *C11orf58* A/G × rs10766342 *C11orf58* G/G × rs10104 *C19orf53* A/A × rs2277947 *C19orf53* G/G (beta = 0.616; *p* = 0.0001), rs10832676 *C11orf58* A/A × rs10766342 *C11orf58* G/A × rs10104 *C19orf53* A/A × rs2277947 *C19orf53* A/A (beta = 0.589; *p* = 0.0005), rs10832676 *C11orf58* A/A × rs10766342 *C11orf58* G/A × rs10104 *C19orf53* A/A × rs2277947 *C19orf53* G/G (beta = 0.596; *p* = 0.002), rs11826990 *C11orf58* T/G × rs10766342 *C11orf58* G/G × rs10104 *C19orf53* A/A × rs2277947 *C19orf53* G/G (beta = 0.544, *p* = 2.61 × 10^−8^), rs11826990 *C11orf58* T/T × rs10766342 *C11orf58* G/G × rs10104 *C19orf53* A/G × rs2277947 *C19orf53* G/G (beta = 0.487; *p* = 1.828 × 10^−6^), rs11826990 *C11orf58* T/T × rs10766342 *C11orf58* G/G × rs10104 *C19orf53* A/A × rs2277947 *C19orf53* G/G (beta = −0.100; *p* = 1.23 × 10^−5^) (Table S20).

#### 3.3.2. Gene-Environment Interactions

When analyzed by the MB-MDR method, nine of the most significant models of gene-smoking interactions were identified, including one two-factor, four three-factor, and four four-factor models (Pperm ≤ 0.01) (Table 7).

The best models of G × E interactions, in addition to smoking, included exclusively polymorphic variants of the Hero genes *C19orf53* and *C11orf58*, suggesting their most important role in the risk of developing IS in interaction with smoking. The most significant statistical indicators were distinguished by the following genotype-environment combinations: smoking × rs10734249 *C11orf58* A/A (beta = 0.218; *p* = 3.25 × 10^−14^), non-smoking × rs10104 *C19orf53* A/A × rs2277947 *C19orf53* G/G (beta = −0.15; *p* = 5.41 × 10^−11^), non-smoking × rs11024031 *C11orf58* C/T × rs10734249 *C11orf58* A/A (beta = −0.148; *p* = 4.49 × 10^−6^), non-smoking × rs3802963 *C11orf58* G/G × rs10734249 *C11orf58* A/A (beta = −0.201; *p* = 2.77 × 10^−15^), smoking × rs7928675 *C11orf58* A/A × rs10734249 *C11orf58* A/A (0.281; *p* = 2.36 × 10^−16^), non-smoking × rs11024031 *C11orf58* C/T × rs10734249 *C11orf58* A/A × rs6677 *C11orf58* T/G (beta = −0.175; *p* = 1.41 × 10^−7^), smoking × rs10734249 *C11orf58* A/A × rs10104 *C19orf53* A/A × rs2277947 *C19orf53* G/G (beta = 0.226; *p* = 8.59 × 10^−10^), non-smoking × rs11024032 *C11orf58* C/C× rs10766342 *C11orf58* G/G × rs10734249 *C11orf58* A/A (beta = −0.211; *p* = 5.64 × 10^−13^), non-smoking × rs10734249 *C11orf58* A/A × rs2277947 *C19orf53* G/G × rs11666524 *C19orf53* G/G (beta = −0.142; *p* = 4.15 × 10^−8^) (Table S21).

**Figure 3:**
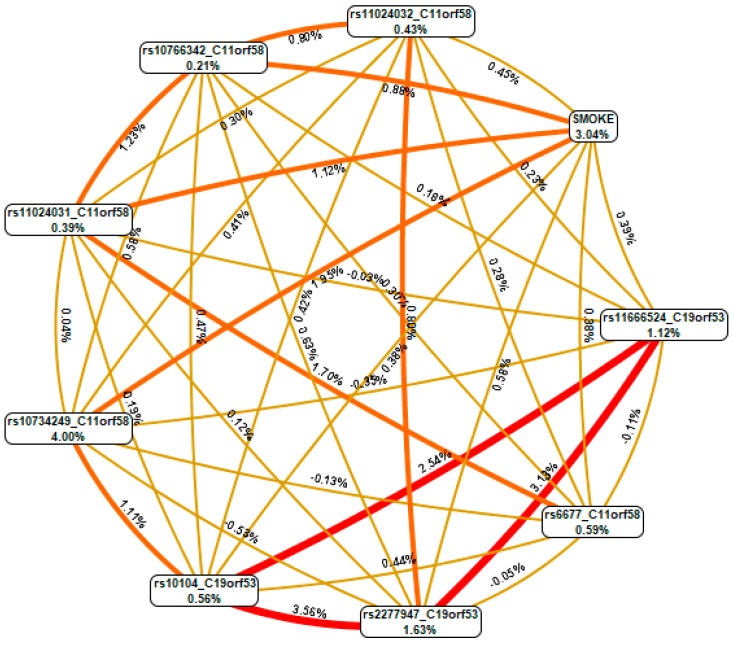
Graph of the most significant G × E interactions associated with the development of IS. The color of the line reflects the nature of the interaction: red and orange—pronounced and moderate synergism, brown—independent effect of individual loci; % reflects the strength and direction of the phenotypic effect of gene interaction (% entropy).

Using the MDR method, the interactions between smoking and eight SNPs included in the best high-order gene-environment interaction models were analyzed and visualized in the form of a graph (Figure 3).

The data presented in the graph indicate that, first, the contribution of smoking (mono-effect) to the entropy of IS (3.04%) was comparable to the mono-effects of the SNPs included in the best G × E models (0.21–4.00%). Second, smoking was characterized by moderate synergism/additive effects in interaction with polymorphic variants of the Hero genes. Third, the contribution of the gene-environment interactions to the entropy of IS (0.38–1.12%) is comparable to the mono-effects of SNPs/smoking (0.21–4.00%). Fourth, the most pronounced mono-effect, besides smoking, was characterized by rs10734249 *C11orf58* (4% contribution to the entropy of the IS). Fifth, polymorphic loci *C11orf58* were characterized by predominantly independent (additive) effects in interaction with loci *C19orf53*, which were included in the best models of G × E interactions.

The summary of the effects of the entropy of Hero SNPs on the contribution to IS risk is provided in Table 8.

## 4. Discussion

In this study, we are the first to report that polymorphisms in the gene encoding Chromosome 11 Open Reading Frame 58 (*C11orf58*) are associated with the risk of IS. Specifically, SNPs rs10766342 (risk allele A), rs11024032 (risk allele T), rs11826990 (risk allele G), rs3203295 (risk allele C), rs10832676 (risk allele G), and rs4757429 (risk allele T) were found to be associated with an elevated risk of IS in the total study population. However, we observed that cigarette smoking and the consumption of fresh vegetables and fruit acted as modifiers of the associations of these SNPs with IS risk. These genetic variants were associated with increased IS risk in smokers and individuals with low fruit and vegetable consumption. Additionally, rs11024030 (risk allele C) was linked to an increased risk of IS exclusively in smokers and in patients with low fruit/vegetable intake. Meanwhile, rs7928675 and rs7951676 were associated with a reduced risk of IS only among smokers. In conducting linkage disequilibrium analysis in the group of smokers between IS-associated SNPs, we have revealed that the protective effects of rs7928675 and rs7951676 were connected to being in negative linkage disequilibrium with the risk SNPs. We found that loci of two Hero genes, *C11orf58* and *C19orf53,* featured the most significant gene–gene and gene-smoking interactions associated with IS risk.

Furthermore, the *C11orf58* haplotypes of tag SNPs were associated with IS. The haplotype rs3802963G-rs7951676G-rs11024032T-rs6677T *C11orf58* was linked to an increased risk of IS in the entire group and in smokers. The haplotype rs3802963G-rs7951676T-rs11024032C-rs6677T *C11orf58* was associated with a reduced risk of IS in smokers, while the haplotype rs3802963G-rs7951676G-rs11024032C-rs6677T *C11orf58* was linked to an increased risk of IS in nonsmokers. In patients with low fruit and vegetable intake, haplotypes rs3802963G-rs7951676G-rs11024032C-rs6677T *C11orf58* and rs3802963C-rs7951676T-rs11024032C-rs6677T *C11orf58* were identified as associated with an increased risk of IS.

Since the chaperone properties of *C11orf58* encoding Hero20 were discovered recently (2020) and functional studies are virtually absent, as well as association studies of variations in the *C11orf58* gene in terms of their relationship with multifactorial human diseases, we resorted to bioinformatics analysis to interpret the established relationships. According to data from the eQTLGene browser, IS-associated *C11orf58* SNPs reduce expression levels of *C11orf58* and *PIK3C2A* genes within the blood. Interestingly, PIK3C2A was recognized for its diverse biological functions encompassing glucose transport, angiogenesis, Akt activation, endosomal trafficking, phagosome maturation, mitotic spindle organization, exocytosis, and autophagy [68,69,70,71,72]. Specifically, Yoshioka et al. have reported that the effects of *PI3KC2A* knockdown led to defective endothelial cell migration, proliferation, tube formation, and blood–brain barrier integrity—critical processes in IS pathogenesis [73,74]. Endothelial PI3KC2A deficiency in vivo suppressed postischemic angiogenesis and diminished vascular barrier function [72]. Moreover, results of Tan et al. revealed a significant decrease in the expression of the *PIK3C2A* gene in the peripheral blood of patients with acute myocardial infarction [75]—a pathologically closely related disease to IS. In addition to PIK3C2A’s significant role in cellular function through vesicular transport [72], this protein is also implicated in the PI3K/AKT/mTOR signaling pathway, which regulates cell survival, differentiation, and development [76]. This interplay between SNPs of *C11orf58* and the consequential decrease in *PIK3C2A* expression highlights a potential regulatory mechanism with implications for both IS and related cardiovascular conditions.

Additional support for the link between risk alleles and underexpression of *C11orf58* was evident in the discovery that the risk alleles for all IS-related SNPs were associated with mQTLs. This correlation results in increased methylation levels at CpG sites, specifically cg05512310, cg05405872, cg20474675, and cg01047635 within the prefrontal cortex.

It is noteworthy that the risk alleles of SNPs associated with IS in our study also demonstrated an increased risk of lacunar stroke (rs11024030, rs3203295, rs10832676, rs4757429), small artery occlusion (rs11024032), and elevated systolic and diastolic blood pressure (rs10766342, rs11024030, rs11826990, rs3203295, rs10832676, rs4757429, rs7928675, rs7951676), as indicated by data from the Cerebrovascular Disease Knowledge Portal. Additionally, information gathered through the bioinformatic resource, the Cardiovascular Disease Knowledge Portal, revealed that the risk alleles of IS-associated SNPs were associated with reduced levels of LDL cholesterol (rs10766342, rs11024032, rs11826990, rs3203295) and total cholesterol (rs10766342, rs11024030, rs11024032, rs11826990, rs3203295, rs10832676, rs4757429), suggesting the potential involvement of polymorphic variants in *C11orf58* in the development of atherosclerosis and thrombosis, the major causes of IS.

To identify the impact of IS-associated SNPs of *C11orf58* on histone modification regions, we employed the bioinformatic tool HaploReg v4.2. The analysis revealed that the IS-associated SNPs of *C11orf58* (rs10766342, rs11826990, rs3203295, rs7928675) exhibit a high level of regulatory potential, exerting profound effects on histone modifications within brain and blood tissues. Notably, they are located in the active promoter/enhancer regions both in blood and brain tissues.

**Figure 4:**
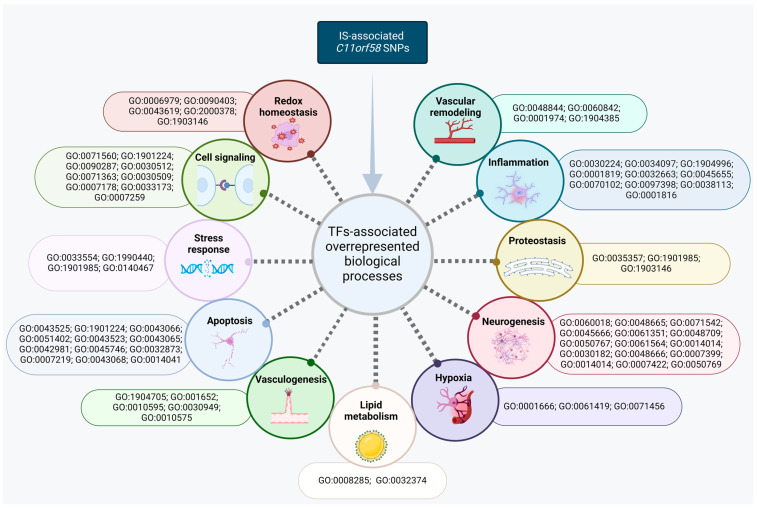
TF-associated overrepresented biological processes of *C11orf58* SNPs.

Given the complex pathophysiology of IS, the accompanying injury and signaling mechanisms should be analyzed in connection with *C11orf58* polymorphic variants, specifically through the modulation of transcriptional activities via interaction with TFs, leading to distinct functional outcomes such as loss or gain of function. In Figure 4, we outlined the main overrepresented biological processes associated with TFs binding to *C11orf58* SNPs.

First, SNPs (rs10766342, rs11024030, rs11826990, rs3203295, rs10832676, rs4757429, and rs7928675) exhibited a significant impact on neuronal cell development and differentiation and on vasculogenesis and vascular remodeling (rs10766342, rs11024032, rs11826990, rs10832676, rs4757429, and rs7928675). Second, the risk allele A of rs10766342, the risk allele T of rs11024032, the risk allele G of rs11826990, and the risk allele A of rs7928675 create binding sites for TFs that regulate inflammation and immune response—a critical cellular response in IS. This inflammatory reaction, though implicated in nerve tissue damage and cell death, plays an essential role in tissue remodeling and repair during nerve cell recovery [77,78]. Additionally, inflammatory mediators contribute to the initiation and progression of atherosclerosis [15,79]. Furthermore, the protective allele C of rs7928675 generates binding sites for TFs regulating cholesterol transport, a crucial element in maintaining overall cholesterol homeostasis to prevent atherosclerosis—a major contributor to IS [80,81]. Third, effect alleles of rs11024032, rs10832676, rs4757429, and rs7928675 create binding sites for TFs that participate in hypoxia and oxidative stress responses–the most important mechanisms for cardiovascular diseases in general [82,83,84] and ischemic stroke in particular [85,86]. Excessive reactive oxygen species (ROS), triggered by hypoxia, are the primary instigators of cellular damage and cell death in ischemic injury [87,88]. Additionally, the risk allele G of rs10832676 generated binding sites for TFs regulating protein acetylation—an essential process within proteostasis. Dysregulation in proteostasis can lead to protein aggregation and disruptions in neuron communication, further contributing to neuronal degeneration [89,90,91].

Further examination of TFs-associated overrepresented biological processes revealed the participation of *C11orf58* in crucial pathways responsible for cell survival in ischemia.

Effect alleles of rs11024030, rs11826990, rs3203295, and rs7928675 generated binding sites for TFs involved in apoptosis regulation either directly or through specific pathways, including the Notch signaling pathway (rs10832676, rs4757429), the negative regulation of stress-activated MAPK cascade (rs4757429), or the NIK/NF-kappaB signaling (rs11024032). In the context of IS, Notch activation poses a threat to neurons by modulating pathways that increase their vulnerability to apoptosis, pro-inflammatory leukocyte infiltration, and microglial activation [92]. Although reduced Notch signaling is necessary and sufficient to trigger neurogenesis by parenchymal astrocytes [93], the neurogenic response is more widespread in the stroke-afflicted striatum when Notch signaling is blocked [92,94,95]. MAPK cascade activation can be pro- and/or anti-apoptotic [96]. Additionally, the MAPK signaling pathway may inhibit the activation and hyperproliferation of glial cells by regulating the cell cycle and reducing harmful secreted factors that damage neurons [97,98,99,100]. Conversely, this pathway also mitigates glial proliferation and swelling induced by local microcirculatory disorders, thereby creating a favorable environment for neuronal survival rather than apoptosis [101]. The implications of the MAPK signaling pathway in the regulation of cytokine expression and cell apoptosis after stroke have also been identified [102]. NF-κB-inducing kinase (NIK) leads to an activation of NF-κB in neurons, contributing to ischemia-induced neuronal injury [103,104]. Despite NF-κB well-known role as an antiapoptotic factor, in cerebral ischemia, it paradoxically contributes to neuronal cell death, particularly in cases of severe ischemia leading to irreversible brain damage, as discussed by Ridder and Schwaninger in 2009 [105].

The risk allele G of rs10832676 creates binging sites for TFs associated with the peroxisome proliferator activated receptor (PPAR) signaling pathway. The PPAR family controls gene expression in downstream networks involved in lipogenesis, lipid metabolism, inflammation, and metabolic homeostasis [106,107]. PPAR-γ, specifically, confers anti-atherosclerotic effects in the vascular system [108,109,110] and neuroprotective effects in the central nervous system by minimizing the harmful effects of excitotoxicity and increasing the survival rates of neuron cells [111,112,113,114]. The risk allele A of rs7928675 generates binding sites for TFs participating in the calcineurin-NFAT signaling cascade. In ischemia, characterized by excitotoxicity and Ca^2+^ overload [115], one well-established pathway through which cells translate changes in calcium levels into differential gene expression is the action of the phosphatase calcineurin on NFAT transcription factors [116]. NFATs can activate or suppress numerous gene expression programs linked to immune/inflammatory signaling, Ca^2+^ regulation, and cell survival [117]. Notably, the blockage of the astrocytic calcineurin/NFAT signaling has been shown to contribute to normalizing hippocampal synaptic function and plasticity in a rat model of traumatic brain injury [118]. Lastly, the risk allele A of rs7928675 creates binding sites for TFs involved in the JAK-STAT signaling pathway. Activated under ischemic conditions, this pathway remains highly expressed in neurons and nerve cells around the infarction, responding to various pathogenic factors, including apoptosis, inflammatory response, vascular remodeling, oxidative stress, and autophagy [119,120].

By considering the pathways through which TFs bind to the alleles of *C11orf58*, we can speculate a broader and more intricate range of roles for *C11orf58* within neuron cells, given its association with fundamental cellular pathways—the primary defenses in ischemia survival. Consequently, our hypothesis extends beyond *C11orf58* merely elevating the risk of IS, as indicated by our genetic analysis, to encompass its involvement in intricate mechanisms that govern the survival of neurons, glia, and endothelial cells. This involvement manifests through the gain and loss of binding sites for TFs.

## 5. Conclusions

Our study identified significant associations between SNPs of the Hero gene *C11orf58* and IS risk. Through bioinformatic analysis, we uncovered the molecular mechanisms by which these risk SNPs contribute to IS. Additionally, gene–gene and gene–environmental interactions involving five Hero genes were examined, revealing that *C19orf53* and *C11orf58* SNPs form the most impactful models for determining IS risk.

Future prospects for this study could include analyzing *C11orf58* gene expression profiles and DNA methylation levels at different stages of stroke, such as the acute and recovery phases, to gain a deeper understanding of *C11orf58*’s role in stroke. Additionally, investigating *C11orf58’s* role within gene networks and pathways may provide valuable insights into new molecular mechanisms underlying the pathogenesis of cerebrovascular conditions. By integrating genetic and environmental factors, our findings contribute to advancing personalized medicine for ischemic stroke, opening new possibilities for effective prevention and management strategies.

## 6. Study Limitations

This study has several limitations. First, we did not measure *C11orf58* expression levels, limiting our ability to determine the direct effect of the studied SNPs on *C11orf58* mRNA expression. Second, our study focused exclusively on a Russian cohort, and the absence of a patient sample from other populations prevented us from performing a replication study to validate our findings across diverse ethnic groups. While we referenced data from the Cerebrovascular Disease Knowledge Portal for cross-population associations, further replication in additional cohorts is warranted. Third, we lacked data on several IS risk factors, like fresh vegetables and fruit intake and physical activity, in the control group, so we could not adjust our calculations for these variables. Fourth, our analysis represents a cross-sectional snapshot of *C11orf58* polymorphisms in association with IS risk and does not assess how these genetic variants might impact IS progression or long-term outcomes.

## Figures and Tables

**Table 1:** Baseline and clinical characteristics of the studied groups.

Baseline and Clinical Characteristics		IS Patients (n = 917)	Controls (n = 1287)	*p*-Value
Age, Me [Q1; Q3]		62 [55; 70]	58 [53; 66]	**<0.001**
Gender	Males, N (%)	507 (55.3%)	594 (46.1%)	**<0.001**
	Females, N (%)	410 (44.7%)	693 (53.9%)	
Smoking	Yes, N (%)	444 (48.4%)	386 (30%)	**<0.001**
	No, N (%)	473 (51.6%)	901 (70%)	
Low physical activity	Yes, N (%)	349 (38.1%)	ND	
	No, N (%)	530 (57.8%)		
	ND, N (%)	38 (4.1%)		
Low fruit/vegetable consumption	Yes, N (%)	469 (51.2%)	ND	
	No, N (%)	410 (44.7%)		
	ND, N (%)	38 (4.1%)		
Coronary artery disease	Yes, N (%)	115 (12.6%)	-	
	No, N (%)	765 (83.5%)	-	
	ND, N (%)	37 (3.9%)		
Body mass index, Me [Q1; Q3]		23 [22; 26] (n = 585)	-	
Family history of cerebrovascular diseases	Yes, N (%)	309 (33.7%)	0 (0%)	
	No, N (%)	567 (61.8%)	0 (0%)	
	ND, N (%)	41 (4.5%)	1287 (100%)	
Age at onset of stroke, Me [Q1; Q3]		61 [54; 70] (n = 896)	-	
Number of strokes including event in question	1, N (%)	796 (88.8%)	-	
	2, N (%)	87 (9.7%)	-	
	3, N (%)	13 (1.5%)	-	
Stroke location	Right/left middle cerebral arteries, N (%)	745 (81.2%)	-	
	Vertebrobasilar arteries, N (%)	149 (16.3%)	-	
	ND, N (%)	23 (2.5%)		
Stroke size, mm^2^, Me [Q1; Q3]		108 [30; 471] (n = 876)	-	
Total cholesterol, mmol/L, Me [Q1; Q3]		5.2 [4.4; 5.9] (n = 601)	ND	
Triglycerides, mmol/L, Me [Q1; Q3]		1.3 [1.1; 1.8] (n = 594)	ND	
Prothrombin time, seconds, Me [Q1; Q3]		10.79 [10.14; 11.70] (n = 873)	ND	
International normalized ratio, Me [Q1; Q3]		1 [0.94; 1.09] (n = 590)	ND	
Activated partial thromboplastin time, seconds, Me [Q1; Q3]		32.7 [29; 37] (n = 593)	ND	

Statistically significant differences between groups are indicated in bold. Me—median, Q1—first quartile, Q3—third quartile, ND—no data.

**Table 2:** Summary of the analysis of the associations between *C11orf58* SNPs and ischemic stroke risk.

Genetic Variant	Effect Allele	Other Allele	OR [95% CI] ^1^	*p* ^2^	OR [95% CI] ^1^	*p* ^2^	OR [95% CI] ^1^	*p* ^2^
			Entire Group		Smokers		Low Fruit/Vegetable Intake [f+]	
rs10766342 *C11orf58*	A	G	1.21 [1.03–1.43]	**0.02**	1.42 [1.09–1.86]	**0.009**	1.26 [1.04–1.53]	**0.02** **(0.04)**
rs7928675 *C11orf58*	C	A	0.89 [0.75–1.07]	0.21	0.73 [0.56–0.96]	**0.025**	0.93 [0.75–1.15]	0.49 (0.98)
rs11024030 *C11orf58*	C	T	1.16 [0.99–1.37]	0.064	1.33 [1.02–1.72]	**0.03**	1.26 [1.05–1.52]	**0.016** **(0.032)**
rs11024032* *C11orf58*	T	C	1.22 [1.04–1.44]	**0.01**	1.39 [1.07–1.81]	**0.01**	1.26 [1.04–1.52]	**0.02** **(0.04)**
rs7951676* *C11orf58*	T	G	0.88 [0.74–1.05]	0.17	0.75 [0.57–0.99]	**0.04**	0.91 [0.74–1.12]	0.38 (0.76)
rs11826990 *C11orf58*	G	T	1.25 [1.06–1.47]	**0.007**	1.48 [1.13–1.93]	**0.004**	1.26 [1.05–1.53]	**0.017** **(0.034)**
rs3203295 *C11orf58*	C	A	1.22 [1.04–1.44]	**0.016**	1.40 [1.07–1.83]	**0.01**	1.26 [1.04–1.53]	**0.02** **(0.04)**
rs10832676 *C11orf58*	G	A	1.26 [1.07–1.48]	**0.006**	1.53 [1.17–2.00]	**0.002**	1.30 [1.07–1.57]	**0.007** **(0.014)**
rs4757429 *C11orf58*	T	C	1.21 [1.03–1.42]	**0.02**	1.32 [1.01–1.71]	**0.04**	1.25 [1.03–1.51]	**0.02** **(0.04)**

All calculations were performed relative to the minor alleles (effect allele) with adjustment for sex, age, and smoking; ^1^—odds ratio and 95% confidence interval; ^2^—*p*-value; tag SNPs are marked with an asterisk; statistically significant differences are marked in bold.

**Table 3:** Association of *C11orf58* SNPs with cis-eQTL-Mediated Expression Profiles of Genes in Whole Blood.

SNP	Allele	Gene Expressed	Z-Score	*p*-Value	FDR
rs10766342 *C11orf58* (G/**A**)	A	*C11orf58*	↓(−12.28)	1.19 × 10^−34^	0
		*PIK3C2A*	↓(−4.37)	1.26 × 10^−5^	0.038
rs11024030 *C11orf58* (T/**C**)	C	*C11orf58*	↓(−12.22)	2.36 × 10^−34^	0
		*PIK3C2A*	↓(−4.28)	1.85 × 10^−5^	0.046
rs11024032 *C11orf58* (C/**T**)	T	*C11orf58*	↓(−12.24)	1.85 × 10^−34^	0
		*PIK3C2A*	↓(−4.33)	1.48 × 10^−5^	0.038
rs11826990 *C11orf58* (T/**G**)	G	*C11orf58*	↓(−12.20)	3.22 × 10^−34^	0
rs3203295 *C11orf58* (A/**C**)	C	*C11orf58*	↓(−12.20)	3.00 × 10^−34^	0
		*PIK3C2A*	↓(−4.32)	1.55 × 10^−5^	0.039
rs10832676 *C11orf58* (A/**G**)	G	*C11orf58*	↓(−12.24)	1.80 × 10^−34^	0
		*PIK3C2A*	↓(−4.28)	1.85 × 10^−5^	0.046
rs4757429 *C11orf58* (C/**T**)	T	*C11orf58*	↓(−12.22)	2.42 × 10^−34^	0
		*PIK3C2A*	↓(−4.26)	1.98 × 10^−5^	0.049
rs7928675 *C11orf58* (A/**C**)	C	*C11orf58*	↓(−8.23)	1.94 × 10^−16^	0
rs7951676 *C11orf58* (G/**T**)	T	*C11orf58*	↓(−8.21)	2.17 × 10^−16^	0

Note: FDR—false discovery rate; Effect alleles are marked in bold.

**Table 4:** Established associations of the studied *C11orf58* SNPs with the cis-mQTL-mediated effect on the level of methylation of CpG-sites.

Trait	Effect Allele	Tissue	Effect Size (Beta)	FDR
rs10766342 *C11orf58*				
cg05512310 (chr11:16804714) *C11orf58*	A	Brain-Prefrontal Cortex	0.01	1.3 × 10^−3^
cg05405872 (chr11:16804957) *C11orf58*	A	Brain-Prefrontal Cortex	0.01	1.7 × 10^−3^
cg20474675 (chr11:16804716) *C11orf58*	A	Brain-Prefrontal Cortex	0.01	2.7 × 10^−3^
rs11024030 *C11orf58*				
cg05405872 (chr11:16804957) *C11orf58*	C	Brain-Prefrontal Cortex	0.02	8.6 × 10^−4^
rs11024032 *C11orf58*				
cg05405872 (chr11:16804957) *C11orf58*	T	Brain-Prefrontal Cortex	0.01	5.0 × 10^−3^
rs11826990 *C11orf58*				
cg05512310 (chr11:16804714) *C11orf58*	G	Brain-Prefrontal Cortex	0.01	1.3 × 10^−3^
cg05405872 (chr11:16804957) *C11orf58*	G	Brain-Prefrontal Cortex	0.01	1.7 × 10^−3^
cg20474675 (chr11:16804716) *C11orf58*	G	Brain-Prefrontal Cortex	0.01	2.7 × 10^−3^
rs3203295 *C11orf58*				
cg05512310 (chr11:16804714) *C11orf58*	C	Brain-Prefrontal Cortex	0.01	0.002
cg20474675 (chr11:16804716) *C11orf58*	C	Brain-Prefrontal Cortex	0.01	0.004
cg05405872 (chr11:16804957) *C11orf58*	C	Brain-Prefrontal Cortex	0.01	0.006
cg01047635 (chr11:16804930) *C11orf58*	C	Brain-Prefrontal Cortex	0.01	0.008
rs10832676 *C11orf58*				
cg05405872 (chr11:16804957) *C11orf58*	G	Brain-Prefrontal Cortex	0.02	8.6 × 10^−4^
rs4757429 *C11orf58*				
cg05405872 (chr11:16804957) *C11orf58*	T	Brain-Prefrontal Cortex	0.02	8.6 × 10^−4^

cg—CpG sites, FDR—false discovery rate. Only data with known effect allele is presented in the table.

**Table 5:** The impact of *C11orf58* SNPs on histone tags in various tissues.

SNP (Ref/Alt Allele)	Marks	Brain							Blood
		(1)	(2)	(3)	(4)	(5)	(6)	(7)	(8)
rs10766342 *C11orf58* (G/**A**)	H3K4me1	Enh	Enh	Enh	Enh	-	-	-	Enh
	H3K4me3	Pro	Pro	Pro	Pro	Pro	Pro	Pro	Pro
	H3K27ac	Enh	Enh	Enh	Enh	Enh	Enh	Enh	Enh
	H3K9ac	-	Pro	Pro	Pro	Pro	Pro	Pro	Pro
	DNase	-	-	-	-	-	-	-	DNase
rs11024030 *C11orf58* (T/**C**)	H3K4me1	Enh	-	-	Enh	-	-	Enh	Enh
	H3K27ac	-	-	-	-	-	-	-	Enh
rs11024032 *C11orf58* (C/**T**)	H3K4me1	-	Enh	-	Enh	-	Enh	-	Enh
	H3K27ac	-	-	-	-	-	-	Enh	Enh
	H3K9ac	-	-	Pro	-	-	-	-	-
rs11826990 *C11orf58* (T/**G**)	H3K4me1	Enh	-	-	-	-	-	-	Enh
	H3K4me3	Pro	Pro	Pro	Pro	Pro	Pro	Pro	Pro
	H3K27ac	Enh	Enh	Enh	Enh	Enh	Enh	Enh	Enh
	H3K9ac	-	Pro	Pro	Pro	Pro	Pro	Pro	Pro
	DNase	-	-	-	-	-	-	-	DNase
rs3203295 *C11orf58* (A/**C**)	H3K4me1	Enh	Enh	Enh	Enh	Enh	Enh	Enh	Enh
	H3K4me3	Pro	Pro	Pro	Pro	Pro	Pro	Pro	Pro
	H3K27ac	Enh	Enh	Enh	Enh	Enh	Enh	Enh	Enh
	H3K9ac		Pro	Pro	Pro	Pro	Pro	Pro	Pro
	DNase	-	-	-	-	-	-	-	DNase
rs10832676 *C11orf58* (A/**G**)	H3K4me1	-	-	Enh	Enh	Enh	Enh	Enh	Enh
	H3K4me3	-	-	-	-	-	-	-	Pro
	H3K27ac	-	-	-	-	-	-	-	Enh
rs4757429 *C11orf58* (C/**T**)	H3K4me1	Enh	Enh	Enh	Enh	Enh	Enh	Enh	Enh
	H3K4me3	Pro	Pro	Pro	Pro	Pro	Pro	Pro	Pro
	H3K27ac	-	-	-	-	Enh	-	Enh	Enh
	H3K9ac	-	-	-	-	-	-	-	Pro
rs7928675 *C11orf58* (A/**C**)	H3K4me1	Enh	Enh	Enh	Enh	Enh	Enh	Enh	Enh
	H3K4me3	Pro	Pro	Pro	Pro	Pro	Pro	Pro	Pro
	H3K27ac	Enh	-	Enh	Enh	Enh	Enh	Enh	Enh
	H3K9ac	-	Pro	Pro	Pro	Pro	Pro	Pro	Pro
rs7951676 *C11orf58* (G/**T**)	H3K4me1	-	-	Enh	Enh	Enh	Enh	Enh	Enh
	H3K4me3	-	-	-	-	-	-	-	Pro
	H3K27ac	-	-	-	-	-	-	-	Enh
	H3K9ac	-	-	-	-	-	-	-	Pro

H3K4me1—mono-methylation at the 4th lysine residue of the histone H3 protein; H3K4me3—tri-methylation at the 4th lysine residue of the histone H3 protein; H3K9ac—the acetylation at the 9th lysine residues of the histone H3 protein; H3K27ac—acetylation of the lysine residues at N-terminal position 27 of the histone H3 protein; alternative alleles are marked in bold. Enh—histone modification in the enhancer region; Pro—histone modification at the promoter region. 1—Brain Hippocampus Middle; 2—Brain Substantia Nigra; 3—Brain Anterior Caudate; 4—Brain Cingulate Gyrus; 5—Brain Inferior Temporal Lobe; 6—Brain Angular Gyrus; 7—Brain Dorsolateral Prefrontal Cortex 8—Cells from peripheral blood (any).

**Table 6:** Gene–gene interactions of polymorphic variants of the Hero genes associated with susceptibility to IS (MB-MDR modeling).

Gene–Gene Interaction Models	NH	Beta H	WH	NL	Beta L	WL	Wmax	P_perm_
The best two-locus models of intergenic interactions (for models with Pmin. < 1 × 10^−17^, 1000 permutations)								
rs10104 *C19orf53*×rs2277947 *C19orf53*	6	0.4708	104.46	2	−0.29994	70.758	104.46	<0.001
rs10104 *C19orf53*×rs11666524 *C19orf53*	6	0.4868	80.29	1	−0.06732	9.155	80.29	<0.001
rs3203295 *C11orf58*×rs10766342 *C11orf58*	2	0.5602	75.06	1	−0.09136	16.641	75.06	<0.001
The best three-locus models of intergenic interactions (for models with Pmin. < 5 × 10^−24^, 1000 permutations)								
rs10104 *C19orf53*×rs2277947 *C19orf53*×rs11666524 *C19orf53*	11	0.5044	133.3	2	−0.33361	98.43	133.3	<0.001
rs10104 *C19orf53*×rs346157 *C19orf53*×rs2277947 *C19orf53*	10	0.5214	109.3	1	−0.08513	11.74	109.3	<0.001
rs8107914 *C19orf53*×rs10104 *C19orf53*×rs2277947 *C19orf53*	9	0.5135	107.0	2	−0.07575	10.51	107.0	<0.001
rs10104 *C19orf53*×rs346158 *C19orf53*×rs2277947 *C19orf53*	9	0.5025	106.6	2	−0.09005	15.31	106.6	<0.001
The best four-locus models of gene–gene interactions (for models with Pmin. < 1 × 10^−34^, 1000 permutations)								
rs3203295 *C11orf58*×rs10766342 *C11orf58*×rs10104 *C19orf53*×rs2277947 *C19orf53*	13	0.5459	171.7	3	−0.1991	69.61	171.7	<0.001
rs10832676 *C11orf58*×rs10766342 *C11orf58*×rs10104 *C19orf53*×rs2277947 *C19orf53*	12	0.5437	164.5	3	−0.2036	73.22	164.5	<0.001
rs11826990 *C11orf58*×rs10766342 *C11orf58*×rs10104 *C19orf53*×rs2277947 *C19orf53*	13	0.5466	162.4	3	−0.1988	69.17	162.4	<0.001

Note: NH is the number of interacting high-risk genotypes, beta H—regression coefficient for high-risk interactions identified at the 2nd stage of analysis, WH—Wald statistics for high-risk interactions, NL—number of interacting low-risk genotypes, beta L—regression coefficient for low-risk interactions identified at the 2nd stage of analysis, WL—Wald statistics for low-risk interactions, Pperm—permutational significance levels for models (all models are adjusted for gender, age, and smoking).

**Table 7:** Gene-environment interactions of polymorphic variants of Hero genes associated with susceptibility to IS (MB-MDR modeling).

Models of G × E Interactions	NH	Beta H	WH	NL	Beta L	WL	Wmax	Pperm
The best two-factor models of G × E interactions (for models with Pmin < 1 × 10^−24^, 1000 permutations)								
SMOKE×rs10734249 *C11orf58*	3	0.2605	110.26	3	−0.26052	110.264	110.26	<0.001
Best three-factor models of G × E interactions (for models with Pmin, < 1 × 10^−25^, 1000 permutations)								
SMOKE×rs10104 *C19orf53*×rs2277947 *C19orf53*	12	0.2577	118.6	2	−0.2335	116.75	118.6	<0.001
SMOKE×rs11024031 *C11orf58*×rs10734249 *C11orf58*	6	0.2729	118.0	5	−0.2406	95.42	118.0	<0.001
SMOKE×rs3802963 *C11orf58*×rs10734249 *C11orf58*	4	0.2636	110.6	3	−0.2634	114.35	114.4	<0.001
SMOKE×rs7928675 *C11orf58*×rs10734249 *C11orf58*	4	0.2851	114.2	4	−0.2467	100.95	114.2	<0.001
Best four-factor models of G × E interactions (for models with Pmin. < 2 × 10^−31^, 1000 permutations)								
SMOKE×rs11024031 *C11orf58*×rs10734249 *C11orf58*×rs6677 *C11orf58*	13	0.2872	131.9	6	−0.2998	147.33	147.3	<0.001
SMOKE×rs10734249 *C11orf58*×rs10104 *C19orf53*×rs2277947 *C19orf53*	13	0.3052	120.4	5	−0.2952	144.63	144.6	<0.001
SMOKE×rs11024032 *C11orf58*×rs10766342 *C11orf58*×rs10734249 *C11orf58*	12	0.2966	144.4	4	−0.2715	124.24	144.4	<0.001
SMOKE×rs10734249 *C11orf58*×rs2277947 *C19orf53*×rs11666524 *C19orf53*	14	0.3001	127.0	5	−0.2932	143.19	143.2	<0.001

Note: NH is the number of interacting high-risk genotypes, beta H—regression coefficient for high-risk interactions identified at the 2nd stage of analysis, WH—Wald statistics for high-risk interactions, NL—number of interacting low-risk genotypes, beta L—regression coefficient for low-risk interactions identified at the 2nd stage of analysis, WL—Wald statistics for low-risk interactions, Pperm—permutational significance levels for models (all models are adjusted for gender, age, and smoking).

**Table 8:** Summarized results of the analysis of G × G and G × E interactions using the MB-MDR and MDR methods (analysis of mono-effects of SNPs, G × G, and G × E interactions relative to the contribution to the entropy of IS).

SNP	G × G Interactions		G × E Interactions	
	Mono-Effect	GG-Effect	Mono-Effect	GE-Effect
rs10104 *C19orf53*	0.39%	5.99%	0.56%	8.67%
rs2277947 *C19orf53*	1.37%	6.8%	1.63%	9.47%
rs11826990 *C11orf58*	0.24%	4.59%	-	-
rs3203295 *C11orf58*	0.22%	5.85%	-	-
rs10766342 *C11orf58*	0.16%	9.44%	0.21%	5.07%
rs10832676 *C11orf58*	0.27%	4.81%	-	-
rs11666524 *C19orf53*			1.12%	7.01%
rs10734249 *C11orf58*	-	-	4.00%	5.46%
rs11024031 *C11orf58*	-	-	0.39%	5.15%
rs11024032 *C11orf58*	-	-	0.43%	3.71%
rs6677 *C11orf58*	-	-	0.59%	2.86%

Note: data presented for high-order (four locus and four factor models) gene–gene and gene–environmental interactions.

## Data Availability

The data presented in this study are available upon request from corresponding author.

## Supplementary Materials

The following supporting information can be downloaded at: https://www.mdpi.com/article/10.3390/biomedicines12112603/s1, Table S1: Characterization of *C11orf58* SNPs (NCBI data https://www.ncbi.nlm.nih.gov/); Table S2: Transcripts and proteins of Hero genes (NCBI and UniProt data); Table S3: Primers and probes designed for the study; Table S4: Used bioinformatic tools for bioinformatics analysis of the functional effects of *C11orf58* gene and *C11orf58* SNPs; Table S5: Distribution of *C11orf58* genotypes in ischemic stroke patients/healthy controls and their correspondence to the Hardy–Weinberg equilibrium; Table S6: Results of the analysis of the associations between *C11orf58* SNPs and ischemic stroke risk; Table S7: Subgroup’s analysis of the associations between *C11orf58* SNPs and IS risk depending on smoking status, fruit and vegetable intake; Table S8: Haplotype frequencies of the tag SNPs *C19orf58* gene and their associations with ischemic stroke risk; Table S9: Linkage disequilibrium indicators between IS-associated SNPs *C19orf58* in smokers; Table S10: Analysis of the effect of rs10766342 *C11orf58* on the binding of DNA to transcription factors; Table S11: Analysis of the effect of rs11024030 *C11orf58* on the binding of DNA to transcription factors; Table S12: Analysis of the effect of rs11024032 *C11orf58* on the binding of DNA to transcription factors; Table S13: Analysis of the effect of rs11826990 *C11orf58* on the binding of DNA to transcription factors; Table S14: Analysis of the effect of rs3203295 *C11orf58* on the binding of DNA to transcription factors; Table S15: Analysis of the effect of rs10832676 *C11orf58* on the binding of DNA to transcription factors; Table S16: Analysis of the effect of rs4757429 *C11orf58* on the binding of DNA to transcription factors; Table S17: Analysis of the effect of rs7928675 *C11orf58* on the binding of DNA to transcription factors; Table S18: Analysis of the effect of rs7951676 *C11orf58* on the binding of DNA to transcription factors; Table S19: Results of aggregated bioinformatic analysis of associations between *C11orf58* SNPs, cerebrovascular diseases and their intermediate phenotypes; Table S20: The most significant combinations of genotypes associated with the risk of developing IS; Table S21: The most significant genotype-environment combinations associated with the risk of developing IS. Figure S1: *C11orf58* expression levels in vessels, brain, and peripheral blood (GTExPortal data).
