# Supplementary material for: C11orf58 (Hero20) Gene Polymorphism: Contribution to Ischemic Stroke Risk and Interactions with Other Heat-Resistant Obscure Chaperones
Source: Biomedicines. 2024 Nov 14;12(11):2603. doi: 10.3390/biomedicines12112603 (PMC11592265; doi:10.3390/biomedicines12112603)
Supplement: Supplementary file 1 [file biomedicines-12-02603-s001.zip › biomedicines-3287349-supplementary.pdf]

Table S1: Characterization of *C11orf58* SNPs (NCBI data https://www.ncbi.nlm.nih.gov/).

SNP	Position	Variation type	EU population frequency	Clinical Significance	Gene : Consequence
rs6677* *C11orf58*	chr11:16755585	SNV	T=0.56 G=0.44	Not Reported in ClinVar	C11orf58 : 3 Prime UTR Variant
rs1846936 *C11orf58*	chr11:16738697	SNV	T=0.17 G=0.83	Not Reported in ClinVar	C11orf58 : 5 Prime UTR Variant
rs11024031 *C11orf58*	chr11:16751043	SNV	T=0.56 C=0.44	Not Reported in ClinVar	C11orf58 : Intron Variant
rs10766342 *C11orf58*	chr11:16739249	SNV	G=0.84 A=0.16	Not Reported in ClinVar	C11orf58 : Intron Variant
rs7928675 *C11orf58*	chr11:16740259	SNV	A=0.79 C=0.21	Not Reported in ClinVar	C11orf58 : Intron Variant
rs11024030 *C11orf58*	chr11:16749066	SNV	T=0.82 C=0.18	Not Reported in ClinVar	C11orf58 : Intron Variant
rs11024032* *C11orf58*	chr11:16751953	SNV	C=0.82 T=0.18	Not Reported in ClinVar	C11orf58 : Intron Variant
rs4757430 *C11orf58*	chr11:16754315	SNV	A=0.66 G=0.34	Not Reported in ClinVar	C11orf58 : Intron Variant
rs7951676* *C11orf58*	chr11:16747703	SNV	G=0.89 T=0.11	Not Reported in ClinVar	C11orf58 : Intron Variant
rs11826990 *C11orf58*	chr11:16739177	SNV	T=0.81 G=0.19	Not Reported in ClinVar	C11orf58 : Intron Variant
rs3203295 *C11orf58*	chr11:16740086	SNV	A=0.81 C=0.19	Not Reported in ClinVar	C11orf58 : Intron Variant
rs10832676 *C11orf58*	chr11:16747341	SNV	A=0.82 G=0.18	Not Reported in ClinVar	C11orf58 : Intron Variant

rs4757429 *C11orf58*	chr11:16742895	SNV	C=0.82 T=0.18	Not Reported in ClinVar	C11orf58 : Intron Variant
rs3802963* *C11orf58*	chr11:16739562	SNV	C=0.19 G=0.81	Not Reported in ClinVar	C11orf58 : Intron Variant
rs10734249 *C11orf58*	chr11:16753707	SNV	G=0.17 A=0.83	Not Reported in ClinVar	C11orf58 : Intron Variant
SNV – single nucleotide variant.					

Table S2: Transcripts and proteins of Hero genes (NCBI and UniProt data).

Gene symbol	Transcript[1]	Length (nt)[1]	Protein[1]	Length (aa)[1]	Mass (Da)[2]	Protein name[1]	Isoform [1]
C11orf58	NM_014267.6	3920	NP_055082.1	183	20.333	small acidic protein	
	XM_017017143.3	7837	XP_016872632.1	139			X1
	XM_047426310.1	6128	XP_047282266.1	139			X1
C19orf53	NM_014047.3	897	NP_054766.1	99	10.577	Leydig Cell Tumor 10 KDa Protein Homolog	
BBLN (C9orf16)	NM_024112.4	704	NP_077017.1	83	9.054	bublin coiled-coil protein	
SERF2	NR_037672.1	2477				small EDRK-rich factor 2	
	NM_001199877.2	3429	NP_001186806.1	59	6.9		c
	NM_001199875.1	3048	NP_001186804.1	170	18.315		a
	NM_001199876.1	2937	NP_001186805.1	133	14.334		b
	NM_001199878.2	2771	NP_001186807.1	45	5.216		d
	NM_001018108.4	2543	NP_001018118.1	59	6.9		c
SERBP1	NM_015640.4	6663	NP_056455.3	387	42.427	SERPINE 1 mRNA-	4

	NM_001018068.2	6708	NP_001018078.1	402	44.257	binding protein 1	3
	NM_001018069.2	6681	NP_001018079.1	393	43.135		2
	NM_001018067.2	6726	NP_001018077.1	408	44.965		1
nt – nucleotides, aa – aminoamides;<br>1 – data from National Library of Medicine (https://www.ncbi.nlm.nih.gov/)<br>2 – data from UniProt (https://www.uniprot.org/).							

Table S3: Primers and probes designed for the study

rs6677*<br>*C11orf58*	5′-TTGTTATAGTAGAGCTGTTCATTATGG-3′<br>5′-TGTGAAAAGTACTTGCTCTCATGTT-3′<br>5′-(FAM)CTTAGTTTCCTGTTACT(RTQ1)-3′<br>5′-(ROX)CTTAGTTGCCTGTTACT(BHQ2)-3′
rs1846936<br>*C11orf58*	5′-GTGACGACTGTGGCAGAGAA-3′<br>5′-GAAAAATGCCGAACCCTCTC-3′<br>5′-(FAM)CTTGGGCCCTTGGCGGAT(RTQ1)-3′<br>5′-(ROX)CTTGGGCCCGTGGCGGAT(BHQ2)-3′
rs11024031<br>*C11orf58*	5′- CCACCTGAACTTACCATTTTG-3′<br>5′-AGGAAGCCGTCTCTCTTCTG-3′<br>5′-(FAM)TGAGAAGATAGACACAGAGC(RTQ1)-3′<br>5′-(ROX)TGAGAAGACAGACACAGAGC(BHQ2)-3′
rs10766342<br>*C11orf58*	5′-TTTCTCGGGCCTTTGTACTC-3′<br>5′-CAGCTAAAGTTAACTCTCAACAGCA-3′<br>5′-(FAM)TCGTGCACGTTTGTACAG(RTQ1)-3′<br>5′-(ROX)TCGTGCACATTTGTACAG(BHQ2)-3′
rs7928675<br>*C11orf58*	5′-GCTGTTACATTGTTTTCCTCCTG-3′<br>5′-GACCTCTTCCGAAGCTGTAAA-3′<br>5′-(FAM)CGTGACAGAAAACTCCA(RTQ1)-3′<br>5′-(ROX)CGTGACAGCAAACTCCA(BHQ2)-3′
rs11024030<br>*C11orf58*	5′-TGTCATAGTTGGCATTTGGTTT-3′<br>5′-CCCAAATTCATTTTCAGTTTTT-3′<br>5′-(FAM)TAAACTAGTTGCAGGGCCTAA(RTQ1)-3′<br>5′-(ROX)TAAACTAGCTGCAGGGCCTAA(BHQ2)-3′
rs11024032*<br>*C11orf58*	5′-GGATCAGCTGCTTAAGATCACA-3′<br>5′-GACAGGAGGTTGGAAATGGA-3′<br>5′-(FAM)CAAAGTGGAACATCCTGAGAT(RTQ1)-3′<br>5′-(ROX)CAAAGTGGAATATCCTGAGAT(BHQ2)-3′
rs4757430<br>*C11orf58*	5′- TTGTTTCTTTCAGGAAAATATAATACC-3′<br>5′-TGCCACTACATTCCAGCCTGT-3′<br>5′-(FAM)TGATCTTCAGAGCACCTC(RTQ1)-3′<br>5′-(ROX)TGATCTTCAGGGCACCTC(BHQ2)-3′

rs7951676*<br>*C11orf58*	5′- TGTCAGGCTAAATCTCATTTGCT-3′<br>5′-CATTTTCTTTCCTAAAGGCCAGT-3′<br>5′-(FAM)CTCTCCATGAGATATACTTGA(RTQ1)-3′<br>5′-(ROX)CTCTCCATGATATATACTTGA(BHQ2)-3′
rs11826990<br>*C11orf58*	5′-CTGAGAGGGTCGAGGAGTGA-3′<br>5′-TCGACATTGTCACGGGAAC-3′<br>5′-(FAM)TACTCAGTATAGAGAC(RTQ1)-3′<br>5′-(ROX)TACTCAGGATAGAGAC(BHQ2)-3′
rs3203295<br>*C11orf58*	5′-CGACTTCAGCTCTGCAGAAAG-3′<br>5′-GGCAGCCAGGTTACTCAAAA-3′<br>5′-(FAM)CTGCTACTGGATTTTAGATCT(RTQ1)-3′<br>5′-(ROX)CTGCTACTGGCTTTTAGATCT(BHQ2)-3′
rs10832676<br>*C11orf58*	5′-CACTTGAAGTTTTTCGGTAGTATCA-3′<br>5′-AAACACCATGTGAAGGTGAGAA-3′<br>5′-(FAM)CTTCACAGATGATTG(RTQ1)-3′<br>5′-(ROX)CTTCACAGGTGATTG(BHQ2)-3′
rs4757429<br>*C11orf58*	5′- TTCTTTGTTTTCCTGATTTTGATG-3′<br>5′-CCCAAGTATATGGAAGGCTGA-3′<br>5′-(FAM)CAAATCTTGCATACTCA(RTQ1)-3′<br>5′-(ROX)CAAATCTTGTATACTCA(BHQ2)-3′
rs3802963*<br>*C11orf58*	5′-TCCTCAGCCCTCACCTCATA-3′<br>5′-TGGGCTAGGAGCTAGGACAA-3′<br>5′-(FAM)TACTGCACTGCCG(RTQ1)-3′<br>5′-(ROX)TACTGCAGTGCCG(BHQ2)-3′
rs10734249<br>*C11orf58*	5′-ACGTTGGATGGAACCTGTCTCAAAAAAGGG-3′<br>5′-ACGTTGGATGTCACCTGGCCTGTTTCTAAG-3′<br>5′-TCTAATGATTAAGCAGAATTTTCA-3′

Table S4: Used bioinformatic tools for bioinformatics analysis of the functional effects of *C11orf58* gene and *C11orf58* SNPs

Name	Link
GTExportal	http://www.gtexportal.org/ (accessed on December 20, 2023)
eQTLGen	https://www.eqtlgen.org/phase1.html (accessed on December 20, 2023)
QTLbase	http://www.mulinlab.org/qtlbase (accessed on December 20, 2023)
HaploReg (v4.2)	https://pubs.broadinstitute.org/mammals/haploreg/haploreg.php (accessed on December 21, 2023)
atSNP Function Prediction tool	http://atsnp.biostat.wisc.edu/ (accessed on December 21, 2023)
Gene Ontology	https://geneontology.org/ (accessed on December 21, 2023)
Cerebrovascular Disease Knowledge Portal and Cardiovascular	https://cd.hugeamp.org/ (accessed on December 22, 2023)<br>https://cvd.hugeamp.org/ (accessed on December 22, 2023)

Disease Knowledge Portal	

Table S5: Distribution of *C11orf58* genotypes in ischemic stroke patients/healthy controls and their correspondence to the Hardy-Weinberg equilibrium

SNP	Genotypes	Controls	H_o (H_e)[1]	P[2]	IS patients	H_o (H_e)[3]	P[4]
rs6677	T/T	358 (29.1%)	0.4805 (0.4980)	0.23	263 (29.4%)	0.4911 (0.4969)	0.74
	T/G	590 (48%)			440 (49.1%)		
	G/G	280 (22.8%)			193 (21.5%)		
	Maf(G)	0.468			0.461		
rs1846936	G/G	778 (64.1%)	0.3100 (0.3243)	0.13	552 (63.5%)	0.3314 (0.3189)	0.29
	G/T	376 (31%)			288 (33.1%)		
	T/T	59 (4.9%)			29 (3.3%)		
	Maf(T)	0.204			0.199		
rs11024031	T/T	356 (30.4%)	0.4838 (0.4958)	0.41	260 (29.9%)	0.4937 (0.4959)	0.89
	T/C	567 (48.4%)			430 (49.4%)		
	C/C	249 (21.2%)			181 (20.8%)		
	Maf(C)	0.454			0.455		
rs10766342	G/G	834 (69.2%)	0.2753 (0.2833)	0.31	558 (64.5%)	0.3156 (0.3165)	0.91
	G/A	332 (27.5%)			273 (31.6%)		
	A/A	40 (3.3%)			34 (3.9%)		
	Maf(A)	0.171			0.197		
rs7928675	A/A	826 (68.3%)	0.2950 (0.2820)	0.13	626 (72%)	0.2529 (0.2606)	0.36
	A/C	357 (29.5%)			220 (25.3%)		
	C/C	27 (2.2%)			24 (2.8%)		
	Maf(C)	0.170			0.154		
rs11024030	T/T	843 (68.4%)	0.2790 (0.2911)	0.14	555 (63.9%)	0.3245 (0.3189)	0.67
	C/T	344 (27.9%)			282 (32.5%)		
	C/C	46 (3.7%)			32 (3.7%)		
	Maf(C)	0.177			0.199		

rs11024032	C/C	842 (68.8%)	0.2747 (0.2877)	0.11	549 (63.2%)	0.3283 (0.3240)	0.75
	T/C	336 (27.5%)			285 (32.8%)		
	T/T	45 (3.7%)			34 (3.9%)		
	Maf(T)	0.174			0.203		
rs4757430	A/A	362 (29.4%)	0.4748 (0.4980)	0.11	254 (29.4%)	0.4890 (0.4971)	0.63
	A/G	584 (47.5%)			423 (48.9%)		
	G/G	284 (23.1%)			188 (21.7%)		
	Maf(G)	0.468			0.462		
rs7951676	G/G	827 (68.7%)	0.2868 (0.2811)	0.54	622 (72.2%)	0.2520 (0.2572)	0.6
	G/T	345 (28.7%)			217 (25.2%)		
	T/T	31 (2.6%)			22 (2.6%)		
	Maf(T)	0.169			0.152		
rs11826990	T/T	842 (69.6%)	0.2678 (0.2825)	0.083	551 (63.8%)	0.3241 (0.3203)	0.83
	T/G	324 (26.8%)			280 (32.4%)		
	G/G	44 (3.6%)			33 (3.8%)		
	Maf(G)	0.170			0.200		
rs3203295	A/A	844 (69%)	0.2764 (0.2844)	0.32	548 (64%)	0.3224 (0.3183)	0.75
	A/C	338 (27.6%)			276 (32.2%)		
	C/C	41 (3.4%)			32 (3.7%)		
	Maf(C)	0.172			0.199		
rs10832676	A/A	846 (69.3%)	0.2703 (0.2848)	0.087	548 (63.1%)	0.3295 (0.3247)	0.75
	A/G	330 (27%)			286 (33%)		
	G/G	45 (3.7%)			34 (3.9%)		
	Maf(G)	0.172			0.204		
rs4757429	C/C	842 (69.4%)	0.2704 (0.2831)	0.13	557 (64.3%)	0.3187 (0.3169)	0.92
	C/T	328 (27%)			276 (31.9%)		
	T/T	43 (3.5%)			33 (3.8%)		
	Maf(T)	0.171			0.197		
rs3802963	G/G	782 (64.2%)	0.3161 (0.3199)	0.65	548 (62.8%)	0.3383 (0.3229)	0.17
	C/G	385 (31.6%)			295 (33.8%)		

	C/C	51 (4.2%)			29 (3.3%)		
	Maf(C)	0.200			0.202		
rs10734249	A/A	814 (69.3%)	0.2826 (0.2768)	0.53	497 (64.6%)	0.3277 (0.3076)	0.079
	G/A	332 (28.3%)			252 (32.8%)		
	G/G	29 (2.5%)			20 (2.6%)		
	Maf(G)	0.166			0.190		

[1] – observed (Ho) and expected (He) heterozygosity in healthy controls; [2] – P-HWE in healthy controls; [3] – observed (Ho) and expected (He) heterozygosity in IS patients; [4] – P-HWE in IS patients

Table S6: Results of the analysis of the associations between *C11orf58* SNPs and ischemic stroke risk.

Genetic variant	Effect allele	Other allele	N	OR [95% CI][1]	P[2]
rs6677* *C11orf58*	G	T	2124	0.96 [0.85-1.08]	0.5
rs1846936 *C11orf58*	T	G	2082	0.96 [0.82-1.13]	0.64
rs11024031 *C11orf58*	C	T	2043	1.00 [0.88-1.13]	0.95
rs10766342 *C11orf58*	A	G	2071	**1.21 [1.03-1.43]**	**0.02**
rs7928675 *C11orf58*	C	A	2080	0.89 [0.75-1.07]	0.21
rs11024030 *C11orf58*	C	T	2102	1.16 [0.99-1.37]	0.064
rs11024032* *C11orf58*	T	C	2091	**1.22 [1.04-1.44]**	**0.01**
rs4757430 *C11orf58*	G	A	2095	0.97 [0.85-1.10]	0.59
rs7951676* *C11orf58*	T	G	2064	0.88 [0.74-1.05]	0.17
rs11826990 *C11orf58*	G	T	2074	**1.25 [1.06-1.47]**	**0.007**
rs3203295 *C11orf58*	C	A	2079	**1.22 [1.04-1.44]**	**0.016**
rs10832676 *C11orf58*	G	A	2089	**1.26 [1.07-1.48]**	**0.006**
rs4757429 *C11orf58*	T	C	2079	**1.21 [1.03-1.42]**	**0.02**
rs3802963* *C11orf58*	C	G	2090	1.02 [0.87-1.19]	0.85
rs10734249 *C11orf58*	G	A	1997	1.19 [1.00-1.41]	0.051

All calculations were performed relative to the minor alleles (Effect allele) with adjustment for sex, age, smoking; 1 - odds ratio and 95% confidence interval; 2– P- value; tag SNPs are marked with an asterisk; statistically significant differences are marked in bold.

Table S7: Subgroup's analysis of the associations between *C11orf58* SNPs and IS risk depending on smoking status, fruit and vegetable intake.

Genetic variant	Effect allele	Other allele	N	OR [95% CI][1]	P[2] (P_{bonf})	N	OR [95% CI][1]	P[2] (P_{bonf})
			Nonsmokers			Smokers		
rs6677* *C11orf58*	G	T	1314	0.98 [0.84-1.15]	0.8	810	0.95 [0.78-1.15]	0.59
rs1846936 *C11orf58*	T	G	1296	0.97 [0.80-1.18]	0.75	786	1.00 [0.77-1.29]	1
rs11024031 *C11orf58*	C	T	1271	1.02 [0.87-1.20]	0.78	772	0.96 [0.78-1.17]	0.67
rs10766342 *C11orf58*	A	G	1292	1.09 [0.88-1.33]	0.43	779	**1.42 [1.09-1.86]**	**0.009**
rs7928675 *C11orf58*	C	A	1299	1.01 [0.81-1.26]	0.94	781	**0.73 [0.56-0.96]**	**0.025**
rs11024030 *C11orf58*	C	T	1309	1.06 [0.87-1.30]	0.56	793	**1.33 [1.02-1.72]**	**0.03**
rs11024032* *C11orf58*	T	C	1306	1.12 [0.92-1.37]	0.26	785	**1.39 [1.07-1.81]**	**0.01**
rs4757430 *C11orf58*	G	A	1309	0.96 [0.82-1.12]	0.6	786	0.99 [0.81-1.21]	0.91
rs7951676* *C11orf58*	T	G	1288	0.97 [0.78-1.21]	0.81	776	**0.75 [0.57-0.99]**	**0.04**
rs11826990 *C11orf58*	G	T	1293	1.11 [0.90-1.36]	0.33	781	**1.48 [1.13-1.93]**	**0.004**
rs3203295 *C11orf58*	C	A	1300	1.11 [0.90-1.36]	0.33	779	**1.40 [1.07-1.83]**	**0.01**
rs10832676 *C11orf58*	G	A	1307	1.10 [0.90-1.34]	0.36	782	**1.53 [1.17-2.00]**	**0.002**
rs4757429 *C11orf58*	T	C	1295	1.12 [0.92-1.38]	0.27	784	**1.32 [1.01-1.71]**	**0.04**
rs3802963* *C11orf58*	C	G	1304	1.03 [0.84-1.25]	0.8	786	1.03 [0.79-1.34]	0.82
rs10734249 *C11orf58*	G	A	1161	1.18 [0.94-1.49]	0.15	783	1.19 [0.91-1.56]	0.21
			Normal fruit/vegetable intake [f-]			Low fruit/vegetable intake[f+]		
rs6677* *C11orf58*	G	T	1628	0.93 [0.79-1.09]	0.37 (0.74)	1687	1.01 [0.87-1.18]	0.86 (1.72)
rs1846936 *C11orf58*	T	G	1611	1.08 [0.89-1.31]	0.46 (0.92)	1660	0.86 [0.70-1.04]	0.11 (0.22)
rs11024031 *C11orf58*	C	T	1569	0.96 [0.82-1.13]	0.63 (1.26)	1622	1.05 [0.90-1.22]	0.54 (1.08)
rs10766342	A	G	1603	1.14	0.22	1650	**1.26**	**0.02**

C11orf58				[0.93-1.40]	(0.44)		**[1.04-1.53]**	**(0.04)**
rs7928675 *C11orf58*	C	A	1609	0.82 [0.65-1.03]	0.08 (0.16)	1657	0.93 [0.75-1.15]	0.49 (0.98)
rs11024030 *C11orf58*	C	T	1629	1.07 [0.87-1.31]	0.53 (1.06)	1683	**1.26 [1.05-1.52]**	**0.016 (0.032)**
rs11024032* *C11orf58*	T	C	1619	1.18 [0.96-1.44]	0.11 (0.22)	1671	**1.26 [1.04-1.52]**	**0.02 (0.04)**
rs4757430 *C11orf58*	G	A	1624	0.96 [0.82-1.12]	0.58 (1.16)	1677	1.01 [0.87-1.17]	0.94 (1.88)
rs7951676* *C11orf58*	T	G	1596	0.82 [0.65-1.02]	0.07 (0.14)	1647	0.91 [0.74-1.12]	0.38 (0.76)
rs11826990 *C11orf58*	G	T	1605	1.19 [0.97-1.46]	0.09 (0.18)	1656	**1.26 [1.05-1.53]**	**0.017 (0.034)**
rs3203295 *C11orf58*	C	A	1616	1.15 [0.94-1.42]	0.17 (0.34)	1663	**1.26 [1.04-1.53]**	**0.02 (0.04)**
rs10832676 *C11orf58*	G	A	1616	1.19 [0.97-1.45]	0.096 (0.19)	1670	**1.30 [1.07-1.57]**	**0.007 (0.014)**
rs4757429 *C11orf58*	T	C	1607	1.16 [0.94-1.42]	0.16 (0.32)	1661	**1.25 [1.03-1.51]**	**0.02 (0.04)**
rs3802963* *C11orf58*	C	G	1616	1.10 [0.91-1.34]	0.33 (0.66)	1668	0.91 [0.75-1.11]	0.35 (0.7)
rs10734249 *C11orf58*	G	A	1510	1.22 [0.97-1.52]	0.09 (0.18)	1553	1.10 [0.88-1.36]	0.42 (0.84)

1 – odds ratio and 95% confidence interval; 2 – P- value (P- value with Bonferroni correction). All calculations were performed relative to the minor allele (Effect allele). Statistically significant differences are marked in bold; tag SNPs are marked with an asterisk.

Table S8: Haplotype frequencies of the tag SNPs *C19orf58* gene and their associations with ischemic stroke risk

	rs3802963	rs7951676	rs11024032	rs6677	Healthy Controls	Patients with IS	OR [95% CI]	P-value
Entire group								
H1	G	G	C	G	0.4549	0.4337	1.00	---
H2	C	G	C	T	0.1832	0.1826	1.04 [0.87 - 1.24]	0.69
H3	G	G	T	T	0.1636	0.1896	**1.22 [1.02 - 1.46]**	**0.026**
H4	G	T	C	T	0.1589	0.1264	0.84 [0.69 - 1.02]	0.075
H5	G	G	C	T	0.016	0.0269	1.50 [0.99 - 2.29]	0.059
H6	C	T	C	T	0.0085	0.0142	1.81 [0.90 - 3.64]	0.094
rare	*	*	*	*			**1.73 [1.05 - 2.85]**	**0.032**
Global haplotype association p-value: =0.0013								
Females								
H1	G	G	C	G	0.4543	0.4264	1.00	---
H2	C	G	C	T	0.1921	0.1834	1.00 [0.79 - 1.28]	0.99
H3	G	G	T	T	0.1739	0.1967	1.1 [0.93 - 1.51]	0.16
H4	G	T	C	T	0.1475	0.1235	0.89 [0.68 - 1.17]	0.41
H5	G	G	C	T	0.0121	0.0234	1.73	0.091

							[0.92 - 3.27]	
H6	C	T	C	T	0.0081	0.0154	1.84 [0.73 - 4.65]	0.2
rare	*	*	*	*			**2.66 [1.35 - 5.23]**	**0.0049**
Global haplotype association p-value: =0.012								
Smokers								
H1	G	G	C	G	0.4583	0.4514	1.00	---
H2	C	G	C	T	0.174	0.179	1.04 [0.78 - 1.39]	0.8
H3	G	G	T	T	0.1461	0.1941	**1.34 [1.01 - 1.79]**	**0.042**
H4	G	T	C	T	0.1761	0.1242	**0.71 [0.52 - 0.95]**	**0.022**
H5	G	G	C	T	0.0188	0.0222	1.08 [0.55 - 2.14]	0.82
H6	C	T	C	T	0.0066	0.0131	1.76 [0.53 - 5.80]	0.35
rare	*	*	*	*			0.86 [0.37 - 1.99]	0.72
Global haplotype association p-value: =0.043								
Nonsmokers								
H1	G	G	C	G	0.455	0.4187	1.00	---
H2	C	G	C	T	0.1872	0.1852	1.06 [0.85 - 1.33]	0.6
H3	G	G	T	T	0.1705	0.1834	1.13 [0.91 - 1.42]	0.27
H4	G	T	C	T	0.1505	0.129	0.93	0.56

							[0.72 - 1.19]	
H5	G	G	C	T	0.0148	0.0318	**1.90 [1.14 - 3.19]**	**0.015**
H6	C	T	C	T	0.0089	0.0133	1.59 [0.67 - 3.75]	0.29
rare	*	*	*	*			**3.03 [1.63 - 5.63]**	**0.0005**
Global haplotype association p-value: =8×10^{-4}								
Low fruit and vegetable intake								
H1	G	G	C	G	0.4549	0.4402	1.00	---
H2	C	G	C	T	0.1832	0.1591	0.88 [0.71 - 1.11]	0.28
H3	G	G	T	T	0.1636	0.1944	1.19 [0.97 - 1.47]	0.1
H4	G	T	C	T	0.1589	0.1258	0.82 [0.65 - 1.04]	0.11
H5	G	G	C	T	0.016	0.0297	**1.61 [1.00 - 2.57]**	**0.048**
H6	C	T	C	T	0.0085	0.0176	**2.11 [1.00 - 4.46]**	**0.049**
rare	*	*	*	*			**2.16 [1.27 - 3.68]**	**0.0046**
Global haplotype association p-value: =0.00025								

Table S9: Linkage disequilibrium indicators between IS-associated SNPs *C19orf58* in smokers

Smokers										
SNP	rs11826990	rs10766342	rs3203295	rs7928675	rs4757429	rs10832676	rs7951676	rs11024030	rs11024032
rs11826990	.	0.1389	0.146	-0.029	0.1407	0.1447	-0.0287	0.1435	0.1417
		0.9426′	0.9901′	0.998′	0.9586′	0.986′	0.9983′	0.9829′	0.9686′
rs10766342	.	.	0.1394	-0.0146	0.1348	0.1397	-0.0161	0.1384	0.1371
			0.9464′	0.5038′	0.9196′	0.9528′	0.5591′	0.9493′	0.938′
rs3203295	.	.	.	-0.029	0.1408	0.146	-0.0288	0.1443	0.1432
				0.9981′	0.9572′	0.9925′	0.9984′	0.9858′	0.9763′
rs7928675	.	.	.	.	-0.0248	-0.0297	0.1316	-0.0304	-0.0301
					0.8335′	0.9982′	0.9818′	0.9982′	0.9981′
rs4757429	.	.	.	.	.	0.1415	-0.0254	0.14	0.1383
						0.942′	0.8624′	0.9364′	0.9227′
rs10832676	.	.	.	.	.	.	-0.0294	0.1459	0.1445
							0.9984′	0.9764′	0.965′
rs7951676	.	.	.	.	.	.	.	-0.0301	-0.0298
								0.9985′	0.9984′
rs11024030	.	.	.	.	.	.	.	.	0.1505
									0.9938′
rs11024032	.	.	.	.	.	.	.	.	.
Note: P-values for all the LD-test were < 0.001.									

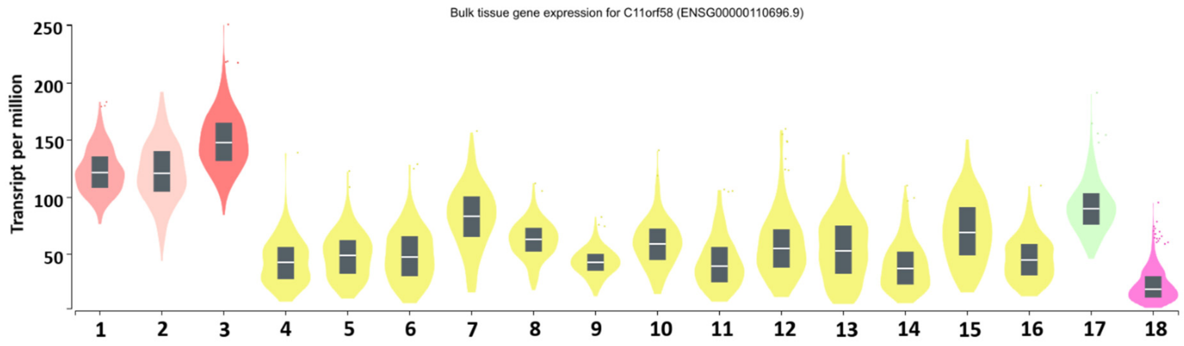

Figure S1: *C11orf58* expression levels in vessels, brain, and peripheral blood (GTExPortal data). Note: 1 – Artery – Aorta, 2 – Artery – Coronary, 3 – Artery – Tibial, 4 – Brain – Amygdala, 5 – Brain - Anterior cingulate cortex (BA24), 6 – Brain - Caudate (basal ganglia), 7 – Brain - Cerebellar Hemisphere, 8 – Brain – Cerebellum, 9 – Brain – Cortex, 10 – Brain - Frontal Cortex (BA9), 11 – Brain – Hippocampus, 12 – Brain – Hypothalamus, 13 – Brain - Nucleus accumbens (basal ganglia), 14 – Brain - Putamen (basal ganglia), 15 – Brain - Spinal cord (cervical c-1), 16 – Brain - Substantia nigra, 17 – Pituitary, 18 – Whole Blood.

Table S10: Analysis of the effect of rs10766342 *C11orf58* on the binding of DNA to transcription factors

№	Ref/SNP allele[1]	TF[2]	GAIN /LOSS[3]	Motif[4]	P-Value SNP impact[5]	P-Value Ref[6]	P-Value SNP[7]
1	G/A	CEBP	gain	CEBP_2	0	0.074	0.000002
2	G/A	FOXC1	gain	FOXC1_5	0.00002	0.322	0.0004
3	G/A	FOXJ2	gain	FOXJ2_2	0.00004	0.107	0.009
4	G/A	FOXB1	gain	FOXB1_3	0.0001	0.123	0.0003
5	G/A	ARID3A	gain	ARID3A_1	0.001	0.130	0.006
6	G/A	SOX8	gain	SOX8_1	0.001	0.144	0.004
7	G/A	FOXC2	gain	FOXC2_3	0.001	0.093	0.002
8	G/A	FOXL1	gain	FOXL1_3	0.001	0.160	0.005
9	G/A	FOXL1	gain	FOXL1_1	0.001	0.125	0.002
10	G/A	GATA	gain	GATA_1	0.001	0.203	0.008
11	G/A	FOXD3	gain	FOXD3_4	0.001	0.711	0.006
12	G/A	FOXO6	gain	FOXO6_2	0.001	0.365	0.008
13	G/A	FOXG1	gain	FOXG1_2	0.002	0.656	0.009
14	G/A	SOX5	gain	SOX5_3	0.002	0.185	0.009
15	G/A	FOXA	gain	FOXA_disc2	0.002	0.058	0.004
16	G/A	FOXD3	gain	FOXD3_2	0.002	0.303	0.010
17	G/A	SOX13	gain	SOX13_1	0.003	0.125	0.007
18	G/A	POU2F1	gain	POU2F1_6	0.003	0.222	0.015

19	G/A	CEBPA	gain	CEBPA_2	0.004	0.466	0.025
20	G/A	SOX9	gain	SOX9_3	0.004	0.796	0.022
21	G/A	FOXL1	gain	FOXL1_4	0.004	0.245	0.005
22	G/A	FOXD1	gain	FOXD1_2	0.004	0.420	0.022
23	G/A	FOXD3	gain	FOXD3_1	0.004	0.408	0.013
24	G/A	FOXG1	gain	FOXG1_3	0.004	0.127	0.019
25	G/A	SOX15	gain	SOX15_1	0.005	0.176	0.016
26	G/A	FOXO1	gain	FOXO1_3	0.006	0.361	0.019
27	G/A	MYEF2	gain	MYEF2_4	0.006	0.279	0.029
28	G/A	HSF	gain	HSF_disc1	0.007	0.232	0.016
29	G/A	HOXB7	gain	HOXB7_1	0.007	0.415	0.032
30	G/A	FOXJ3	gain	FOXJ3_8	0.007	0.053	0.005
31	G/A	AP1	gain	AP1_6	0.007	0.241	0.019
32	G/A	HNF1A	gain	HNF1A_1	0.008	0.143	0.017
33	G/A	PDX1	gain	PDX1_1	0.008	0.360	0.039
34	G/A	FOXF1	gain	FOXF1_1	0.008	0.113	0.016
35	G/A	Foxa2	gain	MA0047.2	0.008	0.067	0.007
36	G/A	TLX2	gain	TLX2_2	0.009	0.133	0.019
37	G/A	ARID3A	gain	MA0151.1	0.009	0.139	0.010
38	G/A	CEBPA	gain	CEBPA_1	0.009	0.285	0.021
39	G/A	FOXO4	gain	FOXO4_4	0.009	0.178	0.008
40	G/A	SOX9	gain	SOX9_2	0.009	0.730	0.038
41	G/A	FOXA2	gain	FOXA2_3	0.010	0.199	0.017
42	G/A	FOXJ3	gain	FOXJ3_7	0.010	0.253	0.020
43	G/A	FOXA2	gain	FOXA2_2	0.011	0.064	0.005
44	G/A	PBX1	gain	PBX1_1	0.011	0.062	0.007
45	G/A	DBX2	gain	CEBP_2	0	0.074	0.000
46	G/A	CBX5	gain	FOXC1_5	0.00002	0.322	0.000
47	G/A	PPARG	gain	FOXJ2_2	0.00004	0.107	0.009
48	G/A	ONECUT1	gain	FOXB1_3	0.0001	0.123	0.0003
49	G/A	ARX	gain	ARID3A_1	0.001	0.130	0.006
50	G/A	LBX2	gain	SOX8_1	0.001	0.144	0.004
51	G/A	FOXK1	gain	FOXC2_3	0.001	0.093	0.002
52	G/A	Foxd3	gain	FOXL1_3	0.001	0.160	0.005
53	G/A	PDX1	gain	FOXL1_1	0.001	0.125	0.002
54	G/A	SOX8	gain	GATA_1	0.001	0.203	0.008
55	G/A	FOXJ2	gain	FOXD3_4	0.001	0.711	0.006
56	G/A	Sox5	gain	FOXO6_2	0.001	0.365	0.008
57	G/A	HOXA7	gain	FOXG1_2	0.002	0.656	0.009
58	G/A	MEF2A	gain	SOX5_3	0.002	0.185	0.009
59	G/A	FOXG1	gain	FOXA_disc2	0.002	0.058	0.004
60	G/A	SIX6	gain	FOXD3_2	0.002	0.303	0.010
61	G/A	FOXB1	gain	SOX13_1	0.003	0.125	0.007

62	G/A	SRF	gain	POU2F1_6	0.003	0.222	0.015
63	G/A	HNF1	gain	CEBPA_2	0.004	0.466	0.025
64	G/A	FOXO3	gain	SOX9_3	0.004	0.796	0.022
65	G/A	FOXA	gain	FOXL1_4	0.004	0.245	0.005
66	G/A	DMRT2	gain	FOXD1_2	0.004	0.420	0.022
67	G/A	FOXJ1	gain	FOXD3_1	0.004	0.408	0.013
68	G/A	SIX6	gain	FOXG1_3	0.004	0.127	0.019
69	G/A	FOXC1	gain	SOX15_1	0.005	0.176	0.016
70	G/A	SIX6	gain	FOXO1_3	0.006	0.361	0.019
71	G/A	SOX9	gain	MYEF2_4	0.006	0.279	0.029
72	G/A	FOXA1	gain	HSF_disc1	0.007	0.232	0.016
73	G/A	HOXA10	gain	HOXB7_1	0.007	0.415	0.032
74	G/A	POU5F1	gain	FOXJ3_8	0.007	0.053	0.005
75	G/A	SIX1	gain	AP1_6	0.007	0.241	0.019
76	G/A	LMX1B	gain	HNF1A_1	0.008	0.143	0.017
77	G/A	NKX2-5	gain	PDX1_1	0.008	0.360	0.039
78	G/A	REST	gain	FOXF1_1	0.008	0.113	0.016
79	G/A	SIX3	gain	MA0047.2	0.008	0.067	0.007
80	G/A	FOXO6	gain	TLX2_2	0.009	0.133	0.019
81	G/A	CDX2	gain	MA0151.1	0.009	0.139	0.010
82	G/A	DBX1	gain	CEBPA_1	0.009	0.285	0.021
83	G/A	FOXB1	gain	FOXO4_4	0.009	0.178	0.008
84	G/A	SOX12	gain	SOX9_2	0.009	0.730	0.038
85	G/A	NFATC1	loss	NFATC1_3	0.004	0.047	0.774
86	G/A	HOMEZ	loss	HOMEZ_2	0.004	0.008	0.147
87	G/A	HOMEZ	loss	HOMEZ_1	0.004	0.012	0.178
88	G/A	SOX9	loss	SOX9_5	0.005	0.012	0.091
89	G/A	SOX8	loss	SOX8_6	0.009	0.008	0.097
90	G/A	MYBL1	loss	MYBL1_5	0.010	0.028	0.283
91	G/A	CCDC6	loss	NFATC1_3	0.004	0.047	0.774
92	G/A	Gata1	loss	HOMEZ_2	0.004	0.008	0.147
93	G/A	HOXB9	loss	HOMEZ_1	0.004	0.012	0.178
94	G/A	FOXJ3	loss	SOX9_5	0.005	0.012	0.091
95	G/A	ARID3A	loss	SOX8_6	0.009	0.008	0.097
	A[8]	astrocyte fate commitment (GO:0060018; FDR = **0.0058**);<br>neuron fate specification (GO:0048665; FDR = **0.00031**);<br>monocyte differentiation (GO:0030224; FDR = **0.047**);<br>dopaminergic neuron differentiation (GO:0071542; FDR = **0.0076**);<br>artery morphogenesis (GO:0048844; FDR = **0.0018**);<br>regulation of vascular associated smooth muscle cell proliferation (GO:1904705; FDR = **0.028**);<br>positive regulation of neuron differentiation (GO:0045666; FDR = **0.0073**);					

		negative regulation of angiogenesis (GO:001652; FDR = **0.0096**);<br>neural precursor cell proliferation (GO:0061351; FDR = **0.01**);<br>oligodendrocyte differentiation (GO:0048709; FDR = **0.047**);<br>cellular response to transforming growth factor beta stimulus (GO:0071560; FDR = **0.033**);<br>regulation of neurogenesis (GO:0050767; FDR = **0.026**);<br>axon development (GO:0061564; FDR = **0.04**);<br>response to cytokine (GO:0034097; FDR = **0.017**).
	G[9]	astrocyte fate commitment (GO:0060018; FDR = **0.0027**).

1 – reference (Ref) / alternative (SNP) allele;
2 – TF - transcription factor;
3 – binding of TF to the reference (LOSS) / alternative (GAIN) allele;
4 – binding sites with high affinity for TF;
5 – p value statistically confirming the potential gain or loss of function of the genomic region with SNP in terms of transcription factor binding;
6 – p-value for assessing the binding of TF to the Ref allele;
7 – p-value for assessing the binding of TF to the SNP allele;
8 – biological processes pathogenetically significant for IS, in which TFs that bind to the SNP allele are jointly involved (data from the Gene Ontology resource; http://geneontology.org/);
9 – biological processes pathogenetically significant for IS, in which TFs that bind to reference allele are jointly involved (data from the Gene Ontology resource; http://geneontology.org/)

Table S11: Analysis of the effect of rs11024030 *C11orf58* on the binding of DNA to transcription factors

№	Ref/ SNP allele[1]	TF[2]	GAIN /LOSS[3]	Motif[4]	P-Value SNP impact[5]	P-Value Ref[6]	P-Value SNP[7]
1	T/C	E2F	gain	E2F_disc8	0	0.075	0
2	T/C	TFAP2	gain	TFAP2_disc 2	0	0.299	0.000001
3	T/C	PAX5	gain	PAX5_disc5	0.000001	0.346	0.00001
4	T/C	MYC	gain	MYC_disc9	0.00003	0.060	0.0002
5	T/C	CTCF	gain	CTCF_disc8	0.00003	0.136	0.002
6	T/C	TAL1	gain	TAL1_4	0.0002	0.192	0.026
7	T/C	ELF1	gain	ELF1_disc3	0.0003	0.077	0.002
8	T/C	TFAP2A	gain	TFAP2A_10	0.0005	0.114	0.003
9	T/C	TCF3	gain	TCF3_1	0.0007	0.125	0.014
10	T/C	SIN3A	gain	SIN3A_disc 6	0.001	0.059	0.005
11	T/C	TFAP2A	gain	TFAP2A_5	0.001	0.064	0.003
12	T/C	TFAP2A	gain	MA0003.2	0.002	0.099	0.003
13	T/C	Myod1	gain	MA0499.1	0.002	0.316	0.039

14	T/C	TFAP2C	gain	TFAP2C_4	0.002	0.160	0.006
15	T/C	TFAP2A	gain	TFAP2A_7	0.002	0.097	0.004
16	T/C	AP1	gain	AP1_disc10	0.002	0.290	0.029
17	T/C	REST	gain	REST_3	0.002	0.193	0.032
18	T/C	TFAP2C	gain	TFAP2C_2	0.002	0.075	0.005
19	T/C	REST	gain	REST_disc9	0.002	0.099	0.009
20	T/C	HEY1	gain	HEY1_disc2	0.002	0.290	0.035
21	T/C	TFCP2	gain	TFCP2_2	0.003	0.107	0.009
22	T/C	TFAP2B	gain	TFAP2B_1	0.003	0.087	0.008
23	T/C	TCF3	gain	TCF3_7	0.004	0.303	0.018
24	T/C	TFAP2A	gain	TFAP2A_2	0.004	0.095	0.009
25	T/C	TFAP2B	gain	TFAP2B_3	0.004	0.067	0.004
26	T/C	NHLH1	gain	NHLH1_5	0.004	0.695	0.029
27	T/C	BCL	gain	BCL_disc8	0.004	0.282	0.046
28	T/C	SMC3	gain	SMC3_disc2	0.004	0.105	0.011
29	T/C	TFAP4	gain	TFAP4_1	0.004	0.188	0.023
30	T/C	ZNF784	gain	ZNF784_1	0.005	0.291	0.018
31	T/C	CTCF	gain	CTCF_disc7	0.005	0.072	0.007
32	T/C	Tcf3	gain	MA0522.1	0.005	0.124	0.009
33	T/C	SP1	gain	SP1_6	0.005	0.419	0.014
34	T/C	TEAD2	gain	TEAD2_1	0.005	0.184	0.023
35	T/C	Atoh1	gain	MA0461.1	0.006	0.107	0.012
36	T/C	TFAP2C	gain	TFAP2C_8	0.006	0.176	0.009
37	T/C	FIGLA	gain	FIGLA_1	0.006	0.431	0.022
38	T/C	ETS	gain	ETS_disc9	0.006	0.268	0.028
39	T/C	EP300	gain	EP300_disc8	0.006	0.090	0.010
40	T/C	TFAP2A	gain	TFAP2A_11	0.006	0.251	0.037
41	T/C	TCF4	gain	TCF4_1	0.006	0.738	0.023
42	T/C	GABP	gain	GABP_1	0.007	0.281	0.034
43	T/C	TFAP2C	gain	TFAP2C_3	0.007	0.115	0.012
44	T/C	TFAP2C	gain	TFAP2C_5	0.008	0.246	0.037
45	T/C	TFAP2A	gain	E2F_disc8	0	0.075	0
46	T/C	E2F1	gain	TFAP2_disc 2	0	0.299	0.000001
47	T/C	E2F1	gain	PAX5_disc5	0.000001	0.346	0.00001
48	T/C	RFX5	gain	MYC_disc9	0.00003	0.060	0.0002
49	T/C	E2F1	gain	CTCF_disc8	0.00003	0.136	0.002
50	T/C	INSM1	gain	TAL1_4	0.0002	0.192	0.026
51	T/C	TFAP2E	gain	ELF1_disc3	0.0003	0.077	0.002
52	T/C	ID4	gain	TFAP2A_10	0.0005	0.114	0.003
53	T/C	RUNX1	gain	TCF3_1	0.0007	0.125	0.014
54	T/C	Myog	gain	SIN3A_disc 6	0.001	0.059	0.005

55	T/C	REST	gain	TFAP2A_5	0.001	0.064	0.003
56	T/C	TFAP2A	gain	MA0003.2	0.002	0.099	0.003
57	T/C	TFAP2B	gain	MA0499.1	0.002	0.316	0.039
58	T/C	SP3	gain	TFAP2C_4	0.002	0.160	0.006
59	T/C	E2F4	gain	TFAP2A_7	0.002	0.097	0.004
60	T/C	INSM1	gain	AP1_disc10	0.002	0.290	0.029
61	T/C	Tcf12	gain	REST_3	0.002	0.193	0.032
62	T/C	FOXO3	gain	TFAP2C_2	0.002	0.075	0.005
63	T/C	TBX5	gain	REST_disc9	0.002	0.099	0.009
64	T/C	REST	gain	HEY1_disc2	0.002	0.290	0.035
65	T/C	AIRE	gain	TFCP2_2	0.003	0.107	0.009
66	T/C	EBF1	gain	TFAP2B_1	0.003	0.087	0.008
67	T/C	MAZ	gain	TCF3_7	0.004	0.303	0.018
68	T/C	ASCL2	gain	TFAP2A_2	0.004	0.095	0.009
69	T/C	MTF1	gain	TFAP2B_3	0.004	0.067	0.004
70	T/C	NHLH1	gain	NHLH1_5	0.004	0.695	0.029
71	T/C	GRHL1	gain	BCL_disc8	0.004	0.282	0.046
72	T/C	TFAP2C	gain	SMC3_disc2	0.004	0.105	0.011
73	T/C	TFAP4	gain	TFAP4_1	0.004	0.188	0.023
74	T/C	CHD2	gain	ZNF784_1	0.005	0.291	0.018
75	T/C	SRF	gain	CTCF_disc7	0.005	0.072	0.007
76	T/C	DMRT1	gain	MA0522.1	0.005	0.124	0.009
77	T/C	TFAP4	gain	SP1_6	0.005	0.419	0.014
78	T/C	E2F1	gain	TEAD2_1	0.005	0.184	0.023
79	T/C	NR2F2	gain	MA0461.1	0.006	0.107	0.012
80	T/C	E2F3	gain	TFAP2C_8	0.006	0.176	0.009
81	T/C	TFAP2B	gain	FIGLA_1	0.006	0.431	0.022
82	T/C	REST	gain	ETS_disc9	0.006	0.268	0.028
83	T/C	ZBTB3	gain	EP300_disc8	0.006	0.090	0.010
84	T/C	E2F1	gain	TFAP2A_11	0.006	0.251	0.037
85	T/C	CTCF	gain	TCF4_1	0.006	0.738	0.023
86	T/C	TATA	gain	GABP_1	0.007	0.281	0.034
87	T/C	E2F1	gain	TFAP2C_3	0.007	0.115	0.012
88	T/C	FOXO4	gain	TFAP2C_5	0.008	0.246	0.037
89	T/C	NR2C2	loss	NR2C2_disc1	0.001	0.006	0.128
90	T/C	CTCF	loss	CTCF_disc4	0.002	0.021	0.561
91	T/C	FOXO1	loss	FOXO1_4	0.003	0.019	0.465
92	T/C	HOXA4	loss	HOXA4_1	0.005	0.020	0.340
93	T/C	PROP1	loss	PROP1_2	0.005	0.046	0.945
94	T/C	BATF	loss	BATF_disc2	0.006	0.028	0.231
95	T/C	ATF3	loss	NR2C2_disc1	0.001	0.006	0.128

96	T/C	TCF12	loss	CTCF_disc4	0.002	0.021	0.561
97	T/C	RUNX2	loss	FOXO1_4	0.003	0.019	0.465
98	T/C	ZNF350	loss	HOXA4_1	0.005	0.020	0.340
99	T/C	Mafb	loss	PROP1_2	0.005	0.046	0.945
100	T/C	SIN3A	loss	BATF_disc2	0.006	0.028	0.231
	C[8]	negative regulation of gliogenesis (GO:0014014; FDR = **0.0164**); positive regulation of neuron differentiation (GO:0045666; FDR = **0.0000336**); positive regulation of neuron apoptotic process (GO:0043525; FDR = **0.0339**); response to hypoxia (GO:0001666; FDR = **0.00788**); response to oxidative stress (GO:0006979; FDR = **0.03**); neuron differentiation (GO:0030182; FDR = **0.00885**); cellular response to stress (GO:0033554; FDR = **0.0166**).					
	T[9]	-					

1 – reference (Ref) / alternative (SNP) allele;
2 – TF - transcription factor;
3 – binding of TF to the reference (LOSS) / alternative (GAIN) allele;
4 – binding sites with high affinity for TF;
5 – p value statistically confirming the potential gain or loss of function of the genomic region with SNP in terms of transcription factor binding;
6 – p-value for assessing the binding of TF to the Ref allele;
7 – p-value for assessing the binding of TF to the SNP allele;
8 – biological processes pathogenetically significant for IS, in which TFs that bind to the SNP allele are jointly involved (data from the Gene Ontology resource; http://geneontology.org/);
9 – biological processes pathogenetically significant for IS, in which TFs that bind to reference allele are jointly involved (data from the Gene Ontology resource; http://geneontology.org/)

Table S12: Analysis of the effect of rs11024032 *C11orf58* on the binding of DNA to transcription factors

№	Ref/ SNP allele[1]	TF[2]	GAIN /LOSS[3]	Motif[4]	P-Value SNP impact[5]	P-Value Ref[6]	P-Value SNP[7]
1	C/T	TEAD1	gain	MA0090.1	0.0001	0.158	0.012
2	C/T	NR1H4	gain	NR1H4_1	0.0002	0.082	0.008
3	C/T	FOXH1	gain	MA0479.1	0.002	0.113	0.005
4	C/T	POU2F1	gain	POU2F1_2	0.002	0.112	0.002
5	C/T	CEBP	gain	CEBP_1	0.002	0.739	0.017
6	C/T	NR1H4	gain	NR1H4_2	0.003	0.140	0.020
7	C/T	CCDC6	gain	CCDC6_1	0.004	0.272	0.012
8	C/T	POU1F1	gain	POU1F1_2	0.005	0.421	0.024
9	C/T	TEAD1	gain	TEAD1_2	0.005	0.118	0.010

10	C/T	SIX1	gain	SIX1_1	0.006	0.291	0.026
11	C/T	CRX	gain	CRX_2	0.007	0.177	0.011
12	C/T	GATA	gain	GATA_1	0.007	0.557	0.032
13	C/T	OBOX6	gain	OBOX6_1	0.007	0.076	0.006
14	C/T	POU2F2	gain	POU2F2_4	0.008	0.310	0.036
15	C/T	NFAT5	gain	NFAT5_1	0.008	0.213	0.018
16	C/T	NFKB	gain	NFKB_disc1	0.009	0.052	0.007
17	C/T	RELA	gain	RELA_2	0.011	0.091	0.008
18	C/T	RUNX1	gain	RUNX1_5	0.012	0.220	0.029
19	C/T	AP1	gain	AP1_disc1	0.015	0.436	0.048
20	C/T	TEAD4	gain	TEAD4_1	0.024	0.210	0.021
21	C/T	GATA1	gain	GATA1_2	0.028	0.640	0.036
22	C/T	HMGA1	gain	HMGA1_1	0.029	0.108	0.006
23	C/T	GSC2	gain	GSC2_1	0.031	0.396	0.050
24	C/T	SOX10	gain	SOX10_3	0.035	0.150	0.031
25	C/T	EP300	gain	EP300_disc10	0.040	0.169	0.037
26	C/T	IRF7	gain	IRF7_3	0.049	0.160	0.038
27	C/T	AR	loss	AR_6	0.00004	0.00005	0.118
28	C/T	NFAT	loss	NFAT_1	0.0001	0.011	0.259
29	C/T	AR	loss	MA0007.2	0.001	0.004	0.154
30	C/T	NR3C1	loss	MA0113.2	0.001	0.004	0.083
31	C/T	PGR	loss	PGR_2	0.001	0.023	0.328
32	C/T	EP300	loss	EP300_disc4	0.002	0.008	0.136
33	C/T	AR	loss	AR_2	0.003	0.005	0.178
34	C/T	IRF1	loss	IRF1_3	0.005	0.008	0.143
35	C/T	EP300	loss	EP300_disc5	0.007	0.037	0.534
36	C/T	BATF	loss	BATF_disc2	0.008	0.015	0.134
37	C/T	AR	loss	AR_8	0.011	0.012	0.112
38	C/T	BACH2	loss	BACH2_1	0.011	0.041	0.776
39	C/T	STAT	loss	STAT_disc3	0.012	0.039	0.279
40	C/T	Spz1	loss	MA0111.1	0.012	0.022	0.216
41	C/T	NR3C1	loss	NR3C1_5	0.013	0.025	0.446
42	C/T	AR	loss	AR_7	0.014	0.009	0.072
43	C/T	STAT2::STAT1	loss	MA0517.1	0.017	0.022	0.183
44	C/T	Spi1	loss	MA0080.3	0.020	0.038	0.318
45	C/T	HINFP	loss	MA0131.1	0.020	0.041	0.407
46	C/T	SPI1	loss	SPI1_3	0.023	0.024	0.190
47	C/T	NANOG	loss	NANOG_disc3	0.027	0.012	0.102
48	C/T	CACD	loss	CACD_1	0.032	0.038	0.231
49	C/T	AR	loss	AR_3	0.042	0.019	0.111

50	C/T	SPI1	loss	SPI1_1	0.043	0.035	0.238
	T[8]	positive regulation of leukocyte adhesion to vascular endothelial cell (GO:1904996; FDR = **0.0393)**; positive regulation of NIK/NF-kappaB signaling (GO:1901224; FDR = **0.0109**); positive regulation of cytokine production (GO:0001819; FDR = **0.0217**).					
	C[9]	-					

1 – reference (Ref) / alternative (SNP) allele;
2 – TF - transcription factor;
3 – binding of TF to the reference (LOSS) / alternative (GAIN) allele;
4 – binding sites with high affinity for TF;
5 – p value statistically confirming the potential gain or loss of function of the genomic region with SNP in terms of transcription factor binding;
6 – p-value for assessing the binding of TF to the Ref allele;
7 – p-value for assessing the binding of TF to the SNP allele;
8 – biological processes pathogenetically significant for IS, in which TFs that bind to the SNP allele are jointly involved (data from the Gene Ontology resource; http://geneontology.org/);
9 – biological processes pathogenetically significant for IS, in which TFs that bind to reference allele are jointly involved (data from the Gene Ontology resource; http://geneontology.org/)

Table S13: Analysis of the effect of rs11826990 *C11orf58* on the binding of DNA to transcription factors

№	Ref/SNP allele[1]	TF[2]	GAIN /LOSS[3]	Motif[4]	P-Value SNP impact[5]	P-Value Ref[6]	P-Value SNP[7]
1	T/G	GATA2	gain	GATA2_2	0	0.391	0.004
2	T/G	GATA	gain	GATA_1	0.0002	0.290	0.008
3	T/G	REST	gain	REST_4	0.0004	0.172	0.008
4	T/G	REST	gain	MA0138.2	0.0008	0.158	0.008
5	T/G	REST	gain	REST_disc3	0.001	0.361	0.012
6	T/G	GATA2	gain	GATA2_1	0.003	0.954	0.008
7	T/G	GATA3	gain	GATA3_1	0.003	0.830	0.009
8	T/G	GATA1	gain	GATA1_4	0.003	0.336	0.013
9	T/G	COMP1	gain	COMP1_1	0.003	0.199	0.018
10	T/G	Erg	gain	MA0474.1	0.003	0.311	0.014
11	T/G	ETS	gain	ETS_1	0.003	0.569	0.029
12	T/G	FLI1	gain	MA0475.1	0.004	0.301	0.016
13	T/G	TAL1::GATA1	gain	MA0140.2	0.004	0.539	0.050
14	T/G	Ets1	gain	MA0098.2	0.005	0.378	0.021

15	T/G	GATA	gain	GATA_disc3	0.005	0.653	0.027
16	T/G	ALX4	gain	ALX4_1	0.006	0.089	0.007
17	T/G	ETS1	gain	ETS1_3	0.007	0.635	0.014
18	T/G	FEV	gain	FEV_2	0.007	0.329	0.023
19	T/G	GATA1	gain	GATA1_1	0.007	0.919	0.023
20	T/G	IRF2	gain	MA0051.1	0.008	0.119	0.016
21	T/G	NR1H4	gain	NR1H4_2	0.008	0.262	0.043
22	T/G	ETS	gain	ETS_2	0.010	0.592	0.024
23	T/G	IKZF2	gain	IKZF2_3	0.011	0.731	0.048
24	T/G	ELF5	gain	MA0136.1	0.011	0.411	0.025
25	T/G	ERF	gain	ERF_1	0.011	0.747	0.049
26	T/G	ZEB1	gain	ZEB1_1	0.014	0.291	0.025
27	T/G	TFCP2L1	gain	TFCP2L1_1	0.014	0.432	0.048
28	T/G	ETS1	gain	ETS1_6	0.014	0.327	0.018
29	T/G	TFCP2	gain	TFCP2_5	0.016	0.389	0.045
30	T/G	FLI1	gain	FLI1_3	0.016	0.217	0.017
31	T/G	CCNT2	gain	CCNT2_disc1	0.017	0.312	0.041
32	T/G	RUNX1	gain	RUNX1_1	0.017	0.303	0.041
33	T/G	FLI1	gain	FLI1_1	0.018	0.256	0.019
34	T/G	ETV2	gain	ETV2_1	0.018	0.382	0.033
35	T/G	GATA3	gain	GATA3_2	0.019	0.606	0.026
36	T/G	ETV5	gain	ETV5_1	0.026	0.369	0.029
37	T/G	IRF2	gain	IRF2_2	0.028	0.189	0.026
38	T/G	IKZF2	gain	IKZF2_1	0.028	0.545	0.037
39	T/G	HDAC2	gain	GATA2_2	0	0.391	0.004
40	T/G	SIN3A	gain	GATA_1	0.0002	0.290	0.008
41	T/G	RUNX1	gain	REST_4	0.0004	0.172	0.008
42	T/G	ERG	gain	MA0138.2	0.0008	0.158	0.008
43	T/G	ETV4	gain	REST_disc3	0.001	0.361	0.012
44	T/G	ERG	gain	GATA2_1	0.003	0.954	0.008
45	T/G	FOXD1	gain	GATA3_1	0.003	0.830	0.009
46	T/G	ETS1	gain	GATA1_4	0.003	0.336	0.013
47	T/G	PAX1	gain	COMP1_1	0.003	0.199	0.018
48	T/G	MEF2C	gain	MA0474.1	0.003	0.311	0.014
49	T/G	MAFF	gain	ETS_1	0.003	0.569	0.029
50	T/G	RFX2	gain	MA0475.1	0.004	0.301	0.016
51	T/G	FOXJ2	loss	FOXJ2_2	0.00004	0.034	0.306
52	T/G	POU6F1	loss	POU6F1_2	0.003	0.042	0.510
53	T/G	RFX3	loss	RFX3_4	0.004	0.028	0.787
54	T/G	ETS	loss	ETS_disc3	0.006	0.005	0.057
55	T/G	IRF5	loss	IRF5_2	0.009	0.043	0.607

56	T/G	NKX3-1	loss	MA0124.1	0.010	0.009	0.141
57	T/G	FOXJ3	loss	FOXJ3_6	0.010	0.035	0.266
58	T/G	MYC	loss	MYC_disc8	0.011	0.029	0.927
59	T/G	SOX5	loss	SOX5_2	0.014	0.045	0.541
60	T/G	GZF1	loss	GZF1_1	0.018	0.014	0.116
61	T/G	MEF2A	loss	MA0052.2	0.019	0.020	0.162
62	T/G	MEF2A	loss	MEF2A_4	0.027	0.044	0.281
63	T/G	RFX2	loss	FOXJ2_2	0.00004	0.034	0.306
64	T/G	Rfx1	loss	POU6F1_2	0.003	0.042	0.510
	G[8]	positive regulation of neuron differentiation (GO:0045666; FDR = **0.0000746**); regulation of interleukin-2 production (GO:0032663; FDR = **0.0135**); regulation of blood vessel endothelial cell migration (GO:0043535; FDR = **0.0324**); positive regulation of endothelial cell migration (GO:0010595; FDR = **0.0466**); regulation of cellular response to growth factor stimulus (GO:0090287; FDR = **0.0103**); neuron development (GO:0048666; FDR **= 0.00364**); negative regulation of apoptotic process (GO:0043066; FDR **= 0.0248**).					
	T[9]	-					

1 – reference (Ref) / alternative (SNP) allele;
2 – TF - transcription factor;
3 – binding of TF to the reference (LOSS) / alternative (GAIN) allele;
4 – binding sites with high affinity for TF;
5 – p value statistically confirming the potential gain or loss of function of the genomic region with SNP in terms of transcription factor binding;
6 – p-value for assessing the binding of TF to the Ref allele;
7 – p-value for assessing the binding of TF to the SNP allele;
8 – biological processes pathogenetically significant for IS, in which TFs that bind to the SNP allele are jointly involved (data from the Gene Ontology resource; http://geneontology.org/);
9 – biological processes pathogenetically significant for IS, in which TFs that bind to reference allele are jointly involved (data from the Gene Ontology resource; http://geneontology.org/)

Table S14: Analysis of the effect of rs3203295 *C11orf58* on the binding of DNA to transcription factors

№	Ref/SNP allele[1]	TF[2]	GAIN /LOSS[3]	Motif[4]	P-Value SNP impact[5]	P-Value Ref[6]	P-Value SNP[7]
1	A/C	TFAP2A	gain	TFAP2A_4	0	0.130	0
2	A/C	TFAP2A	gain	TFAP2A_1	0	0.130	0

3	A/C	TFAP2	gain	TFAP2_3	0.00001	0.269	0.0003
4	A/C	NR2E1	gain	NR2E1_4	0.0005	0.106	0.003
5	A/C	RAD21	gain	RAD21_disc 7	0.001	0.064	0.004
6	A/C	TCF7L2	gain	TCF7L2_1	0.002	0.180	0.007
7	A/C	GTF2A	gain	GTF2A_1	0.002	0.150	0.013
8	A/C	TCF12	gain	TCF12_disc 2	0.005	0.446	0.028
9	A/C	MEF2	gain	MEF2_disc3	0.005	0.136	0.015
10	A/C	MEIS3	gain	MEIS3_4	0.009	0.740	0.047
11	A/C	TFAP2A	gain	TFAP2A_7	0.009	0.083	0.022
12	A/C	TFAP2	gain	TFAP2_2	0.010	0.387	0.045
13	A/C	REST	gain	REST_disc5	0.010	0.230	0.040
14	A/C	NR2E1	gain	NR2E1_3	0.010	0.277	0.012
15	A/C	MYC	gain	MYC_disc8	0.012	0.502	0.020
16	A/C	NR2F1	gain	NR2F1_3	0.013	0.252	0.050
17	A/C	MYOG	gain	MYOG_1	0.020	0.282	0.042
18	A/C	TCF12	gain	TCF12_disc 4	0.022	0.279	0.031
19	A/C	TFAP2	gain	TFAP2_disc 2	0.022	0.218	0.044
20	A/C	TCF12	gain	TCF12_1	0.024	0.121	0.024
21	A/C	TFAP2C	gain	TFAP2C_8	0.026	0.136	0.019
22	A/C	BARHL2	gain	BARHL2_6	0.028	0.355	0.041
23	A/C	TATA	gain	TATA_disc8	0.029	0.242	0.031
24	A/C	NR4A2	gain	NR4A2_4	0.030	0.181	0.024
25	A/C	ZNF384	gain	ZNF384_1	0.033	0.205	0.043
26	A/C	ESRRA	gain	ESRRA_disc 2	0.034	0.155	0.024
27	A/C	GCM1	gain	GCM1_4	0.035	0.124	0.018
28	A/C	MYC	gain	MYC_disc7	0.036	0.258	0.040
29	A/C	TATA	gain	TATA_disc7	0.036	0.058	0.011
30	A/C	TFCP2	gain	TFCP2_1	0.038	0.070	0.013
31	A/C	BARHL2	gain	BARHL2_3	0.038	0.351	0.049
32	A/C	TFAP2B	gain	TFAP2B_3	0.039	0.151	0.030
33	A/C	GCM1	gain	GCM1_1	0.040	0.080	0.016
34	A/C	BARHL1	gain	BARHL1_3	0.040	0.325	0.045
35	A/C	HNF4	gain	TFAP2A_4	0	0.130	0
36	A/C	SPDEF	gain	TFAP2A_1	0	0.130	0
37	A/C	ZKSCAN3	gain	TFAP2_3	0.00001	0.269	0.0003
38	A/C	NFIC	gain	NR2E1_4	0.0005	0.106	0.003
39	A/C	MYC	loss	MYC_disc6	0.001	0.043	0.281

40	A/C	NR2C2	loss	NR2C2_disc1	0.001	0.002	0.050
41	A/C	NKX2-1	loss	NKX2-1_1	0.005	0.032	0.366
42	A/C	ELF1	loss	ELF1_3	0.005	0.012	0.502
43	A/C	GFI1B	loss	GFI1B_1	0.005	0.035	0.591
44	A/C	ZNF354C	loss	ZNF354C_1	0.010	0.008	0.191
45	A/C	NR1H2::RXRA	loss	MA0115.1	0.010	0.033	0.190
46	A/C	ZNF354C	loss	MA0130.1	0.011	0.013	0.184
47	A/C	NFATC1	loss	NFATC1_2	0.014	0.010	0.079
48	A/C	CTCF	loss	CTCF_disc3	0.023	0.040	0.303
49	A/C	IRF7	loss	IRF7_3	0.024	0.045	0.270
50	A/C	DMRTA2	loss	DMRTA2_1	0.025	0.032	0.230
51	A/C	HNF4A	loss	HNF4A_11	0.025	0.016	0.096
52	A/C	ETS1	loss	ETS1_3	0.026	0.046	0.766
53	A/C	REST	loss	REST_3	0.031	0.040	0.170
54	A/C	FLI1	loss	FLI1_2	0.039	0.036	0.212
55	A/C	Myb	loss	MYC_disc6	0.001	0.043	0.281
	C[8]	neuron apoptotic process (GO:0051402; FDR = **0.035**); regulation of neuron apoptotic process (GO:0043523; FDR = **0.0204**); positive regulation of apoptotic process (GO:0043065; FDR **= 0.0369**); nervous system development (GO:0007399; FDR = **0.000346**).					
	A[9]	regulation of monocyte differentiation (GO:0045655; FDR = **0.0237**).					

1 – reference (Ref) / alternative (SNP) allele;
2 – TF - transcription factor;
3 – binding of TF to the reference (LOSS) / alternative (GAIN) allele;
4 – binding sites with high affinity for TF;
5 – p value statistically confirming the potential gain or loss of function of the genomic region with SNP in terms of transcription factor binding;
6 – p-value for assessing the binding of TF to the Ref allele;
7 – p-value for assessing the binding of TF to the SNP allele;
8 – biological processes pathogenetically significant for IS, in which TFs that bind to the SNP allele are jointly involved (data from the Gene Ontology resource; http://geneontology.org/);
9 – biological processes pathogenetically significant for IS, in which TFs that bind to reference allele are jointly involved (data from the Gene Ontology resource; http://geneontology.org/)

Table S15: Analysis of the effect of rs10832676 *C11orf58* on the binding of DNA to transcription factors

№	Ref/ SNP allele[1]	TF[2]	GAIN /LOSS[3]	Motif[4]	P-Value SNP impact[5]	P-Value Ref[6]	P-Value SNP[7]

1	A/G	MYC	gain	MYC_1	0	0.067	0.0001
2	A/G	ZEB1	gain	ZEB1_5	0	0.335	0.001
3	A/G	ZEB1	gain	ZEB1_disc1	0.0001	0.065	0.001
4	A/G	NFE2	gain	NFE2_disc3	0.0002	0.076	0.002
5	A/G	ZIC3	gain	ZIC3_3	0.0002	0.282	0.007
6	A/G	HEY2	gain	HEY2_2	0.0002	0.901	0.003
7	A/G	RAR	gain	RAR_1	0.0004	0.696	0.009
8	A/G	HEY1	gain	HEY1_1	0.0005	0.429	0.004
9	A/G	HEY2	gain	HEY2_1	0.0005	0.321	0.003
10	A/G	NR3C1	gain	NR3C1_disc5	0.001	0.105	0.004
11	A/G	MYC	gain	MYC_disc9	0.001	0.138	0.008
12	A/G	SCRT1	gain	SCRT1_1	0.001	0.374	0.004
13	A/G	ZBTB7A	gain	ZBTB7A_2	0.001	0.324	0.005
14	A/G	ZIC4	gain	ZIC4_1	0.001	0.359	0.008
15	A/G	GLIS2	gain	GLIS2_2	0.001	0.603	0.012
16	A/G	RXRG	gain	RXRG_3	0.001	0.305	0.007
17	A/G	AP1	gain	AP1_disc5	0.002	0.154	0.011
18	A/G	ZNF784	gain	ZNF784_1	0.002	0.394	0.010
19	A/G	PAX4	gain	PAX4_3	0.002	0.518	0.006
20	A/G	PPARG	gain	PPARG_1	0.002	0.077	0.007
21	A/G	RARG	gain	RARG_1	0.002	0.549	0.006
22	A/G	SNAI2	gain	SNAI2_1	0.002	0.159	0.003
23	A/G	TBX5	gain	TBX5_1	0.002	0.388	0.013
24	A/G	HEY1	gain	HEY1_disc2	0.002	0.204	0.024
25	A/G	REST	gain	REST_disc5	0.003	0.202	0.021
26	A/G	RARB	gain	RARB_1	0.003	0.297	0.005
27	A/G	MYC	gain	MYC_disc10	0.003	0.455	0.037
28	A/G	TFCP2	gain	TFCP2_1	0.003	0.200	0.018
29	A/G	TBX5	gain	TBX5_4	0.003	0.934	0.017
30	A/G	ZIC3	gain	ZIC3_4	0.003	0.272	0.021
31	A/G	BRCA1	gain	BRCA1_disc1	0.004	0.342	0.021
32	A/G	ARNTL	gain	ARNTL_1	0.004	0.437	0.004
33	A/G	XBP1	gain	XBP1_3	0.005	0.859	0.031
34	A/G	NR2C2	gain	NR2C2_disc2	0.005	0.605	0.016
35	A/G	MYC::MAX	gain	MYC::MAX_4	0.006	0.158	0.012
36	A/G	NR2F1	gain	NR2F1_3	0.006	0.263	0.041
37	A/G	TBX20	gain	TBX20_1	0.006	0.324	0.038
38	A/G	NFKB	gain	NFKB_disc3	0.006	0.182	0.007
39	A/G	ZIC1	gain	ZIC1_3	0.007	0.187	0.018

40	A/G	REST	gain	REST_disc8	0.007	0.296	0.050
41	A/G	NR2F1	gain	NR2F1_2	0.008	0.053	0.011
42	A/G	ZIC1	gain	ZIC1_2	0.008	0.115	0.021
43	A/G	CTCF	gain	CTCF_disc9	0.008	0.267	0.041
44	A/G	TCF4	gain	TCF4_2	0.008	0.058	0.003
45	A/G	ESRRA	gain	ESRRA_1	0.009	0.580	0.025
46	A/G	Arnt	gain	MYC_1	0	0.067	0.0001
47	A/G	HIF1A	gain	ZEB1_5	0	0.335	0.001
48	A/G	ATOH1	gain	ZEB1_disc1	0.0001	0.065	0.001
49	A/G	Rxra	gain	NFE2_disc3	0.0002	0.076	0.002
50	A/G	TP53	gain	ZIC3_3	0.0002	0.282	0.007
51	A/G	HES7	gain	HEY2_2	0.0002	0.901	0.003
52	A/G	CLOCK::ARNTL	gain	RAR_1	0.0004	0.696	0.009
53	A/G	MYB	gain	HEY1_1	0.0005	0.429	0.004
54	A/G	ARNT	gain	HEY2_1	0.0005	0.321	0.003
55	A/G	MLX	gain	NR3C1_disc5	0.0006	0.105	0.004
56	A/G	NR2C2	gain	MYC_disc9	0.0006	0.138	0.008
57	A/G	ZIC2	gain	SCRT1_1	0.0009	0.374	0.004
58	A/G	KLF7	gain	ZBTB7A_2	0.0009	0.324	0.005
59	A/G	ZBTB33	gain	ZIC4_1	0.0009	0.359	0.008
60	A/G	USF	gain	GLIS2_2	0.001	0.603	0.012
61	A/G	BHLHE41	gain	RXRG_3	0.001	0.305	0.007
62	A/G	NR2F6	gain	AP1_disc5	0.002	0.154	0.011
63	A/G	ZIC3	gain	ZNF784_1	0.002	0.394	0.010
64	A/G	ARNT	gain	PAX4_3	0.002	0.518	0.006
65	A/G	SIN3A	gain	MA0103.2	0.002	0.053	0.003
66	A/G	RXRA	gain	PPARG_1	0.002	0.077	0.007
67	A/G	ALX4	gain	RARG_1	0.002	0.549	0.006
68	A/G	TCF3	gain	SNAI2_1	0.002	0.159	0.003
69	A/G	NR3C1	gain	TBX5_1	0.002	0.388	0.013
70	A/G	NR2F6	gain	HEY1_disc2	0.002	0.204	0.024
71	A/G	PKNOX2	gain	REST_disc5	0.003	0.202	0.021
72	A/G	BHLHA15	gain	RARB_1	0.003	0.297	0.005
73	A/G	ZIC3	gain	MYC_disc10	0.003	0.455	0.037
74	A/G	FOXA	gain	TFCP2_1	0.003	0.200	0.018
75	A/G	CUX1	gain	TBX5_4	0.003	0.934	0.017
76	A/G	MSC	gain	ZIC3_4	0.003	0.272	0.021
77	A/G	RXRA	gain	BRCA1_disc1	0.004	0.342	0.021
78	A/G	TLX2	gain	ARNTL_1	0.004	0.437	0.004
79	A/G	TAL1::TCF3	gain	XBP1_3	0.005	0.859	0.031

80	A/G	BHLHE40	gain	NR2C2_disc2	0.005	0.605	0.016
81	A/G	MAX	gain	MYC::MAX_4	0.006	0.158	0.012
82	A/G	DPRX	gain	NR2F1_3	0.006	0.263	0.041
83	A/G	Myc	gain	TBX20_1	0.006	0.324	0.038
84	A/G	NEUROG2	gain	NFKB_disc3	0.006	0.182	0.007
85	A/G	NR2F1	gain	ZIC1_3	0.007	0.187	0.018
86	A/G	BHLHE41	gain	REST_disc8	0.007	0.296	0.050
87	A/G	NRF1	gain	NR2F1_2	0.008	0.053	0.011
88	A/G	MYC::MAX	gain	ZIC1_2	0.008	0.115	0.021
89	A/G	BHLHE40	gain	CTCF_disc9	0.008	0.267	0.041
90	A/G	SP4	gain	TCF4_2	0.008	0.058	0.003
91	A/G	NR2F6	gain	ESRRA_1	0.009	0.580	0.025
92	A/G	SREBF1	gain	MYC_1	0	0.067	0.0001
93	A/G	ESR1	gain	ZEB1_5	0	0.335	0.001
94	A/G	SMC3	gain	ZEB1_disc1	0.00006	0.065	0.001
95	A/G	TGIF1	gain	NFE2_disc3	0.0002	0.076	0.002
96	A/G	TATA	gain	ZIC3_3	0.0002	0.282	0.007
97	A/G	MAX	gain	HEY2_2	0.0002	0.901	0.003
98	A/G	RARB	gain	RAR_1	0.0004	0.696	0.009
99	A/G	Mycn	gain	HEY1_1	0.0005	0.429	0.004
100	A/G	ZEB1	gain	MA0103.2	0.002	0.053	0.003
101	A/G	PBX1	loss	PBX1_1	0	0.001	0.7310
102	A/G	PBX1	loss	PBX1_4	0.003	0.003	0.092
103	A/G	ZBTB18	loss	ZBTB18_1	0.004	0.006	0.072
104	A/G	YY1	loss	YY1_6	0.007	0.027	0.484
105	A/G	GFI1B	loss	PBX1_1	0	0.001	0.731
106	A/G	USF	loss	PBX1_4	0.003	0.003	0.092
107	A/G	ZBTB18	loss	ZBTB18_1	0.004	0.006	0.072
108	A/G	RXRA	loss	YY1_6	0.007	0.027	0.484
109	A/G	MAX	loss	PBX1_1	0	0.001	0.731
	G[8]	oxidative stress-induced premature senescence (GO:0090403; FDR = **0.00787**); regulation of transcription from RNA polymerase II promoter in response to oxidative stress (GO:0043619; FDR = **0.000369**); positive regulation of transcription from RNA polymerase II promoter in response to hypoxia (GO:0061419; FDR = **0.0103**); arterial endothelial cell differentiation (GO:0060842; FDR = **0.0128**); peroxisome proliferator activated receptor signaling pathway (GO:0035357; FDR = **0.0218**); positive regulation of transcription from RNA polymerase II promoter in response to endoplasmic reticulum stress (GO:1990440; FDR = **0.0333**);					

		positive regulation of vascular endothelial growth factor receptor signaling pathway (GO:0030949; FDR **= 0.0415**); regulation of apoptotic process (GO:0042981; FDR = **0.0000166**); positive regulation of vascular endothelial growth factor production (GO:0010575; FDR = **0.00769**); positive regulation of protein acetylation (GO:1901985; FDR = **0.0102**); negative regulation of reactive oxygen species metabolic process (GO:2000378; FDR = **0.00127**); regulation of autophagy of mitochondrion (GO:1903146; FDR = **0.0136**); negative regulation of Notch signaling pathway (GO:0045746; FDR = **0.0146**); negative regulation of gliogenesis (GO:0014014; FDR = **0.0174**); negative regulation of transforming growth factor beta receptor signaling pathway (GO:0030512; FDR = **0.00124**); regulation of vascular associated smooth muscle cell proliferation (GO:1904705; FDR = **0.0432**); positive regulation of apoptotic process (GO:0043065; FDR = **0.00258**); cellular response to growth factor stimulus (GO:0071363; FDR = **0.0333**); negative regulation of apoptotic process (GO:0043066; FDR = **0.00658**); neuron differentiation (GO:0030182; FDR = **0.00239**).
	A[9]	-

1 – reference (Ref) / alternative (SNP) allele;
2 – TF - transcription factor;
3 – binding of TF to the reference (LOSS) / alternative (GAIN) allele;
4 – binding sites with high affinity for TF;
5 – p value statistically confirming the potential gain or loss of function of the genomic region with SNP in terms of transcription factor binding;
6 – p-value for assessing the binding of TF to the Ref allele;
7 – p-value for assessing the binding of TF to the SNP allele;
8 – biological processes pathogenetically significant for IS, in which TFs that bind to the SNP allele are jointly involved (data from the Gene Ontology resource; http://geneontology.org/);
9 – biological processes pathogenetically significant for IS, in which TFs that bind to reference allele are jointly involved (data from the Gene Ontology resource; http://geneontology.org/)

Table S16. Analysis of the effect of rs4757429 *C11orf58* on the binding of DNA to transcription factors

№	Ref/ SNP allele[1]	TF[2]	GAIN /LOSS[3]	Motif[4]	P-Value SNP impact[5]	P-Value Ref[6]	P-Value SNP[7]

1	C/T	HDAC2	gain	HDAC2_disc2	0.0002	0.136	0.003
2	C/T	FOXJ1	gain	FOXJ1_2	0.001	0.133	0.003
3	C/T	Foxa2	gain	MA0047.2	0.001	0.195	0.010
4	C/T	FOXO1	gain	FOXO1_2	0.001	0.379	0.025
5	C/T	FOXO6	gain	FOXO6_2	0.001	0.282	0.006
6	C/T	FOXF2	gain	MA0030.1	0.002	0.159	0.010
7	C/T	HOXC13	gain	HOXC13_3	0.002	0.632	0.009
8	C/T	MYC	gain	MYC_disc6	0.002	0.312	0.040
9	C/T	FOXF2	gain	FOXF2_2	0.002	0.152	0.011
10	C/T	FOXB1	gain	FOXB1_3	0.002	0.109	0.004
11	C/T	SIX5	gain	SIX5_disc3	0.003	0.053	0.006
12	C/T	TBP	gain	TBP_1	0.003	0.164	0.016
13	C/T	RUNX1	gain	RUNX1_4	0.003	0.055	0.003
14	C/T	FOXA2	gain	FOXA2_2	0.003	0.217	0.011
15	C/T	FOXP2	gain	MA0593.1	0.003	0.117	0.006
16	C/T	HOXD11	gain	HOXD11_2	0.003	0.301	0.019
17	C/T	ALX4	gain	ALX4_1	0.003	0.460	0.032
18	C/T	FOXC1	gain	FOXC1_7	0.003	0.665	0.010
19	C/T	FOXO4	gain	FOXO4_2	0.003	0.159	0.012
20	C/T	Mecom	gain	MA0029.1	0.003	0.160	0.016
21	C/T	FOXL1	gain	FOXL1_3	0.004	0.159	0.013
22	C/T	FOXA2	gain	FOXA2_3	0.004	0.257	0.014
23	C/T	Foxo1	gain	MA0480.1	0.004	0.207	0.009
24	C/T	FOXO3	gain	FOXO3_2	0.005	0.216	0.009
25	C/T	FOXJ3	gain	FOXJ3_1	0.005	0.215	0.013
26	C/T	FOXO1	gain	FOXO1_4	0.005	0.102	0.007
27	C/T	FOXK1	gain	FOXK1_1	0.006	0.280	0.019
28	C/T	HOXD13	gain	HOXD13_5	0.006	0.441	0.021
29	C/T	FOXA1	gain	FOXA1_2	0.006	0.189	0.014
30	C/T	FOXA	gain	FOXA_disc1	0.006	0.164	0.009
31	C/T	SRY	gain	SRY_3	0.006	0.399	0.012
32	C/T	FOXP1	gain	MA0481.1	0.007	0.292	0.024
33	C/T	FOXD3	gain	FOXD3_1	0.008	0.389	0.025
34	C/T	ZNF35	gain	ZNF35_1	0.008	0.287	0.035
35	C/T	RUNX1	gain	RUNX1_1	0.008	0.063	0.004
36	C/T	HOXA13	gain	HOXA13_3	0.009	0.121	0.007
37	C/T	SOX10	gain	HDAC2_disc2	0.0002	0.136	0.003
38	C/T	FOXO3	gain	FOXJ1_2	0.001	0.133	0.003
39	C/T	POU5F1	gain	MA0047.2	0.001	0.195	0.010
40	C/T	FOXJ3	gain	FOXO1_2	0.001	0.379	0.025
41	C/T	RBPJ	gain	FOXO6_2	0.001	0.282	0.006

42	C/T	EGR2	gain	MA0030.1	0.002	0.159	0.010
43	C/T	FOXC2	gain	HOXC13_3	0.002	0.632	0.009
44	C/T	HOXC11	gain	MYC_disc6	0.002	0.312	0.040
45	C/T	STAT	gain	FOXF2_2	0.002	0.152	0.011
46	C/T	FOXC1	gain	FOXB1_3	0.002	0.109	0.004
47	C/T	CEBPB	gain	SIX5_disc3	0.003	0.053	0.006
48	C/T	POU4F2	gain	TBP_1	0.003	0.164	0.016
49	C/T	MAFF	gain	RUNX1_4	0.003	0.055	0.003
50	C/T	MYC	gain	FOXA2_2	0.003	0.217	0.011
51	C/T	HOXD13	gain	MA0593.1	0.003	0.117	0.006
52	C/T	RUNX1	gain	HOXD11_2	0.003	0.301	0.019
53	C/T	PROP1	gain	ALX4_1	0.003	0.460	0.032
54	C/T	RUNX1	gain	FOXC1_7	0.003	0.665	0.010
55	C/T	MYC	gain	FOXO4_2	0.003	0.159	0.012
56	C/T	IRX4	gain	MA0029.1	0.003	0.160	0.016
57	C/T	POU	gain	FOXL1_3	0.004	0.159	0.013
58	C/T	CEBPA	gain	FOXA2_3	0.004	0.257	0.014
59	C/T	HOXC10	gain	MA0480.1	0.004	0.207	0.009
60	C/T	POU3F1	gain	FOXO3_2	0.005	0.216	0.009
61	C/T	POU2F3	gain	FOXJ3_1	0.005	0.215	0.013
62	C/T	POU2F2	gain	FOXO1_4	0.005	0.102	0.007
63	C/T	POU4F1	gain	FOXK1_1	0.006	0.280	0.019
64	C/T	HOXC12	gain	HOXD13_5	0.006	0.441	0.021
65	C/T	CEBPA	gain	FOXA1_2	0.006	0.189	0.014
66	C/T	POU2F2	gain	FOXA_disc1	0.006	0.164	0.009
67	C/T	FOXI1	gain	SRY_3	0.006	0.399	0.012
68	C/T	TEAD1	gain	MA0481.1	0.007	0.292	0.024
69	C/T	FOXD2	gain	FOXD3_1	0.008	0.389	0.025
70	C/T	NRL	gain	ZNF35_1	0.008	0.287	0.035
71	C/T	NFE2L2	gain	RUNX1_1	0.008	0.063	0.004
72	C/T	ZSCAN4	gain	HOXA13_3	0.009	0.121	0.007
73	C/T	PAX6	gain	HDAC2_disc2	0.0002	0.136	0.003
74	C/T	POU2F1	gain	FOXJ1_2	0.001	0.133	0.003
75	C/T	FOXF1	gain	MA0047.2	0.001	0.195	0.010
76	C/T	FOXO4	gain	FOXO1_2	0.001	0.379	0.025
77	C/T	POU5F1B	gain	FOXO6_2	0.001	0.282	0.006
78	C/T	HOXB13	gain	MA0030.1	0.002	0.159	0.010
79	C/T	IRX5	gain	HOXC13_3	0.002	0.632	0.009
80	C/T	ZSCAN4	gain	MYC_disc6	0.002	0.312	0.040
81	C/T	FOXD3	gain	FOXF2_2	0.002	0.152	0.011
82	C/T	CTCF	gain	FOXB1_3	0.002	0.109	0.004
83	C/T	FOXO3	gain	SIX5_disc3	0.003	0.053	0.006

84	C/T	NFE2	gain	TBP_1	0.003	0.164	0.016
85	C/T	FOXK1	gain	RUNX1_4	0.003	0.055	0.003
86	C/T	FOXL1	gain	FOXA2_2	0.003	0.217	0.011
87	C/T	CEBP	loss	CEBP_2	0	0.00002	0.149
88	C/T	CEBPB	loss	CEBPB_2	0.00002	0.000001	0.220
89	C/T	POU2F1	loss	POU2F1_9	0.001	0.002	0.068
90	C/T	POU2F1	loss	POU2F1_6	0.003	0.007	0.103
91	C/T	ZNF8	loss	ZNF8_1	0.004	0.008	0.124
92	C/T	MAFB	loss	MAFB_2	0.005	0.017	0.215
93	C/T	POU3F3	loss	POU3F3_2	0.005	0.008	0.112
94	C/T	HINFP	loss	HINFP_3	0.005	0.033	0.235
95	C/T	POU4F2	loss	POU4F2_2	0.006	0.009	0.089
96	C/T	CEBP	loss	CEBP_5	0.007	0.028	0.701
97	C/T	POU2F2	loss	POU2F2_5	0.007	0.004	0.075
98	C/T	POU1F1	loss	POU1F1_2	0.007	0.020	0.278
99	C/T	POU3F3	loss	POU3F3_3	0.008	0.007	0.070
100	C/T	POU5F1	loss	POU5F1_3	0.009	0.032	0.256
101	C/T	FOXK1	loss	CEBP_2	0	0.00002	0.149
102	C/T	FOXF2	loss	CEBPB_2	0.00002	0.000001	0.220
103	C/T	FOXQ1	loss	POU2F1_9	0.001	0.002	0.068
104	C/T	SRY	loss	POU2F1_6	0.003	0.007	0.103
105	C/T	Pou5f1::Sox2	loss	ZNF8_1	0.004	0.008	0.124
106	C/T	Bach1::Mafk	loss	MAFB_2	0.005	0.017	0.215
107	C/T	IRX3	loss	POU3F3_2	0.005	0.008	0.112
108	C/T	ATF4	loss	HINFP_3	0.005	0.033	0.235
109	C/T	MAFK	loss	POU4F2_2	0.006	0.009	0.089
110	C/T	MAFK	loss	CEBP_5	0.007	0.028	0.701
111	C/T	IRX3	loss	POU2F2_5	0.007	0.004	0.075
112	C/T	ATF2	loss	POU1F1_2	0.007	0.020	0.278
113	C/T	FOXA1	loss	POU3F3_3	0.008	0.007	0.070
114	C/T	NFE2::MAF	loss	POU5F1_3	0.009	0.032	0.256
115	C/T	POU3F4	loss	CEBP_2	0	0.00002	0.149
116	C/T	GRHL1	loss	CEBPB_2	0.00002	0.000001	0.220
117	C/T	RUNX1	loss	POU2F1_9	0.001	0.002	0.068
118	C/T	FOXO1	loss	POU2F1_6	0.003	0.007	0.103
	T[8]	positive regulation of transcription from RNA polymerase II promoter in response to hypoxia (GO:0061419; FDR = **0.0126**);<br>integrated stress response signaling (GO:0140467; FDR = **0.000515**);<br>neuron fate specification (GO:0048665; FDR **= 0.0125**);<br>blood vessel remodeling (GO:0001974; FDR = **0.016**);<br>artery morphogenesis (GO:0048844; FDR **= 0.000365**);<br>peripheral nervous system development (GO:0007422; FDR = **0.000653**);					

		negative regulation of stress-activated MAPK cascade (GO:0032873; FDR = **0.0291**);<br>Notch signaling pathway (GO:0007219; FDR = **0.00278**);<br>positive regulation of neurogenesis (GO:0050769; FDR = **0.0434**).
	C[9]	BMP signaling pathway (GO:0030509; FDR = **0.0271**);<br>regulation of neuron apoptotic process (GO:0043523; FDR = **0.0207**);<br>positive regulation of cytokine production (GO:0001819; FDR **= 0.0311**);<br>positive regulation of programmed cell death (GO:0043068; FDR = **0.0425**);<br>neuron differentiation (GO:0030182; FDR = **0.0226**).
1 – reference (Ref) / alternative (SNP) allele;<br>2 – TF - transcription factor;<br>3 – binding of TF to the reference (LOSS) / alternative (GAIN) allele;<br>4 – binding sites with high affinity for TF;<br>5 – p value statistically confirming the potential gain or loss of function of the genomic region with SNP in terms of transcription factor binding;<br>6 – p-value for assessing the binding of TF to the Ref allele;<br>7 – p-value for assessing the binding of TF to the SNP allele;<br>8 – biological processes pathogenetically significant for IS, in which TFs that bind to the SNP allele are jointly involved (data from the Gene Ontology resource; http://geneontology.org/);<br>9 – biological processes pathogenetically significant for IS, in which TFs that bind to reference allele are jointly involved (data from the Gene Ontology resource; http://geneontology.org/)		

Table S17: Analysis of the effect of rs7928675 *C11orf58* on the binding of DNA to transcription factors

№	Ref/SNP allele[1]	TF[2]	GAIN /LOSS[3]	Motif[4]	P-Value SNP impact[5]	P-Value Ref[6]	P-Value SNP[7]
1	A/C	HIC1	gain	HIC1_2	0	0.244	0.000001
2	A/C	ZBTB14	gain	ZBTB14_1	0	0.179	0.000003
3	A/C	DBP	gain	DBP_1	0.001	0.124	0.001
4	A/C	ZBTB7A	gain	ZBTB7A_disc1	0.001	0.078	0.002
5	A/C	TFCP2	gain	TFCP2_1	0.001	0.070	0.005
6	A/C	NRL	gain	NRL_1	0.001	0.769	0.008
7	A/C	ETS	gain	ETS_disc7	0.002	0.105	0.006
8	A/C	CTCF	gain	CTCF_disc8	0.002	0.313	0.039
9	A/C	NFE2	gain	NFE2_disc4	0.002	0.108	0.007
10	A/C	E2F	gain	E2F_disc1	0.002	0.435	0.023
11	A/C	HNF4A	gain	MA0114.2	0.003	0.241	0.015

12	A/C	REST	gain	REST_disc4	0.003	0.369	0.040
13	A/C	E2F1	gain	E2F1_17	0.003	0.075	0.003
14	A/C	RAD21	gain	RAD21_disc8	0.003	0.187	0.022
15	A/C	MYC	gain	MYC_disc10	0.003	0.206	0.020
16	A/C	CTCF	gain	CTCF_disc5	0.003	0.196	0.027
17	A/C	HNF4A	gain	HNF4A_13	0.004	0.130	0.013
18	A/C	ETS1	gain	ETS1_6	0.004	0.526	0.018
19	A/C	MAFK	gain	MAFK_2	0.005	0.650	0.017
20	A/C	TBX21	gain	TBX21_6	0.006	0.277	0.029
21	A/C	MAFK	gain	MAFK_4	0.006	0.534	0.024
22	A/C	ESRRA	gain	ESRRA_disc4	0.006	0.204	0.022
23	A/C	RFX4	gain	RFX4_1	0.007	0.367	0.031
24	A/C	GABPA	gain	GABPA_3	0.007	0.509	0.023
25	A/C	RUNX	gain	RUNX_1	0.007	0.297	0.035
26	A/C	Mafb	gain	MA0117.1	0.008	0.337	0.015
27	A/C	RAD21	gain	RAD21_disc6	0.008	0.260	0.039
28	A/C	FLI1	gain	FLI1_1	0.009	0.752	0.032
29	A/C	MAFB	gain	MAFB_1	0.009	0.313	0.010
30	A/C	TFAP2	gain	TFAP2_2	0.010	0.323	0.029
31	A/C	ERF	gain	ERF_1	0.010	0.210	0.011
32	A/C	MAFB	gain	MAFB_3	0.010	0.591	0.039
33	A/C	INSM1	gain	INSM1_1	0.011	0.332	0.032
34	A/C	HIC1	gain	HIC1_1	0.011	0.570	0.045
35	A/C	SREBP	gain	SREBP_disc1	0.011	0.503	0.045
36	A/C	EGR1	gain	HIC1_2	0	0.244	0.000001
37	A/C	SPI1	gain	ZBTB14_1	0	0.179	0.000003
38	A/C	HNF4A	gain	DBP_1	0.001	0.124	0.001
39	A/C	TCF12	gain	ZBTB7A_disc1	0.001	0.078	0.002
40	A/C	IRF5	gain	TFCP2_1	0.001	0.070	0.005
41	A/C	CTCF	gain	NRL_1	0.001	0.769	0.008
42	A/C	RUNX2	gain	ETS_disc7	0.002	0.105	0.006
43	A/C	SPIB	gain	CTCF_disc8	0.002	0.313	0.039
44	A/C	TBX5	gain	NFE2_disc4	0.002	0.108	0.007
45	A/C	RAD21	gain	E2F_disc1	0.002	0.435	0.023
46	A/C	MTF1	gain	MA0114.2	0.003	0.241	0.015
47	A/C	SPDEF	gain	REST_disc4	0.003	0.369	0.040
48	A/C	STAT3	gain	E2F1_17	0.003	0.075	0.003

49	A/C	EGR1	gain	RAD21_disc 8	0.003	0.187	0.022
50	A/C	HMGN3	gain	MYC_disc10	0.003	0.206	0.020
51	A/C	TCF12	gain	CTCF_disc5	0.003	0.196	0.027
52	A/C	HNF4A	gain	HNF4A_13	0.004	0.130	0.013
53	A/C	PKNOX2	gain	ETS1_6	0.004	0.526	0.018
54	A/C	HSF1	gain	MAFK_2	0.005	0.650	0.017
55	A/C	REST	gain	TBX21_6	0.006	0.277	0.029
56	A/C	TGIF1	gain	MAFK_4	0.006	0.534	0.024
57	A/C	BCL	gain	ESRRA_disc 4	0.006	0.204	0.022
58	A/C	PPARG	gain	RFX4_1	0.007	0.367	0.031
59	A/C	RUNX2	gain	GABPA_3	0.007	0.509	0.023
60	A/C	HEY1	gain	RUNX_1	0.007	0.297	0.035
61	A/C	NFKB1	gain	MA0117.1	0.008	0.337	0.015
62	A/C	RXRA	gain	RAD21_disc 6	0.008	0.260	0.039
63	A/C	RXRA	gain	FLI1_1	0.009	0.752	0.032
64	A/C	AHR::ARNT	gain	MAFB_1	0.009	0.313	0.010
65	A/C	RUNX	gain	TFAP2_2	0.010	0.323	0.029
66	A/C	SIX5	loss	SIX5_disc4	0.0001	0.003	0.061
67	A/C	NFAT5	loss	NFAT5_1	0.001	0.008	0.638
68	A/C	REL	loss	REL_1	0.001	0.003	0.408
69	A/C	AP1	loss	AP1_1	0.001	0.014	0.369
70	A/C	REL	loss	REL_2	0.001	0.003	0.523
71	A/C	PTF1A	loss	PTF1A_1	0.002	0.004	0.052
72	A/C	STAT5A	loss	STAT5A_4	0.003	0.011	0.688
73	A/C	EWSR1::FLI1	loss	EWSR1::FLI1_1	0.003	0.034	0.207
74	A/C	Sox3	loss	MA0514.1	0.004	0.011	0.226
75	A/C	NFATC1	loss	NFATC1_2	0.005	0.032	0.371
76	A/C	STAT4	loss	STAT4_1	0.005	0.031	0.705
77	A/C	RREB1	loss	RREB1_1	0.006	0.023	0.122
78	A/C	STAT1	loss	STAT1_3	0.007	0.014	0.496
79	A/C	CCNT2	loss	CCNT2_disc1	0.007	0.039	0.654
80	A/C	NANOG	loss	NANOG_disc2	0.008	0.020	0.284
81	A/C	TATA	loss	SIX5_disc4	0.0001	0.003	0.061
82	A/C	MXI1	loss	NFAT5_1	0.001	0.008	0.638
83	A/C	JUN::FOS	loss	REL_1	0.001	0.003	0.408
84	A/C	E2F1	loss	AP1_1	0.001	0.014	0.369

85	A/C	TFAP2A	loss	REL_2	0.001	0.003	0.523
86	A/C	TBX21	loss	PTF1A_1	0.002	0.004	0.052
87	A/C	EGR4	loss	STAT5A_4	0.003	0.011	0.688
88	A/C	SPI1	loss	EWSR1::FLI1_1	0.003	0.034	0.207
89	A/C	RHOXF1	loss	MA0514.1	0.004	0.011	0.226
90	A/C	HNF4	loss	NFATC1_2	0.005	0.032	0.371
91	A/C	HSF1	loss	STAT4_1	0.005	0.031	0.705
92	A/C	NFKB1	loss	RREB1_1	0.006	0.023	0.122
93	A/C	REST	loss	STAT1_3	0.007	0.014	0.496
94	A/C	EGR1	loss	CCNT2_disc1	0.007	0.039	0.654
95	A/C	RARA	loss	NANOG_disc2	0.008	0.020	0.284
	C[8]	peroxisome proliferator activated receptor signaling pathway (GO:0035357; FDR = **0.0239**);<br>interleukin-6-mediated signaling pathway (GO:0070102; FDR = **0.0437**);<br>cellular response to interleukin-17 (GO:0097398; FDR = **0.0476**);<br>regulation of cholesterol transport (GO:0032374; FDR = **0.0329**);<br>cellular response to hypoxia (GO:0071456; FDR = **0.0172**);<br>transmembrane receptor protein serine/threonine kinase signaling pathway (GO:0007178; FDR = **0.000995**);<br>positive regulation of apoptotic process (GO:0043065; FDR = **0.0116**);<br>cellular response to growth factor stimulus (GO:0071363; FDR = **0.0442**).					
	A[9]	interleukin-9-mediated signaling pathway (GO:0038113; FDR = **0.00720**);<br>calcineurin-NFAT signaling cascade (GO:0033173; FDR = **0.0205**);<br>cellular response to angiotensin (GO:1904385; FDR = **0.0452**);<br>cytokine production (GO:0001816; FDR = **0.0479**);<br>receptor signaling pathway via JAK-STAT (GO:0007259; FDR = **0.0065**);<br>response to hypoxia (GO:0001666; FDR = **0.0303**);<br>regulation of neuron death (GO:0014041; FDR = **0.0373**);<br>cellular response to growth factor stimulus (GO:0071363; FDR = **0.0252**);<br>regulation of apoptotic process (GO:0042981; FDR **= 0.00556**);<br>cellular response to stress (GO:0033554; FDR = **0.0387**).					

1 – reference (Ref) / alternative (SNP) allele;
2 – TF - transcription factor;
3 – binding of TF to the reference (LOSS) / alternative (GAIN) allele;
4 – binding sites with high affinity for TF;
5 – p value statistically confirming the potential gain or loss of function of the genomic region with SNP in terms of transcription factor binding;
6 – p-value for assessing the binding of TF to the Ref allele;

7 – p-value for assessing the binding of TF to the SNP allele;
8 – biological processes pathogenetically significant for IS, in which TFs that bind to the SNP allele are jointly involved (data from the Gene Ontology resource; http://geneontology.org/);
9 – biological processes pathogenetically significant for IS, in which TFs that bind to reference allele are jointly involved (data from the Gene Ontology resource; http://geneontology.org/)

Table S18: Analysis of the effect of rs7951676 *C11orf58* on the binding of DNA to transcription factors

№	Ref/SNP allele[1]	TF[2]	GAIN /LOSS[3]	Motif[4]	P-Value SNP impact[5]	P-Value Ref[6]	P-Value SNP[7]
1	G/T	THAP1	gain	THAP1_disc1	0.00006	0.169	0.003
2	G/T	ZBTB6	gain	ZBTB6_1	0.0004	0.074	0.001
3	G/T	FOXB1	gain	FOXB1_3	0.002	0.752	0.027
4	G/T	YY1	gain	YY1_disc1	0.003	0.253	0.010
5	G/T	HDX	gain	HDX_1	0.004	0.511	0.030
6	G/T	BCL	gain	BCL_disc3	0.006	0.055	0.002
7	G/T	TBP	gain	MA0108.2	0.007	0.480	0.039
8	G/T	TATA	gain	TATA_disc1	0.007	0.163	0.006
9	G/T	HDAC2	gain	HDAC2_disc2	0.010	0.513	0.040
10	G/T	TBP	gain	TBP_5	0.010	0.482	0.041
11	G/T	TBP	gain	TBP_2	0.010	0.482	0.041
12	G/T	T	gain	T_3	0.011	0.375	0.041
13	G/T	FOXC1	gain	FOXC1_7	0.014	1.000	0.048
14	G/T	TAL1::GATA1	gain	MA0140.2	0.015	0.151	0.014
15	G/T	FOXB1	gain	FOXB1_4	0.029	0.337	0.037
16	G/T	HLF	gain	HLF_4	0.029	0.400	0.046
17	G/T	NR1H4	gain	NR1H4_2	0.036	0.243	0.043
18	G/T	NKX3-1	gain	NKX3-1_2	0.041	0.509	0.049
19	G/T	TAL1	loss	TAL1_4	0	0.049	0.347
20	G/T	POU2F1	loss	POU2F1_6	0.001	0.002	0.083
21	G/T	MYCN	loss	MYCN_1	0.002	0.009	0.212
22	G/T	MYC::MAX	loss	MYC::MAX_3	0.002	0.007	0.146
23	G/T	GLIS3	loss	GLIS3_1	0.002	0.010	0.289
24	G/T	ZBTB49	loss	ZBTB49_1	0.003	0.039	0.372
25	G/T	EBF1	loss	EBF1_disc1	0.004	0.010	0.087
26	G/T	AP1	loss	AP1_disc9	0.006	0.010	0.136

27	G/T	EBF1	loss	MA0154.2	0.006	0.023	0.281
28	G/T	RAD21	loss	RAD21_disc 2	0.007	0.026	0.265
29	G/T	ATF3	loss	ATF3_disc2	0.010	0.023	0.215
30	G/T	CENPB	loss	CENPB_1	0.010	0.022	0.347
31	G/T	MLXIPL	loss	MLXIPL_1	0.011	0.021	0.354
32	G/T	MAX	loss	MA0058.2	0.013	0.019	0.179
33	G/T	USF1	loss	USF1_1	0.013	0.029	0.308
34	G/T	USF2	loss	MA0526.1	0.013	0.029	0.251
35	G/T	USF2	loss	USF2_1	0.018	0.020	0.238
36	G/T	MAX	loss	MAX_2	0.019	0.026	0.286
37	G/T	TFEB	loss	TFEB_1	0.019	0.024	0.243
38	G/T	USF1	loss	USF1_2	0.022	0.039	0.353
39	G/T	RXRA	loss	RXRA_disc2	0.026	0.048	0.258
40	G/T	MXI1	loss	MXI1_disc2	0.027	0.049	0.397
41	G/T	PAX5	loss	PAX5_2	0.027	0.044	0.223
42	G/T	GLIS2	loss	GLIS2_2	0.032	0.045	0.272
43	G/T	JDP2	loss	JDP2_6	0.033	0.047	0.357
44	G/T	Myc	loss	MA0147.2	0.035	0.045	0.336
45	G/T	AP1	loss	AP1_disc4	0.036	0.037	0.254
46	G/T	MAX	loss	MAX_5	0.038	0.031	0.212
47	G/T	BACH1	loss	BACH1_1	0.038	0.038	0.273
48	G/T	CREB1	loss	CREB1_10	0.039	0.020	0.205
49	G/T	ZIC1	loss	ZIC1_2	0.050	0.046	0.134
50	G/T	RAD21	loss	RAD21_disc 4	0.050	0.049	0.437
	T[8]	-					
	G[9]	-					

1 – reference (Ref) / alternative (SNP) allele;
2 – TF - transcription factor;
3 – binding of TF to the reference (LOSS) / alternative (GAIN) allele;
4 – binding sites with high affinity for TF;
5 – p value statistically confirming the potential gain or loss of function of the genomic region with SNP in terms of transcription factor binding;
6 – p-value for assessing the binding of TF to the Ref allele;
7 – p-value for assessing the binding of TF to the SNP allele;
8 – biological processes pathogenetically significant for IS, in which TFs that bind to the SNP allele are jointly involved (data from the Gene Ontology resource; http://geneontology.org/);
9 – biological processes pathogenetically significant for IS, in which TFs that bind to reference allele are jointly involved (data from the Gene Ontology resource; http://geneontology.org/)

Table S19. Results of aggregated bioinformatic analysis of associations between *C11orf58* SNPs, cerebrovascular diseases and their intermediate phenotypes.

№	SNP	Phenotype	P-Value	Beta (OR)	Sample Size
1.	rs10766342 *C11orf58* (G/**A**)	[1]Systolic blood pressure	0.001	Beta ▲0.0064	1 310 370
2.		[1]Diastolic blood pressure	0.002	Beta ▲0.0061	1 331 510
3.		[1]Pulse pressure	0.024	Beta ▲0.0041	882 509
4.		[2]Total cholesterol	0.0002	Beta ▼-0.0056	1 939 980
5.<br>6.		[2]LDL cholesterol	0.021	Beta ▼-0.0033	2 036 340
7.	rs11024030 *C11orf58* (T/**C**)	[1]Systolic blood pressure	0.001	Beta ▲0.0066	1 309 960
8.		[1]Diastolic blood pressure	0.003	Beta ▲0.0059	1 331 070
9.		[1]Pulse pressure	0.020	Beta ▲0.0043	882 098
10.		[1]Lacunar stroke	0.037	OR ▲1.0530	28 530
11.		[2]Total cholesterol	0.004	Beta ▼-0.0045	1 939 980
12.	rs11024032 *C11orf58* (C/**T**)	[1]TOAST small artery occlusion	0.030	OR ▲2.8959	254 558
13.		[2]Total cholesterol	3.64×10^{-4}	Beta ▼-0.0054	1 924 340
14.		[2]Systolic blood pressure	8.91×10^{-4}	Beta ▲0.0053	1 290 030
15.		[2]Diastolic blood pressure	0.003	Beta ▲0.0065	1 311 140
16.		[2]LDL cholesterol	0.020	Beta ▼-0.0033	2 023 590
17.		[2]Pulse pressure	0.020	Beta ▼-0.0013	880 709
18.	rs11826990 *C11orf58* (T/**G**)	[1]Systolic blood pressure	0.001	Beta ▲0.0064	1 310 240
19.		[1]Diastolic blood pressure	0.003	Beta ▲0.006	1 331 380
20.		[1]Pulse pressure	0.022	Beta ▲0.0042	882 385
21.		[2]Total cholesterol	0.0002	Beta ▼-0.0057	1 910 510
22.		[2]LDL cholesterol	0.016	Beta ▼-0.0035	2 007 360
23.	rs3203295 *C11orf58* (A/**C**)	[1]Systolic blood pressure	0.001	Beta ▲0.0064	1 309 000
24.		[1]Diastolic blood pressure	0.003	Beta ▲0.006	1 330 150
25.		[1]Pulse pressure	0.024	Beta ▲0.0041	881 153
26.		[1]Lacunar stroke	0.037	OR ▲1.0520	28 530
27.		[2]Total cholesterol	0.0001	Beta ▼-0.0057	1 940 420
28.		[2]LDL cholesterol	0.012	Beta ▼-0.0036	2 024 730
29.	rs10832676 *C11orf58* (A/**G**)	[1]Systolic blood pressure	0.001	Beta ▲0.0066	1 291 600
30.		[1]Diastolic blood pressure	0.002	Beta ▲0.0061	1 312 710
31.		[1]Pulse pressure	0.020	Beta ▲0.0043	882 275
32.		[1]Lacunar stroke	0.037	OR ▲1.0530	28 530
33.		[2]Total cholesterol	0.006	Beta ▼-0.0044	1 924 340
34.	rs4757429 *C11orf58* (C/**T**)	[1]Systolic blood pressure	0.001	Beta ▲0.0066	1 309 670
35.		[1]Diastolic blood pressure	0.003	Beta ▲0.006	1 330 790
36.		[1]Pulse pressure	0.018	Beta ▲0.0044	881 819
37.		[1]Lacunar stroke	0.033	OR ▲1.0541	28 530
38.		[2]Total cholesterol	0.005	Beta ▼-0.0045	1 939 980

39.	rs7928675 *C11orf58* (A/**C**)	[1]Systolic blood pressure	0.006	Beta▲0.0046	1 756 920
40.		[2]Serum ApoB	0.0104	Beta▼-0.0057	436 068
41.		[2]AF-over age 65	0.005	OR▲1.1044	10 164
42.	rs7951676 *C11orf58* (G/**T**)	[1]CCS phenotypic cryptogenic large artery atherosclerosis major	0.005	OR▲1.1042	32 883
43.		[1]Systolic blood pressure	0.008	Beta▲0.0044	1 766 960
44.		[1]CCS causative large artery atherosclerosis	0.03	OR▲1.0863	32 383
45.		[1]Pulse pressure	0.049	Beta▲0.0033	879 987
46.		[2]AF-over age 65	0.005	OR▲1.1043	10 149
47.		[2]Serum ApoB	0.008	Beta▼-0.0058	436 068
48.		[2]Atrial fibrillation	0.04	OR▲1.0177	248 968

1-data obtained using the bioinformatic resource Cerebrovascular Disease Knowledge Portal (https://cd.hugeamp.org/);
2-data obtained using the bioinformatic resource Cardiovascular Disease Knowledge Portal (https://cvd.hugeamp.org/)
Effect alleles are marked in bold

Table S20: The most significant combinations of genotypes associated with the risk of developing IS

N M (1)	N K (2)	Combinations of genotypes (3)	Beta (4)	P (5)	Риск (6)
Two-locus models					
1	1.	rs10104 *C19orf53* rs2277947 *C11orf58*			
		rs10104 *C19orf53* G/G × rs2277947 *C19orf53* G/G	0.611910	0.06929	H
		rs10104 *C19orf53* A/G × rs2277947 *C19orf53* A/A	-0.053768	0.02189	L
		rs10104 *C19orf53* G/G × rs2277947 *C19orf53* A/A	0.474698	0.00843	H
		rs10104 *C19orf53* rs11666524 *C19orf53*			
		rs10104 *C19orf53* A/G × rs11666524 *C19orf53* G/G	0.43844	3.449e-07	H
		rs10104 *C19orf53* G/G × rs11666524 *C19orf53* G/G	0.60493	4.564e-03	H
		rs10104 *C19orf53* A/A × rs11666524 *C19orf53* G/A	0.46968	2.472e-06	H
		rs10104 *C19orf53* A/G × rs11666524 *C19orf53* G/A	-0.06732	2.512e-03	L
		rs10104 *C19orf53* G/G × rs11666524 *C19orf53* G/A	0.61804	9.539e-03	H
		rs10104 *C19orf53* A/A × rs11666524 *C19orf53* A/A	0.50873	9.004e-03	H
		rs10104 *C19orf53* A/G × rs11666524 *C19orf53* A/A	0.43588	9.802e-03	H
		rs3203295 *C11orf58*×rs10766342 *C11orf58*			
		rs3203295 *C11orf58* A/A × rs10766342 *C11orf58* G/A	-0.045622	0.0466187	L
		rs3203295 *C11orf58* A/C × rs10766342 *C11orf58* G/A	0.101273	0.0006061	H
Three-locus models					
		rs10104 *C19orf53*×rs2277947 *C19orf53*×rs11666524 *C19orf53*			
		rs10104 *C19orf53* A/A × rs2277947 *C19orf53* G/G × rs11666524 *C19orf53* G/G	-0.054520	1.323e-02	L
		rs10104 *C19orf53* A/G × rs2277947 *C19orf53* G/G × rs11666524 *C19orf53* G/G	0.423804	3.973e-04	H
		rs10104 *C19orf53* G/G × rs2277947 *C19orf53* G/G × rs11666524 *C19orf53* G/G	0.592495	7.927e-02	H
		rs10104 *C19orf53* A/A × rs2277947 *C19orf53* G/A × rs11666524 *C19orf53* G/G	0.510514	1.015e-06	H

		rs10104 *C19orf53* A/G × rs2277947 *C19orf53* G/A × rs11666524 *C19orf53* G/G	0.398866	2.649e-03	H
		rs10104 *C19orf53* A/A × rs2277947 *C19orf53* G/G × rs11666524 *C19orf53* G/A	0.477139	3.214e-04	H
		rs10104 *C19orf53* A/G × rs2277947 *C19orf53* G/G × rs11666524 *C19orf53* G/A	0.486830	3.724e-07	H
		rs10104 *C19orf53* A/A × rs2277947 *C19orf53* G/A × rs11666524 *C19orf53* G/A	0.421841	5.274e-03	H
		rs10104 *C19orf53* A/G × rs2277947 *C19orf53* G/A × rs11666524 *C19orf53* G/A	-0.092155	8.007e-05	L
		rs10104 *C19orf53* G/G × rs2277947 *C19orf53* G/A × rs11666524 *C19orf53* G/A	0.604014	1.141e-02	H
		rs10104 *C19orf53* A/A × rs2277947 *C19orf53* A/A × rs11666524 *C19orf53* A/A	0.494706	1.120e-02	H
		rs10104 *C19orf53* A/G × rs2277947 *C19orf53* A/A × rs11666524 *C19orf53* A/A	0.548856	1.018e-02	H
		rs10104 *C19orf53* G/G × rs2277947 *C19orf53* G/A × rs11666524 *C19orf53* A/A	0.541342	2.339e-02	H
		rs10104 *C19orf53*×rs346157 *C11orf58*×rs2277947 *C19orf53*			
		rs10104 *C19orf53* A/G × rs346157 *C11orf58* A/A × rs2277947 *C19orf53* G/G	0.352813	7.055e-02	H
		rs10104 *C19orf53* G/G × rs346157 *C11orf58* A/A × rs2277947 *C19orf53* G/G	0.597978	7.650e-02	H
		rs10104 *C19orf53* A/G × rs346157 *C11orf58* A/G × rs2277947 *C19orf53* G/G	0.467790	5.542e-08	H
		rs10104 *C19orf53* A/G × rs346157 *C11orf58* G/G × rs2277947 *C19orf53* G/G	0.696329	1.151e-02	H
		rs10104 *C19orf53* A/A × rs346157 *C11orf58* G/G × rs2277947 *C19orf53* A/A	0.500193	1.032e-02	H
		rs10104 *C19orf53* A/G × rs346157 *C11orf58* G/G × rs2277947 *C19orf53* A/A	0.512573	4.529e-03	H
		rs10104 *C19orf53* A/A× rs346157 *C11orf58* A/A × rs2277947 *C19orf53* G/A	0.533974	1.086e-04	H
		rs10104 *C19orf53* A/A × rs346157 *C11orf58* A/G × rs2277947 *C19orf53* G/A	0.504277	2.467e-05	H
		rs10104 *C19orf53* A/G × rs346157 *C11orf58* A/G × rs2277947 *C19orf53* G/A	-0.085129	6.244e-04	L
		rs10104 *C19orf53* G/G × rs346157 *C11orf58* A/G × rs2277947 *C19orf53* G/A	0.609497	1.068e-02	H
		rs10104 *C19orf53* G/G × rs346157 *C11orf58* G/G × rs2277947 *C19orf53* G/A	0.546827	2.201e-02	H
		rs8107914 *C11orf58*×rs10104 *C19orf53*×rs2277947 *C19orf53*			
		rs8107914 *C11orf58* C/C × rs10104 *C19orf53* A/G × rs2277947 *C19orf53* G/G	0.37473	1.256e-03	H
		rs8107914 *C11orf58* C/T × rs10104 *C19orf53* A/G × rs2277947 *C19orf53* G/G	0.53270	4.967e-08	H
		rs8107914 *C11orf58* T/T × rs10104 *C19orf53* G/G × rs2277947 *C19orf53* G/G	0.61207	6.987e-02	H
		rs8107914 *C11orf58* T/T × rs10104 *C19orf53* A/A × rs2277947 *C19orf53* A/A	0.49962	1.043e-02	H

		rs8107914 *C11orf58* T/T × rs10104 *C19orf53* A/G × rs2277947 *C19orf53* A/A	0.55728	9.085e-03	H
		rs8107914 *C11orf58* C/C × rs10104 *C19orf53* A/A × rs2277947 *C19orf53* G/A	0.51395	4.743e-07	H
		rs8107914 *C11orf58* C/T × rs10104 *C19orf53* A/A × rs2277947 *C19orf53* G/A	0.40683	1.609e-02	H
		rs8107914 *C11orf58* C/T × rs10104 *C19orf53* A/G × rs2277947 *C19orf53* G/A	-0.06689	4.582e-03	L
		rs8107914 *C11orf58* T/T × rs10104 *C19orf53* A/G × rs2277947 *C19orf53* G/A	-0.23099	4.095e-02	L
		rs8107914 *C11orf58* C/T × rs10104 *C19orf53* G/G × rs2277947 *C19orf53* G/A	0.60892	1.077e-02	H
		rs8107914 *C11orf58* T/T × rs10104 *C19orf53* G/G × rs2277947 *C19orf53* G/A	0.54625	2.218e-02	H
		rs10104 *C19orf53*×rs346158 *C11orf58*×rs2277947 *C19orf53*			
		rs10104 *C19orf53* A/G × rs346158 *C11orf58* T/T × rs2277947 *C19orf53* G/G	0.315922	5.177e-03	H
		rs10104 *C19orf53* G/G × rs346158 *C11orf58* T/T × rs2277947 *C19orf53* G/G	0.595130	7.810e-02	H
		rs10104 *C19orf53* A/A × rs346158 *C11orf58* T/C × rs2277947 *C19orf53* G/G	-0.284582	4.466e-03	L
		rs10104 *C19orf53* A/G × rs346158 *C11orf58* T/C × rs2277947 *C19orf53* G/G	0.539266	3.428e-08	H
		rs10104 *C19orf53* A/A × rs346158 *C11orf58* C/C × rs2277947 *C19orf53* A/A	0.497338	1.082e-02	H
		rs10104 *C19orf53* A/G × rs346158 *C11orf58* C/C × rs2277947 *C19orf53* A/A	0.540942	5.564e-03	H
		rs10104 *C19orf53* A/A × rs346158 *C11orf58* T/T × rs2277947 *C19orf53* G/A	0.517688	3.951e-07	H
		rs10104 *C19orf53* A/A × rs346158 *C11orf58* T/C × rs2277947 *C19orf53* G/A	0.419910	5.512e-03	H
		rs10104 *C19orf53* A/G × rs346158 *C11orf58* T/C × rs2277947 *C19orf53* G/A	-0.076359	1.049e-03	L
		rs10104 *C19orf53* G/G × rs346158 *C11orf58* T/C × rs2277947 *C19orf53* G/A	0.606645	1.110e-02	H
		rs10104 *C19orf53* G/G × rs346158 *C11orf58* C/C × rs2277947 *C19orf53* G/A	0.543975	2.280e-02	H
		Four-locus models			
		rs3203295 *C11orf58*×rs10766342 *C11orf58*×rs10104 *C19orf53*×rs2277947 *C19orf53*			
		rs3203295 *C11orf58* A/A × rs10766342 *C11orf58* G/G × rs10104 *C19orf53* A/A × rs2277947 *C19orf53* G/G	-0.098639	1.775e-05	L
		rs3203295 *C11orf58* A/C × rs10766342 *C11orf58* G/G × rs10104 *C19orf53* A/A × rs2277947 *C19orf53* G/G	0.542885	5.212e-08	H
		rs3203295 *C11orf58* A/A × rs10766342 *C11orf58* G/A × rs10104 *C19orf53* A/A × rs2277947 *C19orf53* G/G	0.587938	9.050e-06	H

		rs3203295 *C11orf58* A/A × rs10766342 *C11orf58* G/G × rs10104 *C19orf53* A/G × rs2277947 *C19orf53* G/G	0.491890	1.427e-06	H
		rs3203295 *C11orf58* A/C × rs10766342 *C11orf58* G/A × rs10104 *C19orf53* A/G × rs2277947 *C19orf53* G/G	0.491123	6.936e-05	H
		rs3203295 *C11orf58* A/C × rs10766342 *C11orf58* G/A × rs10104 *C19orf53* G/G × rs2277947 *C19orf53* G/G	0.564501	9.437e-02	H
		rs3203295 *C11orf58* A/C × rs10766342 *C11orf58* G/A × rs10104 *C19orf53* A/A × rs2277947 *C19orf53* A/A	0.489646	4.024e-02	H
		rs3203295 *C11orf58* A/A × rs10766342 *C11orf58* G/G × rs10104 *C19orf53* A/G × rs2277947 *C19orf53* A/A	0.503581	5.273e-03	H
		rs3203295 *C11orf58* A/A × rs10766342 *C11orf58* G/G × rs10104 *C19orf53* A/A × rs2277947 *C19orf53* G/A	0.467024	9.386e-05	H
		rs3203295 *C11orf58* A/C × rs10766342 *C11orf58* G/A × rs10104 *C19orf53* A/A × rs2277947 *C19orf53* G/A	0.505100	1.513e-03	H
		rs3203295 *C11orf58* C/C × rs10766342 *C11orf58* A/A × rs10104 *C19orf53* A/A × rs2277947 *C19orf53* G/A	0.552201	2.069e-02	H
		rs3203295 *C11orf58* A/A × rs10766342 *C11orf58* G/G × rs10104 *C19orf53* A/G × rs2277947 *C19orf53* G/A	-0.069932	1.026e-02	L
		rs3203295 *C11orf58* A/A × rs10766342 *C11orf58* G/A × rs10104 *C19orf53* A/G × rs2277947 *C19orf53* G/A	0.358433	6.609e-02	H
		rs3203295 *C11orf58* A/C × rs10766342 *C11orf58* G/A × rs10104 *C19orf53* A/G × rs2277947 *C19orf53* G/A	-0.080579	3.227e-02	L
		rs3203295 *C11orf58* A/A × rs10766342 *C11orf58* G/G × rs10104 *C19orf53* G/G × rs2277947 *C19orf53* G/A	0.703954	1.060e-02	H
		rs3203295 *C11orf58* A/C × rs10766342 *C11orf58* G/A × rs10104 *C19orf53* G/G × rs2277947 *C19orf53* G/A	0.505849	1.783e-02	H
		rs10832676 *C11orf58*×rs10766342 *C11orf58*×rs10104 *C19orf53*×rs2277947 *C19orf53*			

		rs10832676 *C11orf58* A/A × rs10766342 *C11orf58* G/G × rs10104 *C19orf53* A/A × rs2277947 *C19orf53* G/G	-0.079954	0.0031570	L
		rs10832676 *C11orf58* A/G × rs10766342 *C11orf58* G/G × rs10104 *C19orf53* A/A × rs2277947 *C19orf53* G/G	0.616016	0.0001132	H
		rs10832676 *C11orf58* A/A × rs10766342 *C11orf58* G/A × rs10104 *C19orf53* A/A × rs2277947 *C19orf53* G/G	0.595619	0.0023045	H
		rs10832676 *C11orf58* A/A × rs10766342 *C11orf58* G/G × rs10104 *C19orf53* A/G × rs2277947 *C19orf53* G/G	-0.072333	0.0302015	L
		rs10832676 *C11orf58* A/G × rs10766342 *C11orf58* G/A × rs10104 *C19orf53* A/G × rs2277947 *C19orf53* G/G	-0.083390	0.0753270	L
		rs10832676 *C11orf58* A/G × rs10766342 *C11orf58* G/G × rs10104 *C19orf53* A/A × rs2277947 *C19orf53* A/A	0.370637	0.0103647	H
		rs10832676 *C11orf58* A/A × rs10766342 *C11orf58* G/A × rs10104 *C19orf53* A/A × rs2277947 *C19orf53* A/A	0.588953	0.0005025	H
		rs10832676 *C11orf58* A/G × rs10766342 *C11orf58* G/A × rs10104 *C19orf53* A/A × rs2277947 *C19orf53* A/A	0.104163	0.0379325	H
		rs10832676 *C11orf58* A/A × rs10766342 *C11orf58* G/A × rs10104 *C19orf53* A/G × rs2277947 *C19orf53* A/A	0.512895	0.0634672	H
		rs10832676 *C11orf58* G/G × rs10766342 *C11orf58* A/A × rs10104 *C19orf53* A/G × rs2277947 *C19orf53* A/A	0.408324	0.0241389	H
		rs10832676 *C11orf58* A/A × rs10766342 *C11orf58* G/G × rs10104 *C19orf53* G/G × rs2277947 *C19orf53* A/A	0.200817	0.0303493	H
		rs10832676 *C11orf58* A/G × rs10766342 *C11orf58* G/A × rs10104 *C19orf53* G/G × rs2277947 *C19orf53* A/A	0.281887	0.0418494	H
		rs10832676 *C11orf58* A/A × rs10766342 *C11orf58* G/G × rs10104 *C19orf53* A/A × rs2277947 *C19orf53* G/A	0.135143	0.0848894	H
		rs10832676 *C11orf58* A/G × rs10766342 *C11orf58* G/G × rs10104 *C19orf53* A/A × rs2277947 *C19orf53* G/A	0.542030	0.0498278	H

		rs11826990 *C11orf58*×rs10766342 *C11orf58*×rs10104 *C19orf53*×rs2277947 *C19orf53*			
		rs11826990 *C11orf58* T/T × rs10766342 *C11orf58* G/G × rs10104 *C19orf53* A/A × rs2277947 *C19orf53* G/G	-0.1006543	1.228e-05	L
		rs11826990 *C11orf58* T/G × rs10766342 *C11orf58* G/G × rs10104 *C19orf53* A/A × rs2277947 *C19orf53* G/G	0.5440437	2.613e-08	H
		rs11826990 *C11orf58* T/T × rs10766342 *C11orf58* G/A × rs10104 *C19orf53* A/A × rs2277947 *C19orf53* G/G	0.5728892	7.075e-05	H
		rs11826990 *C11orf58* T/T × rs10766342 *C11orf58* G/G × rs10104 *C19orf53* A/G × rs2277947 *C19orf53* G/G	0.4874427	1.828e-06	H
		rs11826990 *C11orf58* T/T × rs10766342 *C11orf58* G/A × rs10104 *C19orf53* A/G × rs2277947 *C19orf53* G/G	0.6299163	6.224e-02	H
		rs11826990 *C11orf58* T/G × rs10766342 *C11orf58* G/A × rs10104 *C19orf53* A/G × rs2277947 *C19orf53* G/G	0.4727269	2.199e-04	H
		rs11826990 *C11orf58* T/G × rs10766342 *C11orf58* G/A × rs10104 *C19orf53* G/G × rs2277947 *C19orf53* G/G	0.5600905	9.738e-02	H
		rs11826990 *C11orf58* T/G × rs10766342 *C11orf58* G/A × rs10104 *C19orf53* A/A × rs2277947 *C19orf53* A/A	0.4852313	4.233e-02	H
		rs11826990 *C11orf58* T/T × rs10766342 *C11orf58* G/G × rs10104 *C19orf53* A/G × rs2277947 *C19orf53* A/A	0.4991609	5.746e-03	H
		rs11826990 *C11orf58* T/T × rs10766342 *C11orf58* G/G × rs10104 *C19orf53* A/A × rs2277947 *C19orf53* G/A	0.4625867	1.116e-04	H
		rs11826990 *C11orf58* T/G × rs10766342 *C11orf58* G/A × rs10104 *C19orf53* A/A × rs2277947 *C19orf53* G/A	0.5006764	1.687e-03	H
		rs11826990 *C11orf58* G/G × rs10766342 *C11orf58* A/A × rs10104 *C19orf53* A/A × rs2277947 *C19orf53* G/A	0.5605112	4.219e-02	H
		rs11826990 *C11orf58* T/T × rs10766342 *C11orf58* G/G × rs10104 *C19orf53* A/G × rs2277947 *C19orf53* G/A	-0.0747885	6.067e-03	L

		rs11826990 *C11orf58* T/G × rs10766342 *C11orf58* G/A × rs10104 *C19orf53* A/G × rs2277947 *C19orf53* G/A	-0.0668779	8.058e-02	L
		rs11826990 *C11orf58* T/T × rs10766342 *C11orf58* G/G × rs10104 *C19orf53* G/G × rs2277947 *C19orf53* G/A	0.6995427	1.120e-02	H
		rs11826990 *C11orf58* T/G × rs10766342 *C11orf58* G/A × rs10104 *C19orf53* G/G × rs2277947 *C19orf53* G/A	0.5014332	1.900e-02	H
Note: obtained by the MB-MDR method, taking into account correction for covariates (gender, age, smoking);<br>1 – model number of the most significant G×G interactions;<br>2 – number of the combination of genotypes included in the most significant G×G interactions;<br>3 – combination of genotypes;<br>4- beta – logistic regression coefficients for combinations of genotypes;<br>5 -p – level of significance;<br>6 - Risk: H – high, L – low					

Table S21: The most significant genotype-environment combinations associated with the risk of developing IS

N M (1)	N K (2)	Combinations of genotypes (3)	Beta (4)	P (5)	Риск (6)
Two-factor models					
1	1.	SMOKE×rs10734249 *C11orf58*			
1	2.	0 × rs10734249 *C11orf58* A/A	-0.19281	1.481e-14	L
1	3.	1 × rs10734249 *C11orf58* A/A	0.21751	3.249e-14	H
		0 × rs10734249 *C11orf58* G/A	-0.07489	2.001e-02	L
		1 × rs10734249 *C11orf58* G/A	0.16496	1.000e-05	H
		0 × rs10734249 *C11orf58* G/G	-0.16800	5.680e-02	L
		1 × rs10734249 *C11orf58* G/G	0.40873	7.999e-03	H

Three-factor models					
		SMOKE×rs10104 *C19orf53*×rs2277947 *C19orf53*			
		0 × rs10104 *C19orf53* A/A × rs2277947 *C19orf53* G/G	-0.15031	5.406e-11	L
		1 × rs10104 *C19orf53* A/A × rs2277947 *C19orf53* G/G	0.14634	4.076e-08	H
		0 × rs10104 *C19orf53* A/G × rs2277947 *C19orf53* G/G	0.39833	9.385e-05	H
		1 × rs10104 *C19orf53* A/G × rs2277947 *C19orf53* G/G	0.52311	2.998e-06	H
		0 × rs10104 *C19orf53* G/G × rs2277947 *C19orf53* G/G	0.55727	4.760e-02	H
		1 × rs10104 *C19orf53* A/A × rs2277947 *C19orf53* A/A	0.52192	1.665e-02	H
		0 × rs10104 *C19orf53* A/G × rs2277947 *C19orf53* A/A	0.34676	8.158e-02	H
		1 × rs10104 *C19orf53* A/G × rs2277947 *C19orf53* A/A	0.54539	1.234e-02	H
		1 × rs10104 *C19orf53* G/G × rs2277947 *C19orf53* A/A	0.16514	4.101e-02	H
		0 × rs10104 *C19orf53* A/A × rs2277947 *C19orf53* G/A	0.46679	1.311e-04	H
		1 × rs10104 *C19orf53* A/A × rs2277947 *C19orf53* G/A	0.53669	1.084e-05	H
		0 × rs10104 *C19orf53* A/G × rs2277947 *C19orf53* G/A	-0.14070	2.381e-07	L
		0 × rs10104 *C19orf53* G/G × rs2277947 *C19orf53* G/A	0.58622	7.147e-03	H
		1 × rs10104 *C19orf53* G/G × rs2277947 *C19orf53* G/A	0.55639	4.796e-02	H
		SMOKE×rs11024031 *C11orf58*×rs10734249 *C11orf58*			
		0 × rs11024031 *C11orf58* C/C × rs10734249 *C11orf58* A/A	-0.14576	8.035e-05	L
		1 × rs11024031 *C11orf58* C/C × rs10734249 *C11orf58* A/A	0.22241	6.387e-06	H
		0 × rs11024031 *C11orf58* C/T × rs10734249 *C11orf58* A/A	-0.14780	4.488e-06	L
		1 × rs11024031 *C11orf58* C/T × rs10734249 *C11orf58* A/A	0.15430	7.457e-05	H
		0 × rs11024031 *C11orf58* T/T × rs10734249 *C11orf58* A/A	-0.11652	9.489e-03	L
		1 × rs11024031 *C11orf58* T/T × rs10734249 *C11orf58* A/A	0.16719	1.938e-03	H
		0 × rs11024031 *C11orf58* C/T × rs10734249 *C11orf58* G/A	-0.08259	4.783e-02	L
		1 × rs11024031 *C11orf58* C/T × rs10734249 *C11orf58* G/A	0.18112	1.364e-04	H
		1 × rs11024031 *C11orf58* T/T × rs10734249 *C11orf58* G/A	0.18883	1.381e-03	H
		0 × rs11024031 *C11orf58* T/T × rs10734249 *C11orf58* G/G	-0.15930	7.551e-02	L

		1 × rs11024031 *C11orf58* T/T × rs10734249 *C11orf58* G/G	0.40308	8.895e-03	H
		SMOKE×rs3802963 *C11orf58*×rs10734249 *C11orf58*			
		0 × rs3802963 *C11orf58* G/G × rs10734249 *C11orf58* A/A	-0.20138	2.770e-15	L
		1 × rs3802963 *C11orf58* G/G × rs10734249 *C11orf58* A/A	0.21362	4.008e-13	H
		1 × rs3802963 *C11orf58* C/G × rs10734249 *C11orf58* A/A	0.25901	2.885e-02	H
		0 × rs3802963 *C11orf58* C/G × rs10734249 *C11orf58* G/A	-0.08637	9.382e-03	L
		1 × rs3802963 *C11orf58* C/G × rs10734249 *C11orf58* G/A	0.16530	1.480e-05	H
		0 × rs3802963 *C11orf58* C/C × rs10734249 *C11orf58* G/G	-0.16789	5.696e-02	L
		1 × rs3802963 *C11orf58* C/C × rs10734249 *C11orf58* G/G	0.40895	7.958e-03	H
		SMOKE×rs7928675 *C11orf58*×rs10734249 *C11orf58*			
		0 × rs7928675 *C11orf58* A/A × rs10734249 *C11orf58* A/A	-0.13750	1.341e-06	L
		1 × rs7928675 *C11orf58* A/A × rs10734249 *C11orf58* A/A	0.28049	2.356e-16	H
		0 × rs7928675 *C11orf58* A/C × rs10734249 *C11orf58* A/A	-0.18627	4.983e-07	L
		0 × rs7928675 *C11orf58* A/A × rs10734249 *C11orf58* G/A	-0.07991	2.819e-02	L
		1 × rs7928675 *C11orf58* A/A × rs10734249 *C11orf58* G/A	0.15827	1.017e-04	H
		1 × rs7928675 *C11orf58* A/C × rs10734249 *C11orf58* G/A	0.18506	3.059e-02	H
		0 × rs7928675 *C11orf58* A/A × rs10734249 *C11orf58* G/G	-0.15895	7.636e-02	L
		1 × rs7928675 *C11orf58* A/A × rs10734249 *C11orf58* G/G	0.40933	7.938e-03	H
		Four-factor models			
		SMOKE×rs11024031 *C11orf58*×rs10734249 *C11orf58*×rs6677 *C11orf58*			
		0 × rs11024031 *C11orf58* C/T × rs10734249 *C11orf58* A/A × rs6677 *C11orf58* T/T	0.48618	1.418e-02	H
		1 × rs11024031 *C11orf58* C/T × rs10734249 *C11orf58* A/A × rs6677 *C11orf58* T/T	0.38564	3.573e-02	H
		0 × rs11024031 *C11orf58* T/T × rs10734249 *C11orf58* A/A × rs6677 *C11orf58* T/T	-0.14136	2.291e-03	L
		1 × rs11024031 *C11orf58* T/T × rs10734249 *C11orf58* A/A × rs6677 *C11orf58* T/T	0.14674	7.731e-03	H
		0 × rs11024031 *C11orf58* T/T × rs10734249 *C11orf58* G/A × rs6677 *C11orf58* T/T	-0.08723	7.211e-02	L
		1 × rs11024031 *C11orf58* T/T × rs10734249 *C11orf58* G/A × rs6677 *C11orf58* T/T	0.17996	2.979e-03	H
		0 × rs11024031 *C11orf58* T/T × rs10734249 *C11orf58* G/G × rs6677 *C11orf58* T/T	-0.15070	9.753e-02	L

		1 × rs11024031 *C11orf58* T/T × rs10734249 *C11orf58* G/G × rs6677 *C11orf58* T/T	0.39785	9.662e-03	H
		0 × rs11024031 *C11orf58* C/C × rs10734249 *C11orf58* A/A × rs6677 *C11orf58* T/G	0.34052	8.600e-02	H
		1 × rs11024031 *C11orf58* C/C × rs10734249 *C11orf58* A/A × rs6677 *C11orf58* T/G	0.45871	5.878e-02	H
		0 × rs11024031 *C11orf58* C/T × rs10734249 *C11orf58* A/A × rs6677 *C11orf58* T/G	-0.17463	1.407e-07	L
		1 × rs11024031 *C11orf58* C/T × rs10734249 *C11orf58* A/A × rs6677 *C11orf58* T/G	0.12943	1.478e-03	H
		0 × rs11024031 *C11orf58* C/T × rs10734249 *C11orf58* G/A × rs6677 *C11orf58* T/G	-0.10954	1.078e-02	L
		1 × rs11024031 *C11orf58* C/T × rs10734249 *C11orf58* G/A × rs6677 *C11orf58* T/G	0.16643	5.289e-04	H
		0 × rs11024031 *C11orf58* T/T × rs10734249 *C11orf58* G/A × rs6677 *C11orf58* T/G	0.50515	5.920e-03	H
		0 × rs11024031 *C11orf58* C/C × rs10734249 *C11orf58* A/A × rs6677 *C11orf58* G/G	-0.15756	3.376e-05	L
		1 × rs11024031 *C11orf58* C/C × rs10734249 *C11orf58* A/A × rs6677 *C11orf58* G/G	0.20546	4.169e-05	H
		0 × rs11024031 *C11orf58* C/T × rs10734249 *C11orf58* A/A × rs6677 *C11orf58* G/G	0.44809	3.906e-02	H
		1 × rs11024031 *C11orf58* C/T × rs10734249 *C11orf58* A/A × rs6677 *C11orf58* G/G	0.37069	4.355e-02	H
		SMOKE×rs10734249 *C11orf58*×rs10104 *C19orf53*×rs2277947 *C19orf53*			
		0 × rs10734249 *C11orf58* A/A × rs10104 *C19orf53* A/A × rs2277947 *C19orf53* G/G	-0.18908	1.071e-10	L
		1 × rs10734249 *C11orf58* A/A × rs10104 *C19orf53* A/A × rs2277947 *C19orf53* G/G	0.22641	8.590e-10	H
		0 × rs10734249 *C11orf58* G/A × rs10104 *C19orf53* A/A × rs2277947 *C19orf53* G/G	-0.08637	3.793e-02	L
		1 × rs10734249 *C11orf58* G/A × rs10104 *C19orf53* A/A × rs2277947 *C19orf53* G/G	0.11077	2.556e-02	H
		0 × rs10734249 *C11orf58* G/G × rs10104 *C19orf53* A/A × rs2277947 *C19orf53* G/G	-0.19962	8.286e-02	L
		0 × rs10734249 *C11orf58* A/A × rs10104 *C19orf53* A/G × rs2277947 *C19orf53* G/G	0.53165	8.120e-05	H
		1 × rs10734249 *C11orf58* A/A × rs10104 *C19orf53* A/G × rs2277947 *C19orf53* G/G	0.50605	1.794e-03	H
		0 × rs10734249 *C11orf58* G/A × rs10104 *C19orf53* A/G × rs2277947 *C19orf53* G/G	0.46241	5.699e-02	H
		1 × rs10734249 *C11orf58* G/A × rs10104 *C19orf53* A/G × rs2277947 *C19orf53* G/G	0.42324	5.947e-03	H
		1 × rs10734249 *C11orf58* G/A × rs10104 *C19orf53* A/G × rs2277947 *C19orf53* A/A	0.53111	5.824e-02	H
		1 × rs10734249 *C11orf58* G/A × rs10104 *C19orf53* G/G × rs2277947 *C19orf53* A/A	0.27331	7.591e-02	H
		0 × rs10734249 *C11orf58* A/A × rs10104 *C19orf53* A/A × rs2277947 *C19orf53* G/A	0.34276	3.461e-02	H
		1 × rs10734249 *C11orf58* A/A × rs10104 *C19orf53* A/A × rs2277947 *C19orf53* G/A	0.48061	6.223e-04	H
		0 × rs10734249 *C11orf58* G/A × rs10104 *C19orf53* A/A × rs2277947 *C19orf53* G/A	0.51920	3.256e-02	H

		1 × rs10734249 *C11orf58* G/A × rs10104 *C19orf53* A/A × rs2277947 *C19orf53* G/A	0.47162	9.266e-02	H
		0 × rs10734249 *C11orf58* A/A × rs10104 *C19orf53* A/G × rs2277947 *C19orf53* G/A	-0.16086	3.790e-05	L
		0 × rs10734249 *C11orf58* G/A × rs10104 *C19orf53* A/G × rs2277947 *C19orf53* G/A	-0.15677	2.559e-03	L
		1 × rs10734249 *C11orf58* G/G × rs10104 *C19orf53* A/G × rs2277947 *C19orf53* G/A	0.50806	1.939e-02	H
		SMOKE×rs11024032 *C11orf58*×rs10766342 *C11orf58*×rs10734249 *C11orf58*			
		0 × rs11024032 *C11orf58* C/C× rs10766342 *C11orf58* G/G × rs10734249 *C11orf58* A/A	-0.21054	5.594e-13	L
		1 × rs11024032 *C11orf58* C/C× rs10766342 *C11orf58* G/G × rs10734249 *C11orf58* A/A	0.14221	1.291e-04	H
		0 × rs11024032 *C11orf58* T/C× rs10766342 *C11orf58* G/G × rs10734249 *C11orf58* A/A	0.53067	1.348e-05	H
		1 × rs11024032 *C11orf58* T/C× rs10766342 *C11orf58* G/G × rs10734249 *C11orf58* A/A	0.39955	1.397e-02	H
		0 × rs11024032 *C11orf58* C/C× rs10766342 *C11orf58* G/A× rs10734249 *C11orf58* A/A	0.56603	2.006e-02	H
		0 × rs11024032 *C11orf58* T/C× rs10766342 *C11orf58* G/A× rs10734249 *C11orf58* A/A	-0.13669	2.493e-04	L
		1 × rs11024032 *C11orf58* T/C× rs10766342 *C11orf58* G/A× rs10734249 *C11orf58* A/A	0.21002	1.597e-06	H
		1 × rs11024032 *C11orf58* T/T× rs10766342 *C11orf58* A/A× rs10734249 *C11orf58* A/A	0.24436	2.953e-02	H
		0 × rs11024032 *C11orf58* C/C× rs10766342 *C11orf58* G/G × rs10734249 *C11orf58* G/A	-0.08420	2.165e-02	L
		1 × rs11024032 *C11orf58* C/C× rs10766342 *C11orf58* G/G × rs10734249 *C11orf58* G/A	0.15061	3.587e-04	H
		0 × rs11024032 *C11orf58* T/C× rs10766342 *C11orf58* G/G × rs10734249 *C11orf58* G/A	0.61235	7.519e-02	H
		0 × rs11024032 *C11orf58* C/C× rs10766342 *C11orf58* G/A× rs10734249 *C11orf58* G/A	0.50146	6.464e-03	H
		1 × rs11024032 *C11orf58* C/C× rs10766342 *C11orf58* G/A× rs10734249 *C11orf58* G/A	0.52073	3.245e-02	H
		1 × rs11024032 *C11orf58* T/C× rs10766342 *C11orf58* G/A× rs10734249 *C11orf58* G/A	0.14732	6.886e-02	H
		0 × rs11024032 *C11orf58* C/C× rs10766342 *C11orf58* G/G × rs10734249 *C11orf58* G/G	-0.15386	8.636e-02	L
		1 × rs11024032 *C11orf58* C/C× rs10766342 *C11orf58* G/G × rs10734249 *C11orf58* G/G	0.41209	7.541e-03	H
		SMOKE×rs10734249 *C11orf58*×rs2277947 *C19orf53*×rs11666524 *C19orf53*			
		0 × rs10734249 *C11orf58* A/A × rs2277947 *C19orf53* G/G × rs11666524 *C19orf53* G/G	-0.142524	4.146e-08	L
		1 × rs10734249 *C11orf58* A/A × rs2277947 *C19orf53* G/G × rs11666524 *C19orf53* G/G	0.088724	5.113e-03	H
		1 × rs10734249 *C11orf58* G/A × rs2277947 *C19orf53* G/G × rs11666524 *C19orf53* G/G	0.201093	1.592e-05	H
		0 × rs10734249 *C11orf58* A/A × rs2277947 *C19orf53* G/A × rs11666524 *C19orf53* G/G	0.421268	1.250e-03	H
		1 × rs10734249 *C11orf58* A/A × rs2277947 *C19orf53* G/A × rs11666524 *C19orf53* G/G	0.427327	5.627e-03	H

		1 × rs10734249 *C11orf58* G/A × rs2277947 *C19orf53* G/A × rs11666524 *C19orf53* G/G	0.545942	6.090e-03	H
		0 × rs10734249 *C11orf58* A/A × rs2277947 *C19orf53* G/G × rs11666524 *C19orf53* G/A	0.417932	3.025e-03	H
		1 × rs10734249 *C11orf58* A/A × rs2277947 *C19orf53* G/G × rs11666524 *C19orf53* G/A	0.536538	5.043e-04	H
		0 × rs10734249 *C11orf58* G/A × rs2277947 *C19orf53* G/G × rs11666524 *C19orf53* G/A	0.547764	2.956e-03	H
		1 × rs10734249 *C11orf58* G/A × rs2277947 *C19orf53* G/G × rs11666524 *C19orf53* G/A	0.535033	5.230e-04	H
		1 × rs10734249 *C11orf58* A/A × rs2277947 *C19orf53* A/A × rs11666524 *C19orf53* G/A	0.528509	6.037e-02	H
		0 × rs10734249 *C11orf58* A/A × rs2277947 *C19orf53* G/A × rs11666524 *C19orf53* G/A	-0.138605	3.979e-05	L
		0 × rs10734249 *C11orf58* G/A × rs2277947 *C19orf53* G/A × rs11666524 *C19orf53* G/A	-0.128091	4.057e-03	L
		1 × rs10734249 *C11orf58* G/G × rs2277947 *C19orf53* G/A × rs11666524 *C19orf53* G/A	0.336726	3.851e-02	H
		1 × rs10734249 *C11orf58* A/A × rs2277947 *C19orf53* G/A × rs11666524 *C19orf53* A/A	0.592304	8.563e-02	H

Note: obtained by the MB-MDR method, taking into account correction for covariates (gender, age, smoking);
1 – model number of the most significant G×E interactions;
2 – number of the combination of genotypes included in the most significant G×G interactions;
3 – combination of genotypes;
4- beta – logistic regression coefficients for combinations of genotypes;
5 -p – level of significance;
6 - Risk: H – high, L – low
